# The Unfolding MD Simulations of Cyclophilin: Analyzed by Surface Contact Networks and Their Associated Metrics

**DOI:** 10.1371/journal.pone.0142173

**Published:** 2015-11-06

**Authors:** Sourav Roy, Sankar Basu, Dipak Dasgupta, Dhananjay Bhattacharyya, Rahul Banerjee

**Affiliations:** Saha Institute of Nuclear Physics, Sector 1, Block AF, Bidhannagar, Kolkata, 700064 India; Indian Institute of Science, INDIA

## Abstract

Currently, considerable interest exists with regard to the dissociation of close packed aminoacids within proteins, in the course of unfolding, which could result in either wet or dry moltenglobules. The progressive disjuncture of residues constituting the hydrophobic core ofcyclophilin from *L*. *donovani* (LdCyp) has been studied during the thermal unfolding of the molecule, by molecular dynamics simulations. LdCyp has been represented as a surface contactnetwork (SCN) based on the surface complementarity (S_m_) of interacting residues within themolecular interior. The application of S_m_ to side chain packing within proteins make it a very sensitive indicator of subtle perturbations in packing, in the thermal unfolding of the protein. Network based metrics have been defined to track the sequential changes in the disintegration ofthe SCN spanning the hydrophobic core of LdCyp and these metrics prove to be highly sensitive compared to traditional metrics in indicating the increased conformational (and dynamical) flexibility in the network. These metrics have been applied to suggest criteria distinguishing DMG, WMG and transition state ensembles and to identify key residues involved in crucial conformational/topological events during the unfolding process.

## Introduction

Currently, there is considerable interest in the role of molten globules (MG) during the process of protein folding/unfolding [1]. Two classes of MG’s have been distinguished the dry (DMG) and wet molten globules (WMG), both forms preserving native-like secondary structural elements, topology and dimensions with perturbed tertiary contacts [2,3]. The degree of perturbation in the native tertiary structure of the protein distinguishes DMG’s from WMG’s. In DMG’s, though there is dislocation (unlocking) in the tight packing of residues within the molecular interior, the consequent fraying of secondary structural elements and molecular organization is not sufficient to allow for the solvation of the hydrophobic core; in contrast to WMG’s which permits water to penetrate the interior of the molecule [4]. In other words, DMG’s involve non-native packing interactions amongst side chains coupled with the exclusion of solvent molecules from the core.

Probably, one reason why reports of DMG’s are relatively uncommon in the literature could be due to the fact that traditional probes such as tryptophan fluorescence or far UV CD generally monitors water access of previously buried groups. Experimentally, DMG’s can be unambiguously distinguished from WMG’s by the inability of the fluorescent dye ANS to bind to the core of a DMG in addition to the protection of backbone NH hydrogen’s from exchange [5]. However, given the fairly rapid, equilibrium between the locked and unlocked side chain conformations (the time constant for the locking/unlocking reaction was estimated to be about 1μs at 5°C for the villin headpiece [4]), rapid probes such as time resolved energy transfer monitored by fluorescence (FRET) [6] or 1D-NMR spectra [7,8] tracking the broadening of NMR resonance lines (corresponding to the dislocation of closed packed side chains) have been used for the detection of DMG’s. Although several instances of WMG’s have been reported amongst equilibrium folding intermediates, to date DMG’s have been unambiguously confirmed in four proteins namely, ribonuclease A [7], dihydrofolate reductase [8], single chain monellin [6] and the villin headpiece [9]. Thus, the possible universal role of DMG’s in the phenomenon of protein folding/unfolding remains to be established. Interestingly, there has also been a proposal that the side chain locking/unlocking reactions resulting in enhanced conformational flexibility of the protein could generally play a role in its catalytic function [9].

Another issue with regard to DMG’s is their relationship to the highly unstable transition state of unfolding. In an earlier theoretical study [10] the DMG was equated with the transition state species in a two step unfolding process. Subsequently, the DMG was found to be moderately stable, existing in a measurable equilibrium with the native state [11] and preceding the main transition state of unfolding. That is for a small protein like barstar [12] a DMG was found to initiate unfolding followed by a WMG, with the main transition state lying between the WMG and the unfolded state.

It is only fairly recently that extended molecular dynamics simulations of urea induced unfolding of lysozyme [13] and the SH3 domain [14] have been performed with a view to identify transient DMG intermediates in the initial stages of unfolding. Both studies indicated the presence of an early DMG intermediate(s) due to the initial penetration of urea into the hydrophobic core of the respective proteins with the exclusion of water molecules. This was attributed to the enhanced dispersion interaction [15] of urea relative to the solvent as it was the hydrophobic residues which remained preferentially dry in the DMG, probably due to their exclusive interaction with urea. Subsequently, with the progress of the simulation, solvation of the core was achieved following further conformational disruption of the protein.

The illustrative term ‘locking/unlocking’ of side chains buried within the molecular interior (initiating a DMG), possibly presupposes stereo-specific associations amongst amino acid side chains, rather in the nature of a three dimensional jigsaw puzzle [16, 17]. The unlocking of packing contacts in case of a DMG must be a very subtle effect, as in all probability the dissociated residues continue to remain in close proximity hindering solvation of the core. Previous studies with regard to the stereo-specific packing of buried amino acid side chains were productive of somewhat ambiguous results [18–22] though subsequently residues which inter-digitate with specific geometry could be identified based on fairly stringent mathematical criteria [23]. Briefly, the entire polypeptide chain was considered to be one continuous surface rather than covalently linked discrete atoms. In such a representation the surface fit or surface complementarity (S_m_) [24] of two interacting residues can be estimated along with their respective surface patches (Overlap, O_v_) buried upon association with each other. Particularly, the mathematical expression for surface complementarity (S_m_) includes a distance dependent term (Materials and Methods) rendering S_m_ especially sensitive to dislocation in stereo-specific close packing between interacting residues. Application of these two parameters S_m_, O_v_ to the side chains (surfaces) of buried residues confirmed that two interacting binary pairs exhibited highly constrained (non-random) inter-residue geometries above a certain threshold value in their mutual surface complementarity (S_m_ ≥ 0.40) and overlap (O_v_ ≥ 0.08). Thus only a subset of the total number of side chain contacts sustaining a protein fold would be expected to conserve stereo-specific jigsaw puzzle like character.

In this study, intra-molecular close packing has been represented as a network with each node representing an amino acid side chain and their mutual association as links. A link between residues has been defined in terms of the previously mentioned criteria based on the surface representation of proteins with (S_m_ ≥ 0.40) and (O_v_ ≥ 0.08). Thus, the resulting graphs could be expected to consist primarily of those interactions which strongly condition inter-residue geometry and to whom the metaphor with regard to the locking/unlocking of a three dimensional jigsaw puzzle could be justly applied. Incidentally, such graphs (surface contact networks: SCN) have been found to be assemblies of a finite number of topological units (packing motifs) which could be ordered and classified [25]. The applications of SCN’s in protein fold recognition [26] and structure validation [27] have also been established.

The representation of protein structures as networks and the application of such a paradigm has been an exceptionally fruitful research area [28–31]. Contact networks based on the proximity of main chain point atoms have been used to identify key residues in the transition state [32] and distinguish protein conformations at the point of entry and exit from the transition state region [33]. A more detailed representation using all atoms (also referred to as PSN’s: protein side chain structure networks [34]) gives the additional advantage of quantifying the strength of non-covalent side chain interactions [35] and have been used in predicting active site residues [36], rationalizing the thermal stability of thermophilic proteins relative to their mesophilic counterparts [37], tracing pathways whereby signals can be communicated between distal regions of the molecule specifically in the case of methionyl-tRNA synthetase [38], delineating subtle allosteric conformational changes upon ligand binding [39] and the identification of key residues (along with their strategic interactions) in sustaining a specific fold [40]. The utility of such graphs in dissecting conformational events in protein unfolding has also been amply demonstrated in the unfolding MD simulations of lysozyme [41] and ubiquitin [42]. In the case of ubiquitin, network based topological analysis identified modular partitions of the protein (foldons) along the unfolding pathway and key residues which served as connector hubs, stabilized early during the transition phase. A correlation has been observed in the unfolding rates of protein molecules and a topological network parameter referred to as ‘impact of edge removal per residue’ (defined as the ratio of the change of the average path length to the edge removal probability). The same study also observed no correlation between unfolding rates and the clustering coefficients of protein contact networks [43]. It has been suggested that any future definition of the (presently undefined) reaction coordinate of protein folding/unfolding should involve some function of topological network properties [33], indicating thereby the profound impact this research paradigm has made to protein science. Currently, program packages are also available which can extract wide range of network parameters from structural databases or ensembles generated by MD simulations [44].

There exists however, some differences in the methods by which side chain interaction networks have been defined to address a variety of structural and functional problems. The method adopted in this study has been extensively described above and also in the **Material & Methods** section. Another highly fruitful procedure [36] is based on the number of non-covalent interactions of side chain point atoms (which are not immediate neighbors in sequence) suitably normalized with factors estimated specifically for each individual amino acid, finally leading to the computation of an ‘interaction strength’ between interacting pairs of amino acids. An attractive feature of this algorithm allows the user sufficient flexibility to appropriately set the threshold in interaction strength so as to connect two residues by a link in the network. A somewhat related approach defines networks based on salt bridges, covalent, hydrogen bonds within proteins (‘constraint network’) to identify flexible and rigid regions of the molecule [45, 46]. A fourth method based on the explicit surface representation of side chain atoms [47, 48]computes planes between non—hydrogen atoms so as to envelop each atom by (Voronoi) polyhedra formed by the intersection of these planes. Atoms sharing a common polyhedral face are considered to be in contact and are connected by a link. The first two methods described in this paragraph are based on the distance between point atoms whereas the algorithm employed in this work and the one utilized by Joo et al. [47, 48] explicitly invoke surfaces. A few features in favor of the currently used algorithm are the inclusion of all hydrogen atoms and the provision for the user to set the thresholds in surface complementarity and overlap to generate the network. Further, utilization of surface normals in the functional definition of complementarity, make it a very sensitive indicator to perturbations in interacting side chain surfaces.

In this work the time evolution of a surface contact network (SCN) has been studied during the thermal unfolding MD simulations of cyclophilin [49] from *leishmania donovani*. Temporal patterns in the disjuncture of specific packing interactions (constituting the surface contact network) in the course of unfolding has been monitored with a view to address the following objectives:

1. To define network based metrics to quantify the degree or extent of unlocking of the amino acid side chains constituting the hydrophobic core of the protein and to ascertain whether such metrics based on surface complementarity/overlap demonstrate greater sensitivity with regard to the dislocations of side chain interactions relative to other conventional measures.
2. To examine whether the dissolution of the core involves any temporal sequence in the breakdown of specific links in the network.
3. To determine whether the unfolding simulation of cyclophilin could involve a DMG state by simultaneously monitoring the time course of these metrics and the solvent access to the hydrophobic core. In addition, an attempt has been made to define network based quantitative criteria which could serve to identify DMG like states, and perhaps serve as a general method to distinguish DMG, WMG and transition state protein conformations in an unfolding simulation.
4. And finally whether the incorporation of these metrics into multi-dimensional scaling techniques can effectively identify the transition state of unfolding.

## Materials and Methods

### Molecular Dynamics Simulation

Molecular dynamics simulation to unfold cyclophilin were undertaken at temperatures of 310, 400, 450 and 500 K for 50 ns with the initial coordinates being obtained from native crystal structure of cyclophilin (PDB Code: 2HAQ). The simulations were repeated five times at each temperature with different initial random number seeds. A cuboidal box of 78.683, 68.897 and 78.137 Åwas used to solvate the protein with the addition of 11088 TIP3P water molecules. Addition of a single Na^+^ ion ensured charge neutralization executed by the xleap in AMBER [50]. SANDER module of AMBER was utilized for energy minimization with 200 initial steps of steepest descent and 19800 steps of ABNR. Energy minimization was performed utilizing the AMBER 2002 force field, and NAMD 2.0 [51] was used for the molecular dynamics simulations. A gradient of 10 K/ps was employed to reach the simulation temperatures 310, 400, 450, 500 K (as the case may be) for a NPT ensemble. The simulation temperatures were maintained by the Langevin piston and the pressure of the system was set to 1.032 bar. Bond lengths were restricted to a tolerance level of 0.005 Å employing the SHAKE algorithm [52]. Visual molecular dynamics program (VMD 1.9.1) [53] was used for visualizing trajectories obtained from NAMD and also to extract coordinates for further ensuing computations. A time step of 1 fs was kept fixed for the velocity-verlet algorithm.

### Surface Generation, Surface Complementarity & Overlap

The van der Waals surface for the entire polypeptide chain was generated, sampled at 10 dots /Å^2^. The atomic radii were taken from the all atom molecular mechanics force field [54]. The details regarding surface generation have been described in several earlier publications [23, 25]. The entire surface of the polypeptide chain was sampled as an array of discrete dot surface points (10 dots/Å^2^) where each dot point is an area element characterized by its location (x, y, z) and the direction cosines of its normal (dl, dm, dn). Generally, residues within proteins will participate in a ‘network’ of interactions with its neighboring amino acids. The entire surface of the side chain can thus be partitioned into sub-surfaces, such that each sub-surface is in close association with the corresponding surface patch of one of its neighbors. Thus, the (previously mentioned) quantities surface complementarity (Sm) and overlap (Ov) can be used to characterize the association between two side chain surfaces of interacting amino acids [25]. Briefly, consider a residue **A**, which consists of a total of NA side chain surface points. For every side-chain dot point of **A**, its nearest neighbors (within 3.5 Å) were identified from the surrounding dot points contributed by the rest of the polypeptide chain (inclusive of both side and main chain atoms). Then, the following expression was computed:

$$S_{ab}=n_{a}.n_{b}\text{exp}\left(-wd_{ab}^{2}\right)$$ (1)

where **n**_**a**_, **n**_**b**_ are the unit normals of a dot point ‘**a**’ (on residue **A**) and those of its nearest neighbor ‘**b**’ (from residue **B**) respectively, dab is the distance between them and w is a scaling factor set to 0.5. As for two parallel surfaces, the outwardly oriented normals gives a dot product of -1.0, the direction cosines of one of the normals was inverted (made negative), so as to render S_ab_ positive (with a maximum value of 1.00) in case of favorable alignment of surfaces. Thus, for the set of points N_AB_, (dot points on **A** which have found points on the side chain **B** as nearest neighbors) median of distribution {Sab} is defined as the surface complementarity S_m_^A→B^. The corresponding overlap between residues A and B is defined as,

OvA→B= NABNA (2)

where, as mentioned previously *N*_*AB*_ is the number of side chain surface points residue A which have found nearest neighbors amongst the side chain surface points on residue B and *N*_*A*_ is the total number of side chain surface points of residue A. Notably, the maximum possible value attained by both Sm and Ov are 1.00.

### Protein Surface Contact Networks (SCN)

Generally, two point atoms are said to be in ‘contact’ based on purely distance criteria. Since therepresentation of atoms has been expanded to surfaces the criteria for two residues to be in contact based on S_m_ and O_v_ are as follows. S_m_ and O_v_ are generally non-commutative, that is S_m_^A→B^ or O_v_^A→B^ does not necessarily equalS_m_^B→A^ orO_v_^B→A^ respectively. Thus, two residues **A**,**B** are said to be in ‘contact’ when bothS_m_^A→B^, S_m_^B→A^ andO_v_^A→B^, O_v_^B→A^ simultaneously satisfy the cutoffs (are greater than or equal to) 0.4 and 0.08 respectively. These cutoffs were chosen on the basis of a previous work [23] which showed that these criteria (in S_m_ and O_v_) led to highly constrained inter-residue geometries for interacting binary pairs of amino acids. Briefly, all interacting residues belonging to a specific binary pair (say, Val-Leu) were extracted from a database of protein structures and distributed in 4 bins based on their mutual S_m_ and O_v_ values. For any pair the inter-residue geometry was characterized in terms of specific angles defined between the coordinate axes rigidly fixed on each residue. It was found that the distribution of these angles deviated significantly from a random distribution in case of the fourth bin which constituted of binary pairs with high S_m_ (≥ 0.40) and O_v_ (≥0.08) respectively. In the context of protein surface contact networks (SCN), a node stands for an amino acid side chain and two nodes are connected by an edge, when they are in surface contact (as defined above). Henceforth, all such networks will be referred to as protein ‘Surface Contact Networks’ (SCN). Like all other networks SCN’s can be represented in terms of adjacency matrices with elements (a_ij_) equal to one when two nodes are connected by an edge and zero otherwise. Figures ofSCN’swere drawn using Cytoscape v3.2.0. [55].

### Metrics defined on Surface Contact Networks: Disnet, Dlf, Persistence and Persf

SCN’s were constructed based on the coordinates of LdCyp (snapshots) at various stages of the unfolding simulation at different temperatures. The SCN for any snapshot was represented as an adjacency matrix, where the elements of the matrix a_ij_ could assume values either 0 or 1 (1 denoting the presence of an interaction between residues i and j as defined above). Only residues constituting the hydrophobic core of the protein were considered in the construction of the SCN adjacency matrices. The distance (*Disnet*)between any two such adjacency matrices was determined by counting the number of links present in one and absent in other, and normalizing by N(N-1)/2 where N is the total number of residues composing the hydrophobic core of the protein.

$$Disnet(A,A`)=\frac{\sum_{i=1}^{N}\sum_{j=i+1}^{N}|A_{ij}-A{`}_{ij}|}{N(N-1)/2}$$ (3)

Where Aij and A’ij are the matrix elements of two adjacency matrices (A, A’) corresponding to two SCN’s (from their corresponding snapshots) and N(N-1)/2 is the maximum number of possible links. Thus *Disnet (A*, *A’)* essentially estimates the dissimilarity (‘distance’) between two adjacency matrices by counting the unique number of links in each matrix divided by the maximum number of possible links in the network. In other words a *Disnet* value of 0.4 would imply that 40% of all the possible links in the surface contact network of the hydrophobic core are unique to either one or the other matrix. Conversely 60% of the links have identical status in both the matrices.

Based on *Disnet* two more related measures ‘dlf’ and ‘persf’ were defined in order to assess the degree of stability or fluctuations in the SCN’s. Generally the degree of stability associated with a link should be directly proportional to the number of times the link persists in the network (corresponding to each snapshot in the course of the simulation). As has been mentioned previously, each simulation run at a specific temperature (310, 400 K) was for a total of 50 ns. Discarding the first 2 ns, every simulation run was partitioned into contiguous intervals of 2 ns, henceforth to be referred as epochs. Within an epoch, adjacency matrices were constructed pertaining to snapshots sampled every 10 ps leading to 200 snapshots (and their corresponding adjacency matrices) per epoch. Thus the ‘persistence’ of a link between residues i and j in an epoch was defined as the number of times the link was detected in the snapshots (spanning the epoch) divided by the total number (200) of snapshots. Thus,

$$P_{ij}=\sum_{k=1}^{Ns}A_{ij}/N_{s}$$ (4)

where, A_ij_ are elements of the SCN adjacency matrices, the summation being over snapshots (k) and Ns equals 200.

The overall assumption in this work is that a ‘locked’ interaction between two side chains simultaneously satisfies the stated criteria in overlap and surface complementarity thereby linking two nodes in the SCN. As a dynamic equilibrium could exist between the ‘locked’ and ‘unlocked’ state between two residues, (i and j) the overall interaction in an epoch was considered to be locked when the link was found to persist in more than 40% of the snapshots, or in other words when P_ij_ ≥ 0.40. Thus, a new matrix P’ was constructed (referred to as the persistence matrix) such that P’_ij_ = 0 when P_ij_< 0.4 and P’_ij_ = 1 when P_ij_ ≥ 0.40, which represented relatively stable regions of the network. The metric *Disnet* could again be used to estimate the distance between persistence matrices, and its values when so applied was called ‘persf’. Thus, persf estimates the dissimilarity between two networks which represent the relatively stable links in the SCN’s.

In order to estimate the degree of fluctuation (instability) associated with SCN’s in an epoch the metric *Disnet* was estimated for every pair of (SCN) adjacency matrices associated with snapshots spanning the epoch. Then, somewhat analogous to the measure ‘alf’, [56] the average <*Disnet*> over all such pairs ([200x199]/2!) was defined as dlf. Since higher values in *Disnet* implies enhanced dissimilarity between SCN adjacency matrices, high dlf for an epoch would indicate increased divergence amongst the SCN’s spanning the epoch, as a consequence of higher fluctuations in the SCN’s. All data pertaining to *Disnet* was smoothed by a 45 point adjacent averaging method.

### Fractional Native Contacts (Q) and Links (Q_L_)

Initially, 35 native contacts in the core of LdCyp were identified from the crystal structure of LdCyp with a distance cutoff of 3.8 Å (PDB id: 2HAQ). 25 native links (also from the crystal structure) were calculated from the surface contact network. The fraction of native links in the core Q_L_ was calculated for each snapshot at different temperatures along with the fraction of native contacts (Q). For both Q and Q_L_ the data was smoothed by 45 points adjacent averaging.

### Solvent Accessible Surface of Core Residues

The solvent accessibility of amino acid residues in the protein molecule was estimated using a probe radius of 1.4 Å. To provide an estimate of solvent access to the hydrophobic core (constituted of 24 residues mentioned previously) in a snapshot, the side chain solvent accessible areas (Å^2^) of the core residues were first summed (SASC) and then normalized by the average of the same measure obtained from the snapshots of the native simulation at 310 K.

Thus,

SASCN =  SASC of snapshot/ Average SASC from snapshots at 310 K (5)

### Secondary Structural Content

The secondary structural content for any snapshot was estimated by STRIDE [57] and the status of residues constituting the helices (H1, H2) and the 8 ß strands composing the barrel (as found in the native crystal structure, PDB id: 2HAQ) was monitored. A total of 79 residues out of 166 residues of the protein made up the total secondary structural content of native LdCyP crystal structure (with 57 residues in the ß barrel; H1: 13 residues, H2: 9 residues). Thus, fractional secondary structural content for any snapshot was defined as the fraction of these 79 residues found to reside in their native secondary structural elements, either helices or strands. Average fractional secondary structural content within an epoch of 2 ns, SSC_epoch_ was determined by averaging fractional secondary structural content over all the snapshots (200) within the epoch.

### Free Energy Landscape

The free energy landscapes for the simulations at different temperatures as a function Q_L_ and SASCN were computed by means of a histogram analysis [58, 59, 60] wherein the Q_L−_ SASCN plots were divided into 20x20 bins of equal size and the frequency of snapshots was calculated in each bin. Then following Bhattacharyya et al. [58] Helmholtz free energy was estimated by calculating the probabilities for each bin and reference (cell) assigned to the cell containing maximum number of snapshots.

ΔAi= −RTln(PiPref) (6)

R is ideal gas constant, T is temperature in Kelvin, and *P*_*i*_ and *P*_*ref*_ are the probabilities of finding the system in the ith cell and the reference cell, respectively. The absolute Helmholtz free energies were then given by

Ai= Aref−RTln(PiPref) (7)

Aref= −RTln(NrefNtot) (8)

where *N*_*ref*_ is the number of snapshots in the reference cell and *N*_*tot*_ the total number of snapshots in the plot.

### Classical Multi-Dimensional Scaling

Methods of classical multi-dimensional scaling were adopted to map ‘distances’ between snapshots based on *Disnet* to three dimensional coordinates. Initially, the entire 50ns simulation at a specific temperature was sampled in 100 ps intervals leading to 500 snapshots in all. Computing the ‘distance’ (with respect to the metric *Disnet*) for every pair of snapshots led to the construction of a 500 x 500 matrix (D: elements d_ij_), where the matrix element d_ij_ gives the metric (*Disnet*) distance between the i^th^ and j^th^ snapshots. The calculation was also repeated at 200 ps sampling leading to a 250 x 250 matrix. Then following Borg and Groenen [61] the eigenvalues and eigenvectors were computed for the matrix.

$$B=-\frac{1}{2}JP^{\left(2\right)}J$$ (9)

Where for P^2^, every element P_ij_ = D_ij_^2^ and J = I– 1/n [L] where ‘I’ is a n x n unit matrix, ‘n’ is the number of snapshots and [L] is a n x n matrix where every element is unity. The eigenvectors corresponding to the three largest eigenvalues were selected and the three dimensional coordinates for each snapshot was obtained upon suitably combining the eigenvectors subsequent to multiplying them by the square root of their respective eigenvalues.

## Results and Discussions

### Comparison of network based metrics with conventional measures

**Fig 1:**
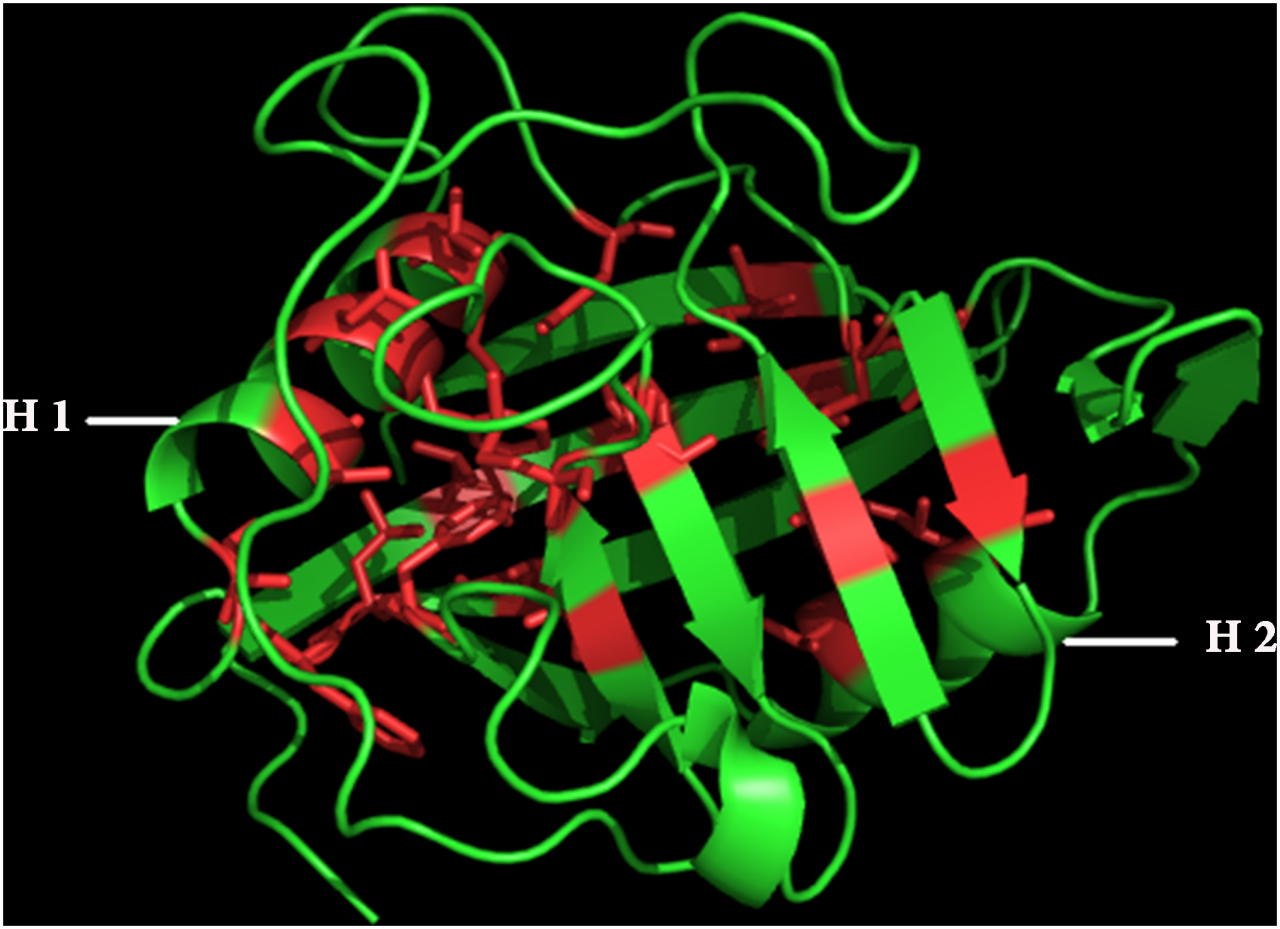
Three dimensional structure of cyclophilin from *Leishmania Donovani* (LdCyp) representing the core residues. Crystal structure of cyclophilin from *leishmania donovani* (PDB id: 2HAQ) with the centrally located β-barrel flanked by two helices H1 and H2. The core residues are marked in red in the stick mode. The figure was generated using Pymol. [62]

Thermal unfolding MD simulations have been performed on cyclophilin from *L*.*donovani* (LdCyp) as a model system. Cyclophilins are a ubiquitous class of peptidyl-prolyl cis-trans isomerases, also known to be the intra-cellular receptor for the immunosuppressive drug cyclosporine A (CsA), regularly used in organ transplants. LdCyp is a single domain protein composed of an 8-stranded β-barrel with two helices located at the either end (Fig 1).

The location of the two helices (designated H1 and H2) with respect to the barrel effectively blocks the solvent accessibility of the only hydrophobic core of the molecule, located in the interior of the barrel. Previous work [63] experimentally confirmed the presence of an (possible WMG type) equilibrium intermediate in the unfolding of LdCyp by guanidium hydrochloride. Preliminary MD simulations performed at 310, 400 and 450 K indicated the tendency of the helices to partially unwind and adopt non-native geometries with respect to the core, despite the persistence of a relatively stable β-barrel.

**Fig 2:**
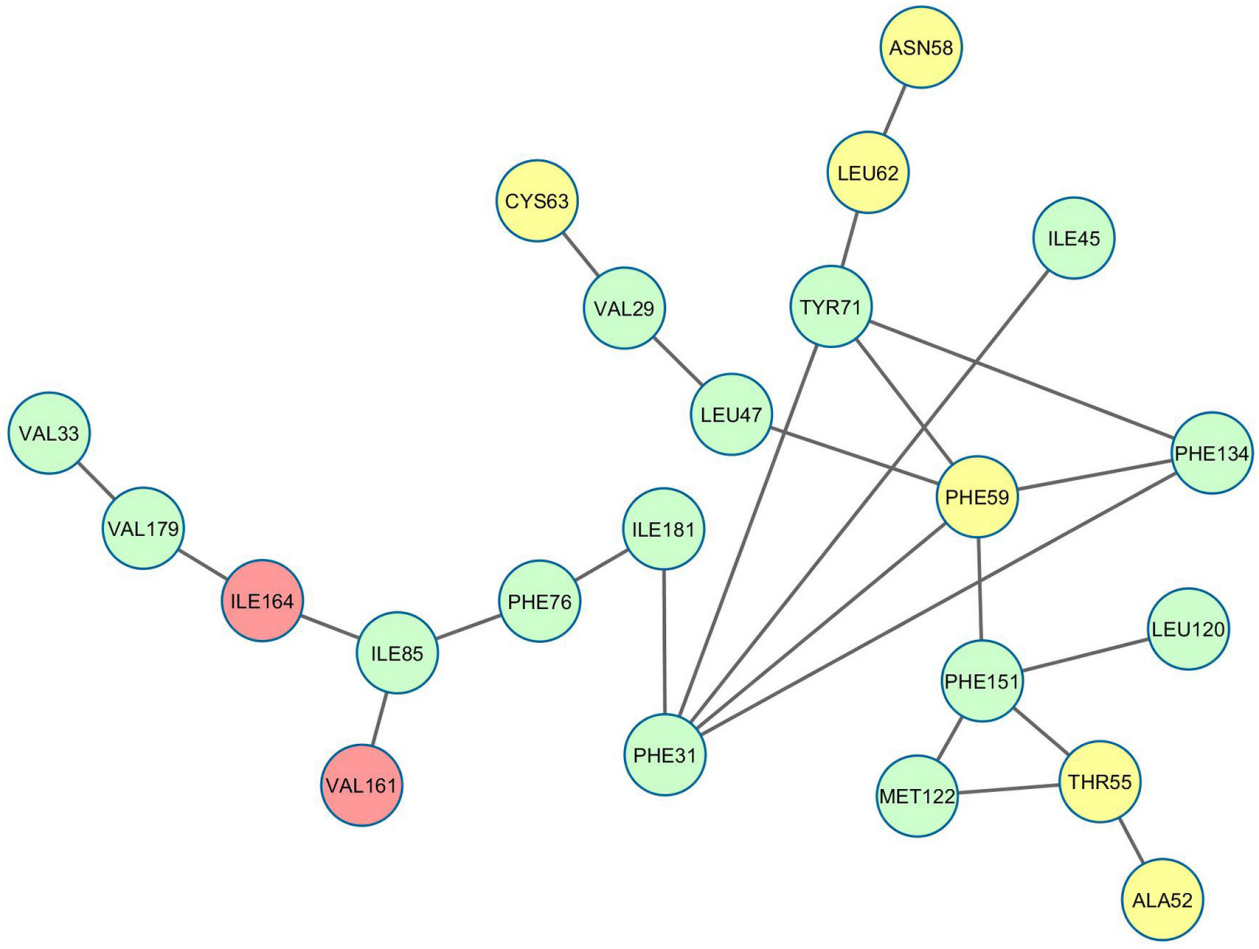
Surface contactnetwork of LdCyp (core residues) based on the crystal structure. Residues (nodes) located on helix H1 have been colored yellow, helix H2: red and β strands: green. The diagram was drawn using Cytoscape v 3.2.0 [55]

To start with, a surface contact network (SCN) was constructed based on the native crystal structure of LdCyp (PDB id: 2HAQ), only considering amino acid side chains of the unique hydrophobic core of the molecule. The hydrophobic core of LdCyp is constituted of 24 residues, of which 8 are contributed by flanking helices (H1, H2), while the rest predominantly come from the β-strands encompassing the core. As has been mentioned previously, two nodes of the surface contact network (representing amino acid side chains) are connected by a link when their mutual surface complementarity and overlap (Materials and Methods) exceeds (or is equal to) 0.4 and 0.08 respectively. As such binary interactions generally arise due to stereo-specific association of amino acid side chains with constrained inter-residue side chain geometries, a link between two nodes of the network, could be considered to represent a ‘locked’ state, analogous to the adjacent pieces of a three dimensional jigsaw puzzle. Thus, the time evolution of SCN’s spanning the entire core and the time course of its disintegration could prove to be a convenient global descriptor of the ‘unlocking’ of the protein core, upon unfolding. During the entire study only the residues constituting the core as defined by the native crystal structure were taken into consideration. Amongst the 24 core residues of cyclophilin, a set of 25 native links (Fig 2) were determined (Table A in S1 File) and were classified into core-core (S1, 11 links), helix 1-core (S2, 9 links) and helix 2-core (S3, 3 links). The remaining two links were intra-helical contacts.

A set of metrics characterizing the topological state and dynamic fluctuations in the network were then defined and compared with traditional measures such as Cα RMSD and Q (fractional native contacts). Analogous to Q, the fraction of native links (Q_L_) with respect to the crystal structure was computed for every snapshot. However, the most fundamental metric *Disnet* was defined as the number of uncommon links between two snapshots normalized by the maximum number of possible links in the core consisting of 24 nodes (24 x 23 /2). In every application of *Disnet*, the SCN’s derived from the snapshots were compared with the network based on the native crystal structure. Although, both measures (Q_L_ and *Disnet*) can assume a maximum value of 1.00; in the former it implies that all the native links have been detected in the snapshot whereas in the latter it is indicative of the fact that the two sets of links (from the crystal structure and snapshot) are two disjoint sets, whose elements sum to the maximum possible links which can be realized in the core. To estimate network fluctuations in the course of unfolding, the entire simulation block (of 50 ns) was divided into non-overlapping, contiguous intervals of 2 ns (henceforth referred to as epochs). Each epoch was sampled every 10 ps (leading to 200 snapshots/epoch) and a new persistence matrix constructed such that its elements P’_ij_ = 1, when the link between residues i and j were observed in more than (equal to) 40% of the snapshots spanning the epoch. In other words an interaction between residues i and j was considered to be locked within an epoch when the corresponding link was observed in more than 40% of the snapshots. Each persistence matrix derived from any epoch was compared to a persistence matrix observed upon averaging an entire simulation block at 310 K (310KSIM1) via *Disnet*, though in this usage the resultant values were referred to as ‘persf’. Lastly, the measure dlf (analogous to alf) was the average of all *Disnet* values obtained between every pair of snapshot within an epoch.

Each simulation run was for 50 ns and was conducted five times at temperatures of 310, 400, 450 and 500 K making for a total of 1 μs simulation time. The first 2 ns for every simulation were not considered for any further calculation. To start with the behavior of metrics Q_L_ and *Disnet* were compared with the conventional parameters Cα RMSD and Q. The Cα RMSD was calculated twice (with respect to the crystal structure 2HAQ), one for the entire polypeptide chain (RMSD_ALL) and again only for residues constituting the hydrophobic core (RMSD_CORE) (Table 1). As expected, the average RMSD for the entire protein was more than double the value obtained for the core (Table 1). The simulation at 310 K was considered to be ‘native’ and the average (RMSD_ALL) (from 3–50 ns) rose in increments from 310 to 500 K (Table 1).

**Table 1:** Average Cα RMSD of simulations considering all residues (SIM1_ALL) of LdCyp and with only the core residues (SIM1_CORE) from simulation sets SIM1. The standard deviations are given in parentheses.

Temperature (K)	<Cα RMSD>	
	All	Core
310	1.35 (0.12)	0.53(0.06)
400	2.76 (0.47)	1.08 (0.33)
450	3.91(0.85)	1.29(0.32)
500	5.20(1.12)	2.69(0.81)

**Fig 3:**
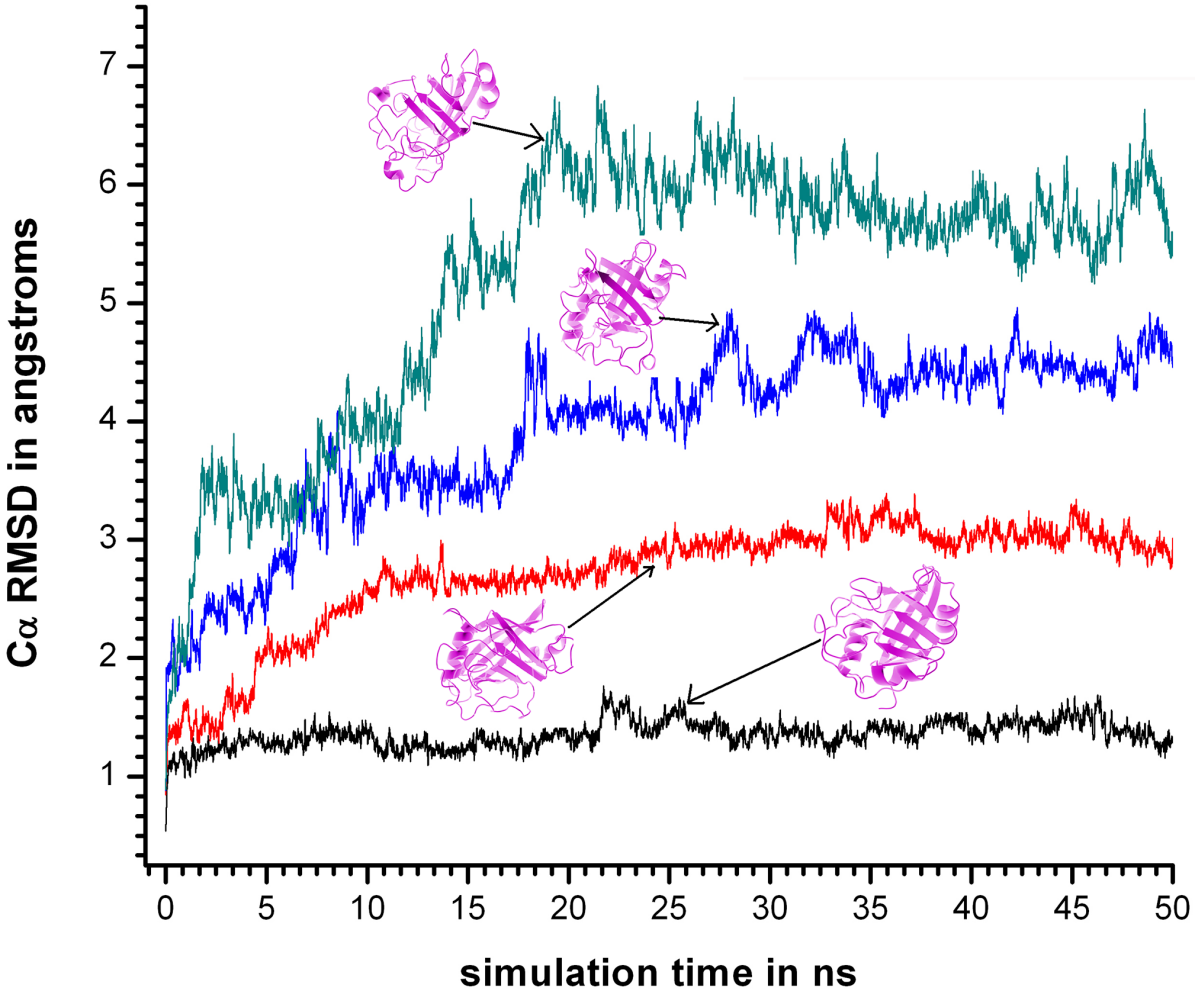
Cα RMSD at different simulation temperatures consisting of all residues. Cα RMSD for simulation sets 310KSIM1 “black solid line”, 400KSIM1 “red solid line”, 450KSIM1 “blue solid line”, and 500KSIM1 “green solid line”, involving all the residues, calculated using the crystal structure of cyclophilin as a reference with the ribbon diagram of representative snapshots in magenta.

Visual examinations of the snapshots revealed that the rise in RMSD_ALL at 400 and 450 K (Fig 3) were primarily due to the relative detachment of the helices (first H2 followed by H1) from their native positions with respect to the barrel.

**Fig 4:**
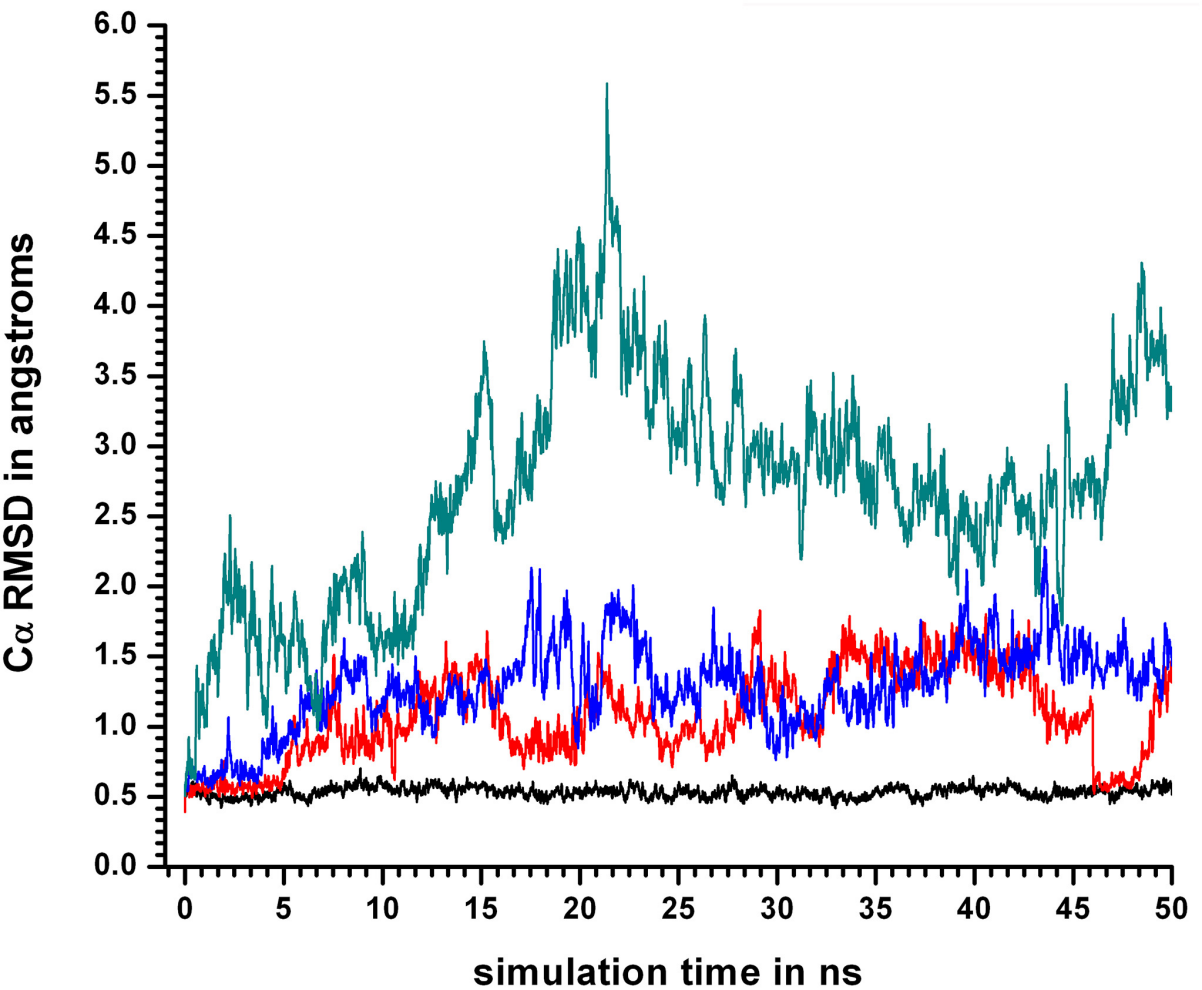
Cα RMSD at different simulation temperatures with only the core residues. Cα RMSD for simulation sets 310KSIM1 “black solid line”, 400KSIM1 “red solid line”, 450KSIM1 “blue solid line”, and 500KSIM1“green solid line”, involving only the 24 hydrophobic core residues, calculated using the crystal structure of cyclophilin as a reference. Cα RMSD is relatively insensitive to subtle distortions in the core.

However, in contrast to RMSD_ALL a relatively insignificant difference in the average values of RMSD_CORE were observed between 400KSIM1 and 450KSIM1 (Fig 4), in confirmation of a previous observation that Cα RMSD over the entire polypeptide could be a misleading metric as it could be predominantly influenced by peripheral distortions in the protein rather than in the core.

For 400KSIM1 and 450KSIM1 the surge in the values of RMSD_ALL were in the first 10–15 ns of the simulation (Fig 3) and only in 500KSIM1 did both RMSD_ALL and RMSD_CORE rise simultaneously with almost comparable values (in the first 20 ns), indicative of the complete unraveling of the structure.

Similar values of average Cα RMSD was observed for both RMSD_ALL and RMSD_CORE (Table B in S1 File, Figure A in S1 File) for other sets of simulations at 310 (SIM2–SIM5), 400 (SIM2 –SIM5) K. At 450 K relatively greater fluctuations were observed in RMSD_ALL and RMSD_CORE with significant divergence post 35ns (in 450KSIM2-SIM5), whereas for 500 K simulation sets (500KSIM2-5) RMSD_CORE were close to the RMSD_ALL values up to 15 ns significantly deviating thereafter (Figure A in S1 File). Additionally, RMSF calculations were performed to assess the fluctuations the core residues at different temperatures (Figure B in S1 File, Table C in S1 File) and as expected exhibited a rise with temperature indicative of rearrangement of core residues.

It was most instructive to compare the relative sensitivities of Q (fraction of native contacts in the core) with Q_L_ (fraction of native links in the network). The average <Q> (from 3–50 ns) was almost identical for 310KSIM1, 400KSIM1 on one hand and 450KSIM1, 500KSIM1 on the other (Table 2).

**Table 2:** Fraction of the native contacts (Q) and native links (Q_L_) at different simulation temperatures. The standard deviations are given in parentheses. The increased sensitivity of Q_L_ compared to Q is evident from the table.

Simulations	<Q>	<Q_L_>
310KSIM1	0.90(0.02)	0.73(0.08)
400KSIM1	0.90(0.02)	0.68(0.09)
450KSIM1	0.77(0.11)	0.41(0.15)
500KSIM1	0.71(0.10)	0.26(0.14)

**Fig 5:**
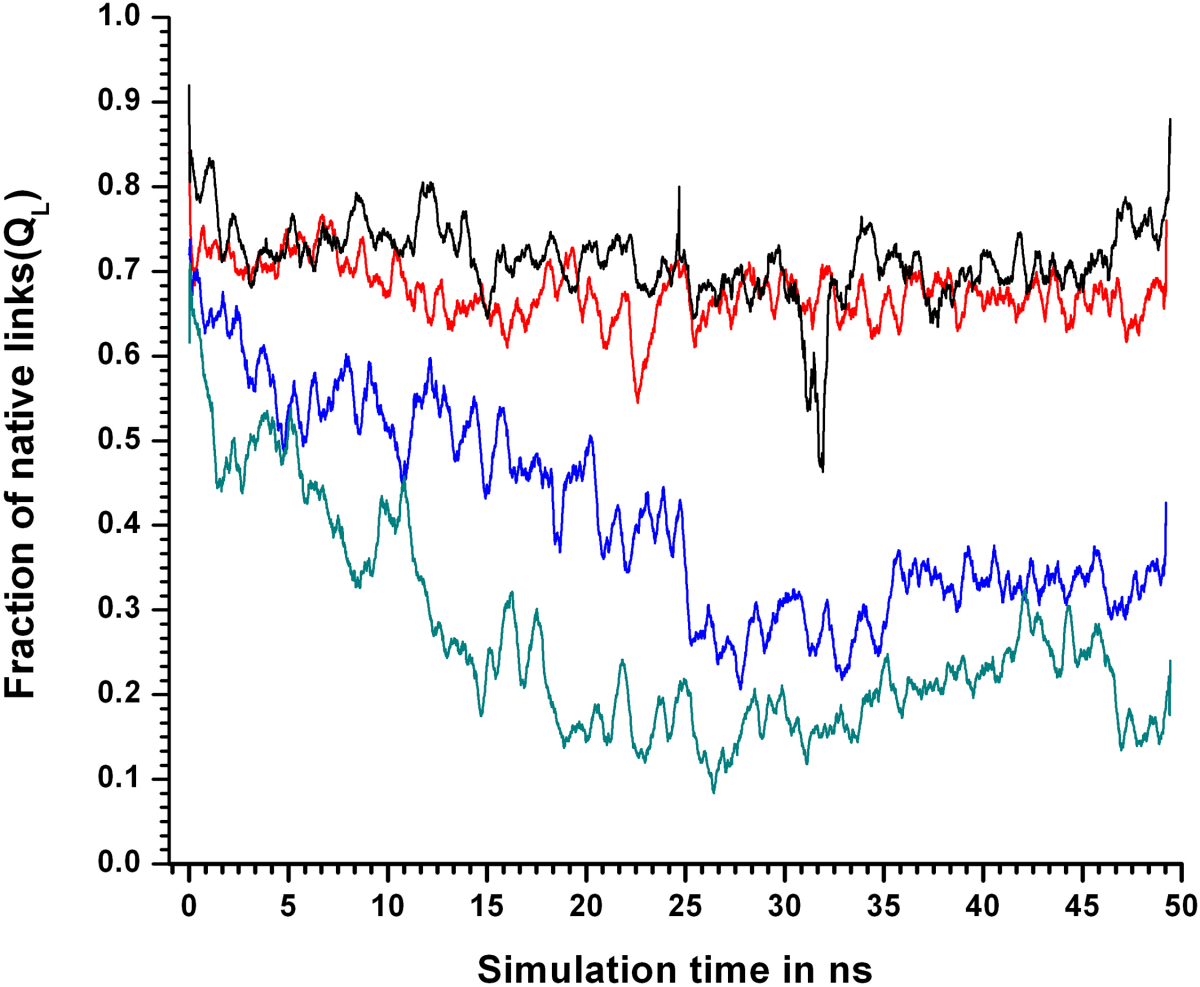
Fraction of native links at different simulation temperatures. Fraction of native links (Q_L_) for simulations 310KSIM1 “black solid line”, 400KSIM1 “red solid line”, 450KSIM1 “blue solid line”, and 500KSIM1 “green solid line”, of the 24 hydrophobic core residues calculated with the crystal structure of LdCyp (PDB id: 2HAQ) as a reference. At 450 K decline in Q_L_ values begins from the very start of the simulation.

<Q_L_> however declined with every elevation in temperature most significantly between 400KSIM1–450KSIM1 and 450KSIM1–500KSIM1 (Fig 5).

**Fig 6:**
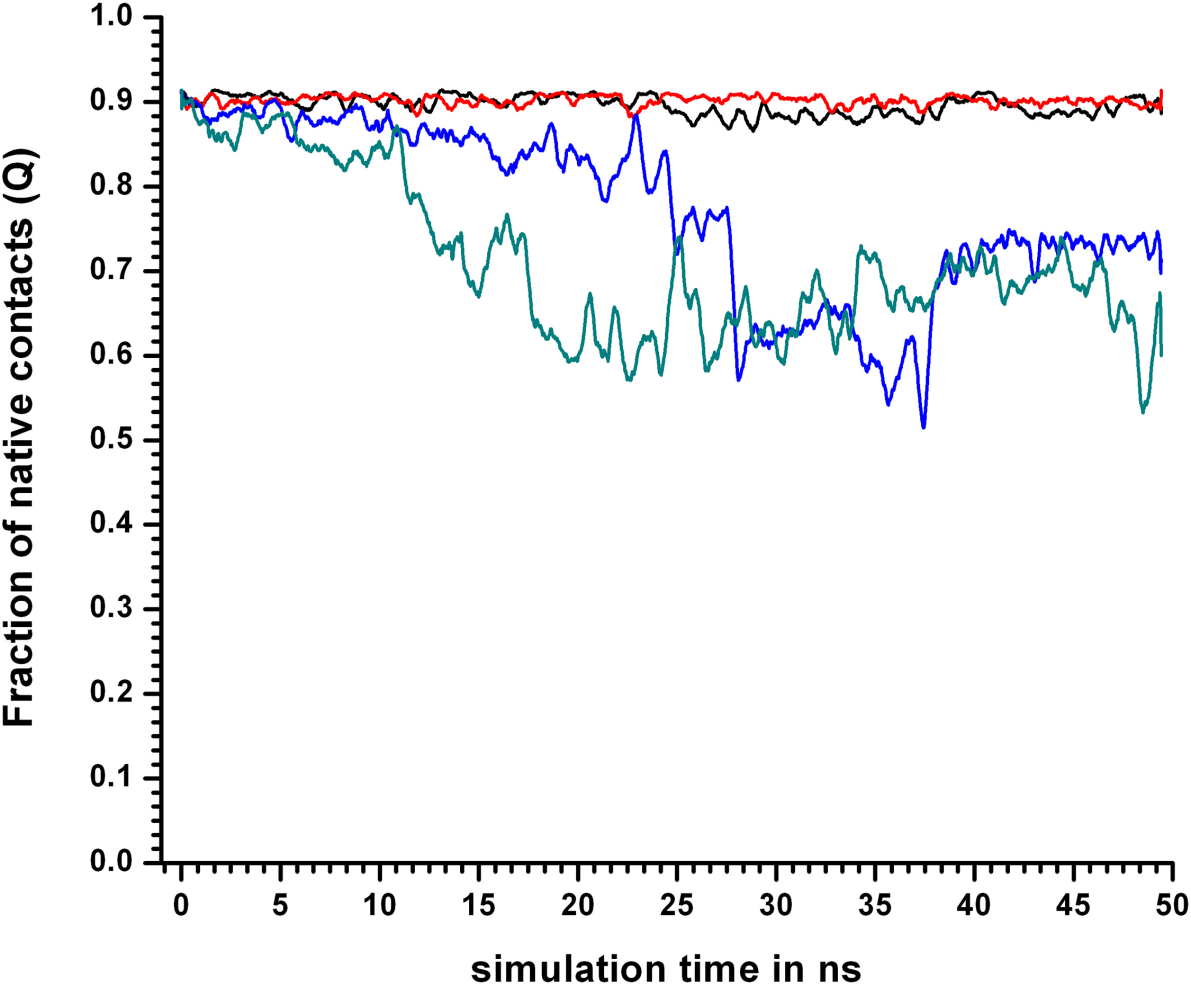
Fraction of native contacts (Q) at different simulation temperatures. Fraction of native contacts (Q) for simulations 310KSIM1 “black solid line”, 400KSIM1 “red solid line”, 450KSIM1 “blue solid line”, and 500KSIM1 “green solid line”, of the 24 hydrophobic core residues calculated with the crystal structure of LdCyp (PDB id: 2HAQ) as a reference.

Even at 400KSIM1 there was a mild decrease in Q_L_ with respect to the native simulation (at 310KSIM1). At 450KSIM1, Q maintained a steady value of about 0.9 for the first 20 ns, abruptly falling to about 0.7 (Fig 6) thereafter.

**Fig 7:**
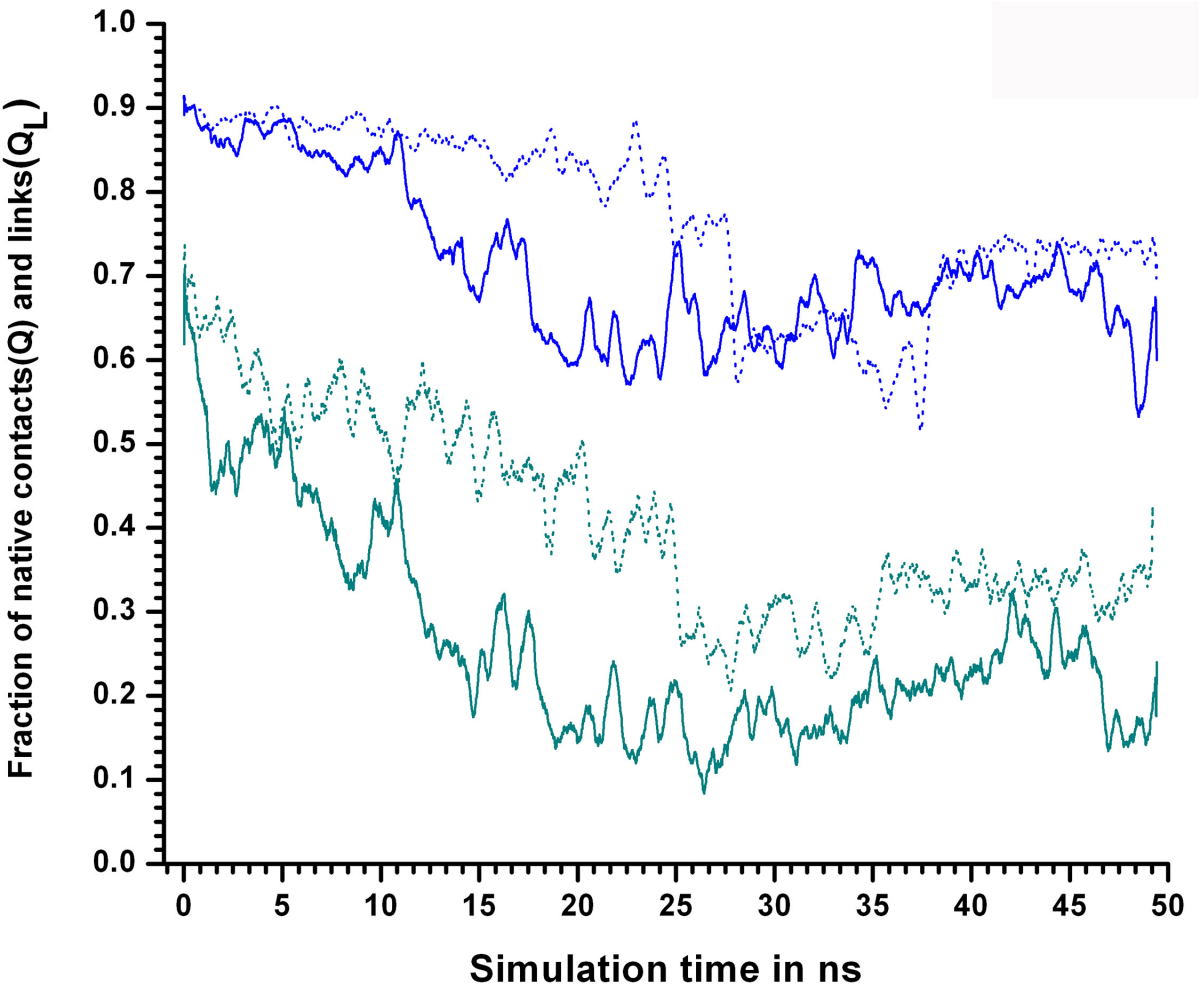
Comparison of Q and Q_L_ in terms of sensitivity at 450 and 500K. Comparison between Q and Q_L_ for simulation sets 450KSIM1 and 500KSIM1. The dotted line “blue line” represents Q for 450KSIM1 whereas the “solid blue line” for Q_L_. Similarly for 500 K Q, Q_L_ are represented by “dotted green line”, “solid green line” respectively. At both 450, 500 K the drop in Q_L_ values begins prior to Q indicating it’s greater sensitivity.

In contrast, (from the same simulation 450KSIM1), the decline in Q_L_ was more gradual and in progress from the very start of the simulation (falling from 0.7 to 0.3 in 0–25ns). Thus, it seemed evident that links as defined in SCN’s were more sensitive to perturbations (unlocking) in the packing interactions compared to Q (Fig 7). The difference in the sensitivities between Q and *Disnet* were also rather stark especially at 400, 450 K (Figure C in S1 File). It was also evident that RMSD_CORE was the least sensitive indicator of decisive conformational events in the core.

The time course of both Q_L_ and *Disnet* appeared to be somewhat similar though with some differences. Average <*Disnet*> (from 3–50ns) recorded a characteristic increase with rise in temperature (Table 3).

**Table 3:** <*Disnet*> values for different simulation temperatures (310KSIM1, 400KSIM1, 450KSIM1 and 500KSIM1) calculated with native crystal structure (2HAQ) as baseline averaged over the entire simulation block of 50 ns with the standard deviations given in parentheses.

Simulations	<*Disnet*>
310KSIM1	0.46(0.07)
400 KSIM1	0.51(0.08)
450 KSIM1	0.70(0.12)
500 KSIM1	0.83(0.11)

**Fig 8:**
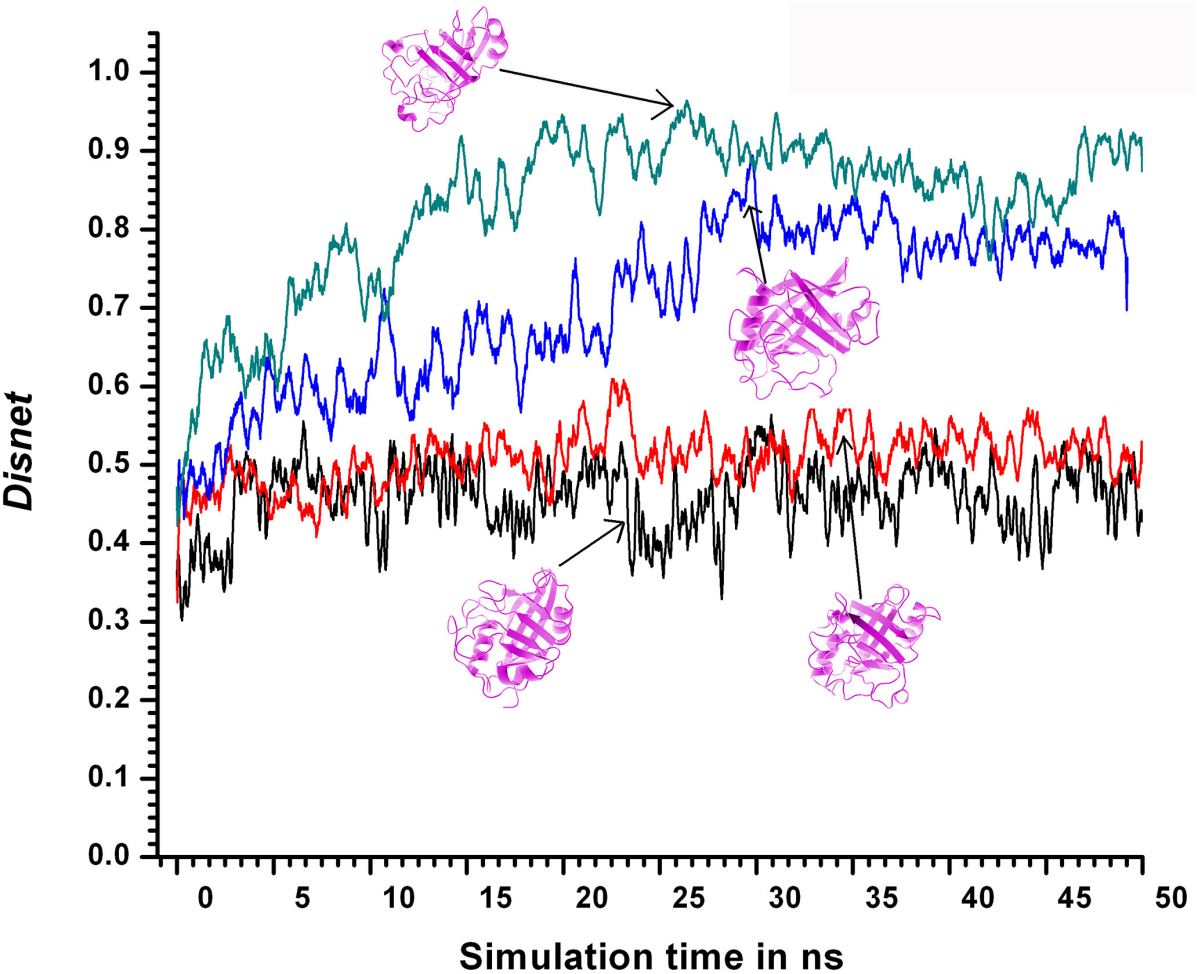
*Disnet* values at different simulation temperatures. *Disnet* values between the Surface Contact Networks (SCNs) of the snapshots and the SCN derived from the crystal structure (2HAQ) plotted as a function of simulation time for the simulations 310KSIM1” black solid line”, 400KSIM1 “red solid line”, 450KSIM1 “blue solid line”, 500KSIM1 “green solid line”. Residues constituting the hydrophobic core of LdCyp alone were considered in the construction of the SCNs with the structure of representative snapshots for 310, 400, 450 and 500 K (SIM1) in magenta (ribbon diagram).

For 310KSIM1 <*Disnet*> was 0.46 ± 0.07, implying that on an average 54% of the links in the core were identical to those found in the native crystal structure. Similar to Q_L_ a marginal increase in <*Disnet*> was observed at 400KSIM1 (0.51 ± 0.08) relative to 310 K. At 450KSIM1 there was a gradual increase in *Disnet* from 5–20ns, followed by a comparatively abrupt rise between 20–30ns (Fig 8). The increase in the interval of 20–30ns appeared to be more marked in *Disnet* compared to Q_L_. For 500KSIM1 the final saturated value <*Disnet>* was0.83. In the native simulation at 310KSIM1 and also 400KSIM1 the hydrophobic core of the molecule was relatively compact with the flanking helices assuming near native geometry with respect to the core reflected in the low *Disnet* values (~0.5) (Fig 8).

With rise in simulation temperature at 450KSIM1 the core tended to become more labile (around 25ns) with increasingly relaxed packing between helix H1, helix H2 and the core during which *Disnet* approached a value of 0.9. Finally, at 500KSIM1 the core tended to unravel with significant distortions in the overall native geometry (Fig 8) followed by the unfolding of both the helices to loops.

Average *Disnet* values were in agreement for all simulation sets from 310 to 500 K (Figure D in S1 File, Table D in S1 File) with some deviation observed for simulations 400KSIM5 and 450KSIM4. In addition, cross-correlation between Q and *Disnet* for the multiple simulations at all temperatures (SIM1-SIM5) was found to be in good agreement (Table E in S1 File).

For the native simulation at 310KSIM1 (which serves as the baseline) the values for <persf> and <dlf> (averaged over all the epochs) were 0.14 (0.03) and 0.39 (0.01) respectively, which implies that for <persf>, on an average about 86% of the contacts prevalent in the core of LdCyP ‘persisted’ throughout the course of the simulation at 310KSIM1. At 500 KSIM1, the same measure dropped to about 25%. Both <persf> and <dlf> exhibited regular and graduated increase as a function of temperature, increasing by about 0.1–0.2 for each incremental rise in temperature (Table 4).

**Table 4:** Dlf and persf values averaged over the entire simulation block (between 2–50 ns) for the hydrophobic core of LdCyp at different simulation temperatures (310KSIM1, 400KSIM1, 450KSIM1 and 500KSIM1). The standard deviations are given in parentheses.

Temperature (K)	<dlf>	<Persf>
310KSIM1	0.39(0.01)	0.14(0.03)
400KSIM1	0.49(0.02)	0.31(0.08)
450KSIM1	0.65(0.05)	0.49(0.16)
500KSIM1	0.76(0.07)	0.75(0.18)

**Fig 9:**
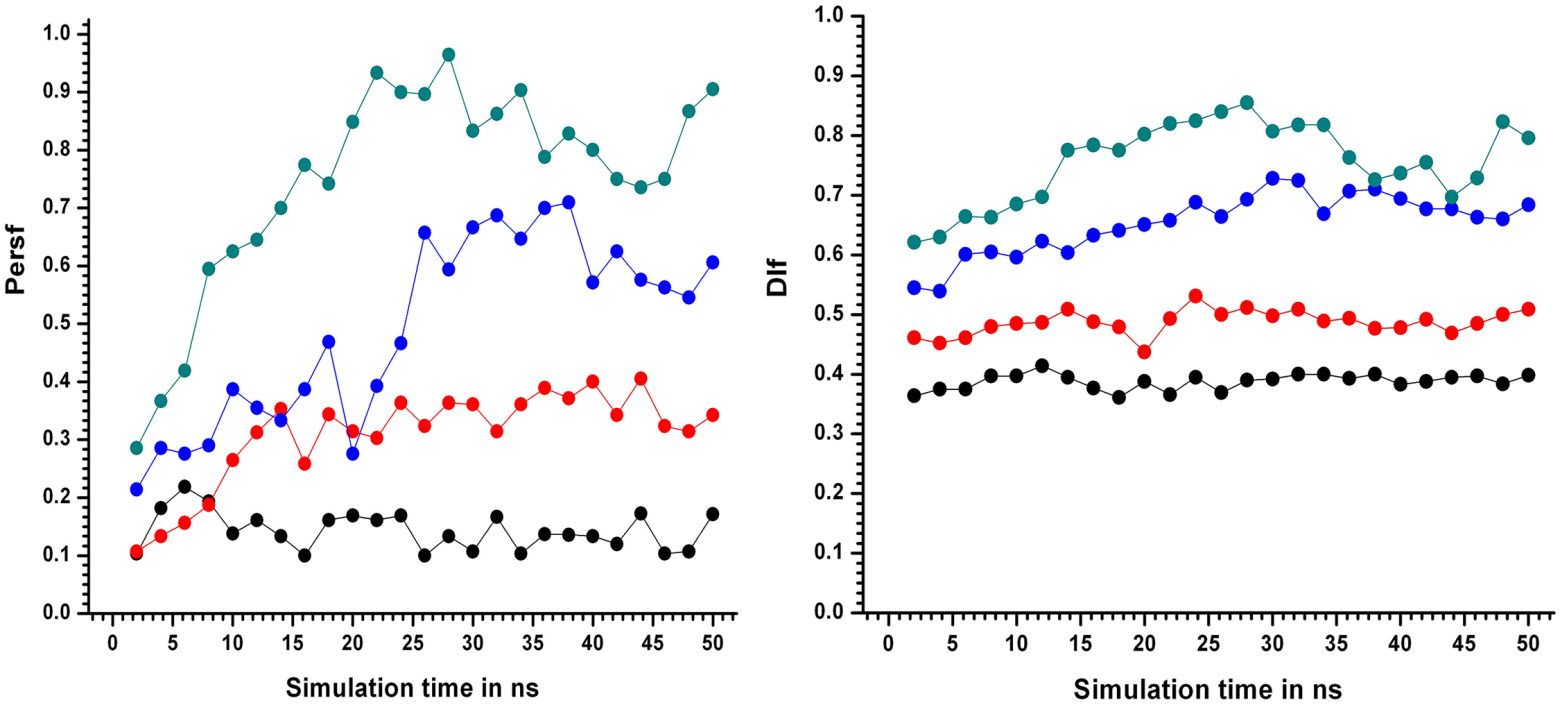
Persf values at different simulation temperatures. Persf values for 2ns epochs indicated by filled circles (considering only the hydrophobic core of LdCyp) as a function of simulation time for simulation sets310KSIM1 “black solid line”, 400KSIM1 “red solid line”, 450KSIM1 “blue solid line”, 500KSIM1 “green solid line”.b) Dlf values for 2ns epochs indicated by filled circles plotted as a function of simulation time. The color scheme is the same as above. At 450 K the sudden surge in persf values post 24 ns was indicative of the transition (TS) state.

Significant differences were observed in the time course of these two measures. Specifically, around 25 ns at 450KSIM1, a sharp surge in persf from about 0.30 to 0.60 was observed (Fig 9), preceded by a relatively unstable region beginning from 13 ns.

A similar rise was observed in the other simulations at 450K (SIM2-5), though not beginning from the same time point (Figure E in S1 File). In the same temporal region dlf exhibited a more gradual increase (from 0.60 to 0.75)(Fig 9) probably due to smoothening of the function by averaging over all pairs of snapshots in an epoch (450KSIM1).

However, persf demonstrated lesser fluctuations in the other simulation trajectories (450KSIM2-5) compared to dlf (Figure F in S1 File, Table F in S1 File). At 500KSIM1, both persf and dlf values had increased to about 0.90 indicating almost complete unraveling of native packing in the core. The most notable feature in both measures was their highly sensitive signaling of the gradual dynamic relaxation of packing constraints in the hydrophobic core of LdCyP, even at 400 K, indicated both by average and real time values. It will be recalled that such sensitivity was not prominently exhibited even by *Disnet* alone. Averages of the metrics Cα RMSD, persf and *Disnet* over all the 5 simulations demonstrated the separation of the Cα RMSD and *Disnet* curves at 450 K from 310, 400 (which were by and large coincident), over and above their standard deviations (Figure G in S1 File). However, the same curves occasionally approached each other within their respective standard deviations for persf (450 versus 400, 310 K). The average values at 500 K were well separated from their corresponding values at other temperatures (exceeding their individual fluctuations) for all the metrics.

**Fig 10:**
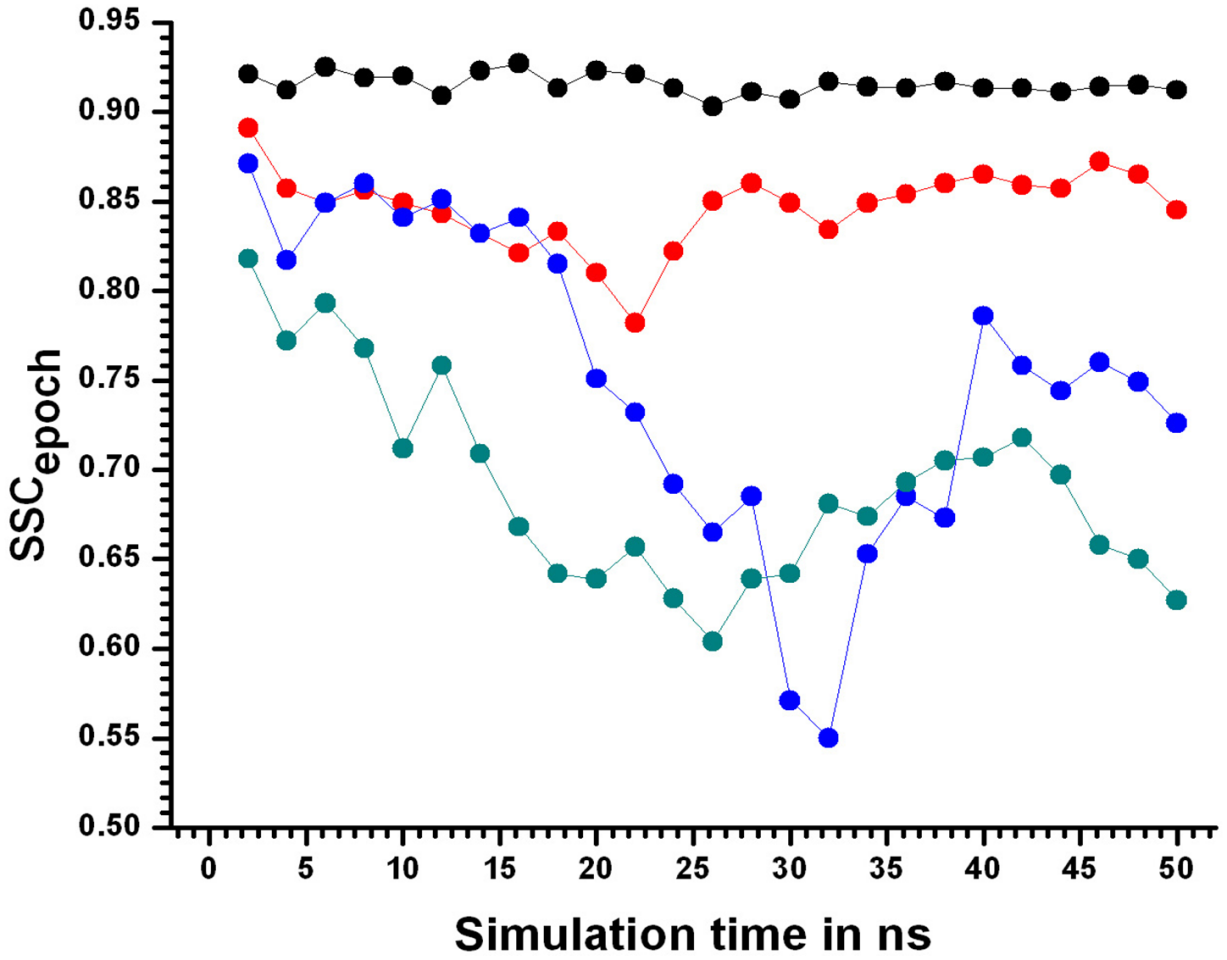
Fraction of secondary structural content. Fractional secondary content SSC_epoch_ values for every 2 ns epochs represented by filled circles joined by solid lines plotted as a function of simulation time. The simulations at different temperatures are indicated for 310KSIM1 “black filled circles joined by black solid lines”, 400KSIM1 “red filled circles joined by red solid lines”, 450KSIM1 “blue filled circles joined by blue solid lines” and 500KSIM1 “green filled circles joined by green solid lines”.

Analysis of the fractional secondary structural content of LdCyp (Materials & Methods) which is the fraction of residues constituting helices/strands in a snapshot (as found in the native crystal structure) and averaged over a 2ns epoch (SSC_epoch_), confirmed stable secondary structural elements in the native 310KSIM1 simulation block. At 400KSIM1, helix H2 exhibited a tendency to unwind upon dissociation from the hydrophobic core (for more details see section on Contacts given below), reflected in a 10% drop in SSC_epoch_. At 20ns (450KSIM1) there was a sharp decline in SSC_epoch_ (Fig 10) falling to a minimum value of 0.55 (at 32 ns) which indicated pronounced disruption for both helices and the barrel.

Even though SSC_epoch_ subsequently recovered somewhat to about 0.75 at 40 ns, post 20 ns all secondary structural features appeared to be irreversibly destabilized, culminating in relatively labile secondary structural features at 500KSIM1.

### Temporal patterns in the dissolution of native links

Next, the time evolution of the individual links in the network sustaining the hydrophobic core of the molecule was studied. As has been mentioned previously, the links of the surface contact network (including only core residues) were parsed into three subsets namely:

S1: links between residues located on ß-strands
S2: links between helix H1 and the remaining residues of the core and
S3: links between helix H2 and the remaining core residues.

All links were analyzed in terms of ‘persistence’ (Materials and Methods) which in effect is the number of times the link was observed in the snapshots spanning the epoch, divided by the total number of snapshots in the same interval (200). That is a persistence of 1.00 implies that the specific link was observed in all the snapshots of an epoch.

Only three links were observed in set S3, of which one (164 ILE-179 VAL) had a comparatively lower persistence of 0.50, even at 310 K (Table 5).

**Table 5:** Persistence of contacts averaged over the entire simulation run time, between residues within the core, divided into subsets of S1 (strand—strand or strand-loop contact), S2 (Strand-Helix1) and S3 (Strand-Helix2) at simulation temperatures 310, 400, 450 and 500 K (SIM1). The standard deviations are given in parentheses. The abbreviations in bracket represent: L-Loop, S-strand, H1-Helix1 and H2-Helix2. The drop in surface complementarity correlates well with sequential disruption of links in the unfolding process.

	SET S1		SET S2		SET S3	
Simulations	Core-Core	Persistence	Helix H1-Core	Persistence	Helix H2-Core	Persistence
310KSIM1	120LEU(S)-151PHE(S)	0.98(0.01)	59 PHE(H1)-151PHE(S)	0.98(0.08)	85 PHE(S)-164PHE(H2)	0.98(0.01)
	29VAL(S)-47LEU(S)	0.96(0.03)	62 LEU(H1)-71 TYR(L)	0.98(0.01)	85 ILE(S)-161VAL(H2)	0.78(0.05)
	122 MET(S)-151PHE(S)	0.94(0.03)	29VAL(S) -63CYS(H1)	0.96(0.02)	164 ILE(H2)-179VAL(S)	0.50(0.07)
	31PHE (S)-181ILE(S)	0.92(0.04)	55THR(H1)-151PHE(S)	0.96(0.03)		
	76 PHE(L)-85 ILE(S)	0.84(0.06)	59 PHE (H1)-71TYR(L)	0.96(0.02)		
	31PHE(S)-45ILE(S)	0.71(0.09)	55THR(H1)-122MET(S)	0.93(0.03)		
	76 PHE(L)-181 ILE(S)	0.68(0.18)	47LEU(S)- 59PHE(H1)	0.67(0.14)		
	71 TYR(L) -134 PHE(S)	0.64(0.10)	59PHE(H1)-134PHE(S)	0.59(0.09)		
	31PHE (S)-134PHE(S)	0.46(0.15)	31PHE(S)-59PHE(H1)	0.26(0.20)		
	31PHE (S)-71TYR(S)	0.30(0.31)				
	33VAL(S -179VAL(S)	0.18(0.16)				
400KSIM1	120 LEU(S)-151 PHE(S)	0.94(0.03)	62 LEU(H1)-71 TYR(L)	0.99(0.01)	85 ILE(S)-164ILE(H2)	0.49(0.19)
	31 PHE(S)-181 ILE(S)	0.88(0.03)	55 THR(H1)-151PHE(S)	0.98(0.02)	164 ILE(H2)-179VAL(S)	0.47(0.09)
	29 VAL(S)- 47 LEU(S)	0.82(0.08)	29 VAL(S)-63 CYS(H1)	0.94(0.03)	85 ILE(S)-161VAL(H2)	0.39(0.12)
	76 PHE(L)- 85 ILE(S)	0.78(0.08)	59 PHE(H1)-151PHE(S)	0.93(0.05)		
	76 PHE(L)-181 ILE(S)	0.77(0.08)	59 PHE(H1)-71 TYR(L)	0.87(0.07)		
	71 TYR(L)-134 PHE(S)	0.77(0.01)	59 PHE(H1)-134PHE(S)	0.78(0.07)		
	122 MET(S)-151PHE(S)	0.70(0.10)	47 LEU(S)-59 PHE(H1)	0.72(0.08)		
	31 PHE(S)-71 TYR(S)	0.65(0.18)	55 THR(H1)-122MET(S)	0.56(0.23)		
	31 PHE(S)- 45 ILE(S)	0.55(0.06)	31 PHE(S)-59PHE(H1)	0.49(0.11)		
	31 PHE(S)-134 PHE(S)	0.54(0.10)				
	33 VAL(S) -179 VAL(S)	0.47(0.10)				
450KSIM1	31PHE (S)- 181ILE(S)	0.87(0.06)	47LEU(S)-59PHE(H1)	0.55(0.22)	164 ILE(H2)- 179VAL(S)	0.46(0.09)
	71 TYR(L)—134PHE(S)	0.68(0.20)	62 LEU(H1)-71 TYR(L)	0.54(0.28)	85ILE(S) -164ILE(H2)	0.45(0.10)
	122 MET(S)-151PHE(S)	0.59(0.14)	59 PHE (H1)-71TYR(L)	0.51(0.27)	85 ILE(S) -161VAL(H2)	0.37(0.09)
	76 PHE(L)—85 ILE(S)	0.57(0.11)	29VAL(S)—63CYS(H1)	0.49(0.34)		
	31PHE(S) -45ILE(S)	0.56(0.08)	59 PHE(H1)-151PHE(S)	0.48(0.40)		
	120 LEU(S) -151PHE(S)	0.53(0.26)	55THR(H1)-151PHE(S)	0.35(0.37)		
	76 PHE(L)- 181 ILE(S)	0.51(0.07)	59PHE(H1)-134PHE(S)	0.26(0.27)		
	31PHE (S)- 71TYR(S)	0.47(0.17)	55THR(H1)- 122MET(S)	0.15(0.24)		
	29VAL(S)- 47LEU(S)	0.41(0.25)	31PHE(S)-59PHE(H1)	0.10(0.13)		
	31PHE (S)- 134PHE(S)	0.20(0.14)				
	33VAL(S)—179VAL(S)	0.17(0.12)				
500KSIM1	31PHE (S)-181ILE(S)	0.60(0.14)	55THR(H1)—151PHE(S)	0.55(0.25)	164 ILE(H2)- 179VAL(S)	0.07(0.10)
	76 PHE(L)-85 ILE(S)	0.48(0.13)	47LEU(S)-59PHE(H1)	0.50(0.17)	85 ILE(S)-161VAL(H2)	0.06(0.10)
	29VAL(S)- 47LEU(S)	0.41(0.20)	29VAL(S)-63CYS(H1)	0.39(0.31)	85ILE(S)-164ILE(H2)	0.02(0.05)
	33VAL(S)-179VAL(S)	0.40(0.09)	59PHE(H1)-134PHE(S)	0.31(0.24)		
	120 LEU(S) -151PHE(S)	0.35(0.23)	62 LEU(H1)-71TYR(L)	0.31(0.26)		
	76 PHE(L)-181ILE(S)	0.29(0.16)	59 PHE(H1)-151PHE(S)	0.26(0.32)		
	31PHE(S)-45ILE(S)	0.27(0.18)	55THR(H1)-122MET(S)	0.16(0.18)		
	122 MET(S)-151PHE(S)	0.25(0.19)	59 PHE (H1)-71TYR(L)	0.08(0.19)		
	71 TYR(L)-134PHE(S)	0.22(0.27)	31PHE(S)-59PHE(H1)	0.07(0.09)		
	31PHE (S)-134PHE(S)	0.17(0.18)				
	31PHE (S)-71TYR(S)	0.14(0.16)				

A decline of about 40–50% in persistence for all the links in S3 at 400 K unambiguously indicated the early dislocation of helix H2 from the barrel.

In contrast, 9 links involving helix H1 (set S2) maintained persistence levels (at 400 K) similar to the native simulation (at 310), implying conservation of the native geometry with regard to the barrel and helix H1. A similar situation was observed for the 11 links in the set S1, denoting a fairly compact hydrophobic core, closely resembling the native. However for both sets (S1 and S2) the links displayed a range of persistence values, the most stable links being 31 PHE-181 ILE, 120 LEU-151 PHE, 29 VAL- 47 LEU, 122 MET-151 PHE and 59 PHE-151 PHE, 62 LEU-71 TYR, 29 VAL-63 CYS, 55 THR-151 PHE, 59 PHE-71 TYR in sets S1, S2 respectively.

At 450 K, there was an overall decline of about 40–50% in the persistence of most links in S1, S2 indicating a profound disruption in core packing concomitant to the dislocation of the helix H1 from the barrel. In S1 only links 31 PHE-181 ILE, 71 TYR-134 PHE, were able to maintain persistence levels comparable to the native simulation. In the vicinity of the transition state (around 25 ns at 450 K) erstwhile high persistence strategic links (29 VAL—47 LEU, 76 PHE-85 LEU, 120 LEU-151 PHE and 122 MET-151 PHE) in S1 and (29 VAL-63 CYS, 55 THR-151 PHE, 59 PHE-71 TYR, 59 PHE-151 PHE, 62 LEU-71 TYR) in S2 declined rather sharply possibly suggesting that the onset of the transition state could be signaled by the collapse of the strategic high persistent links that are integral in maintaining both stability of the core and the association of helix H1 with the barrel.

At 500 K helix H2 dissociated completely from the main body of the protein and despite further decline in persistence in S1, S2 yet a few selected residual links (31 PHE-181 ILE, 76 PHE– 85 ILE, 55 THR-151 PHE and 47 LEU-59 PHE) still retained persistence values of about 0.50. Thus despite dissociation of the helices and dissolution of the core a few native links could still be found.

**Fig 11:**
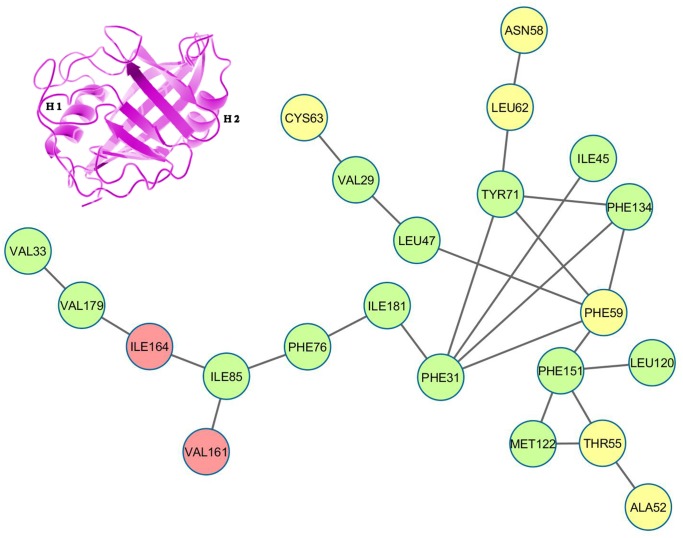
Persistence network diagram at 310 K. Persistence network diagram of an epoch from the simulation at 310 K (2ns) depicting the stability of all the topological regions L1 (composed of residues 33 VAL, 179 VAL, 164 ILE, 85 ILE, 76 PHE, 181 ILE, 161 VAL), L2 (composed of residues 63 CYS, 29 VAL), L3 (composed of residues 58 ASN, 62 LEU), TC(composed of residues 151 PHE, 122 MET, 55 THR) and QC (composed of residues 31 PHE, 71 TYR, 134 PHE, 59 PHE) with residues (nodes) located on helix H1 colored in yellow, helix H2: red and strand residues: green. A ribbon diagram of a representative snapshot from within the epoch is depicted on the right hand corner.

**Fig 12:**
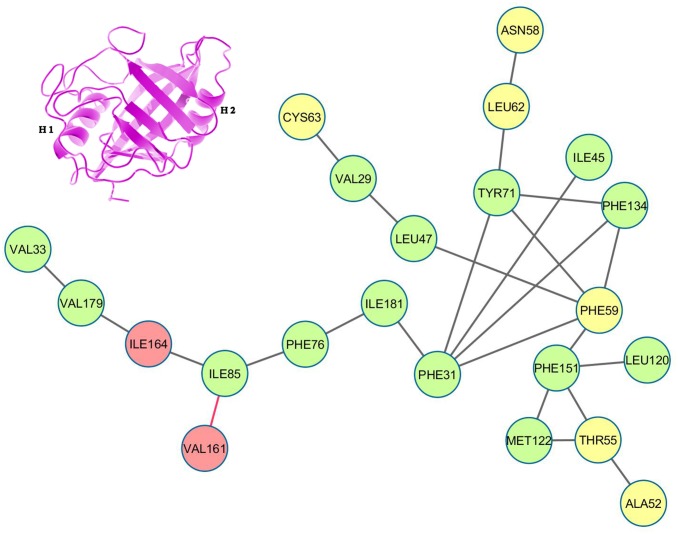
Persistence network diagram at 400 K. Persistence network diagram of an epoch from the simulations at 400 K (4 ns) showing some signs of disruption in L1 (the absence of a pre-existing link is marked in “red solid line”)with residues (nodes) located on helix H1 colored in yellow, helix H2: red and strand residues: green. A ribbon diagram of a representative snapshot from within the epoch is depicted on the right hand corner. The color scheme followed is same as above. Networks at 400 K bear a close resemblance with the native network.

**Fig 13:**
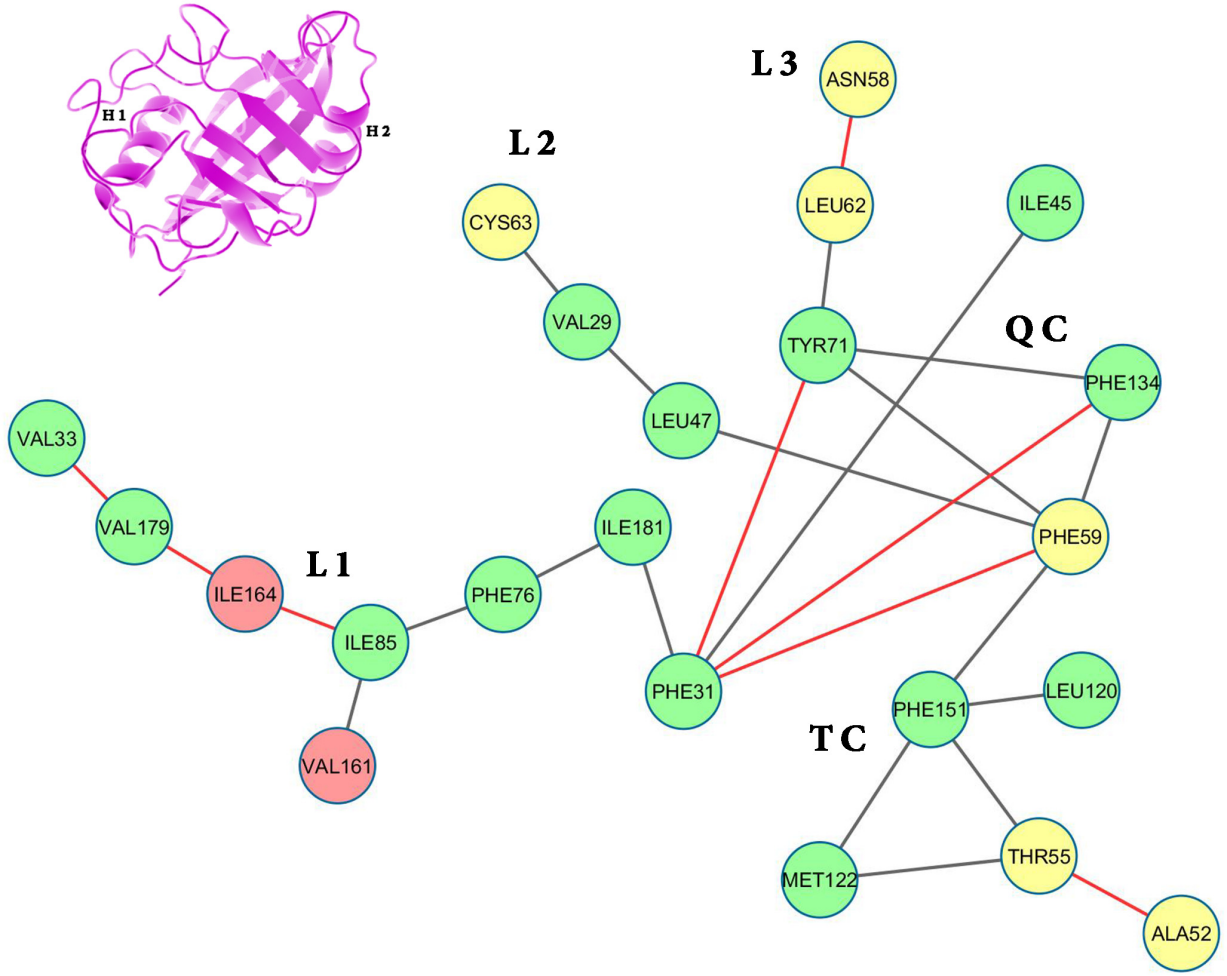
Persistence network diagram in the early stages of 450 K simulation. Persistence network diagram of the epoch at 4 ns (450 K SIM1) showing signs of instability in L1 and QC (the breakage of original links are shown in “red solid line”)with residues (nodes) located on helix H1 colored in yellow, helix H2: red and strand residues: green. A ribbon diagram of a representative snapshot from within the epoch is depicted on the right hand corner. The color scheme followed is same as above.

**Fig 14:**
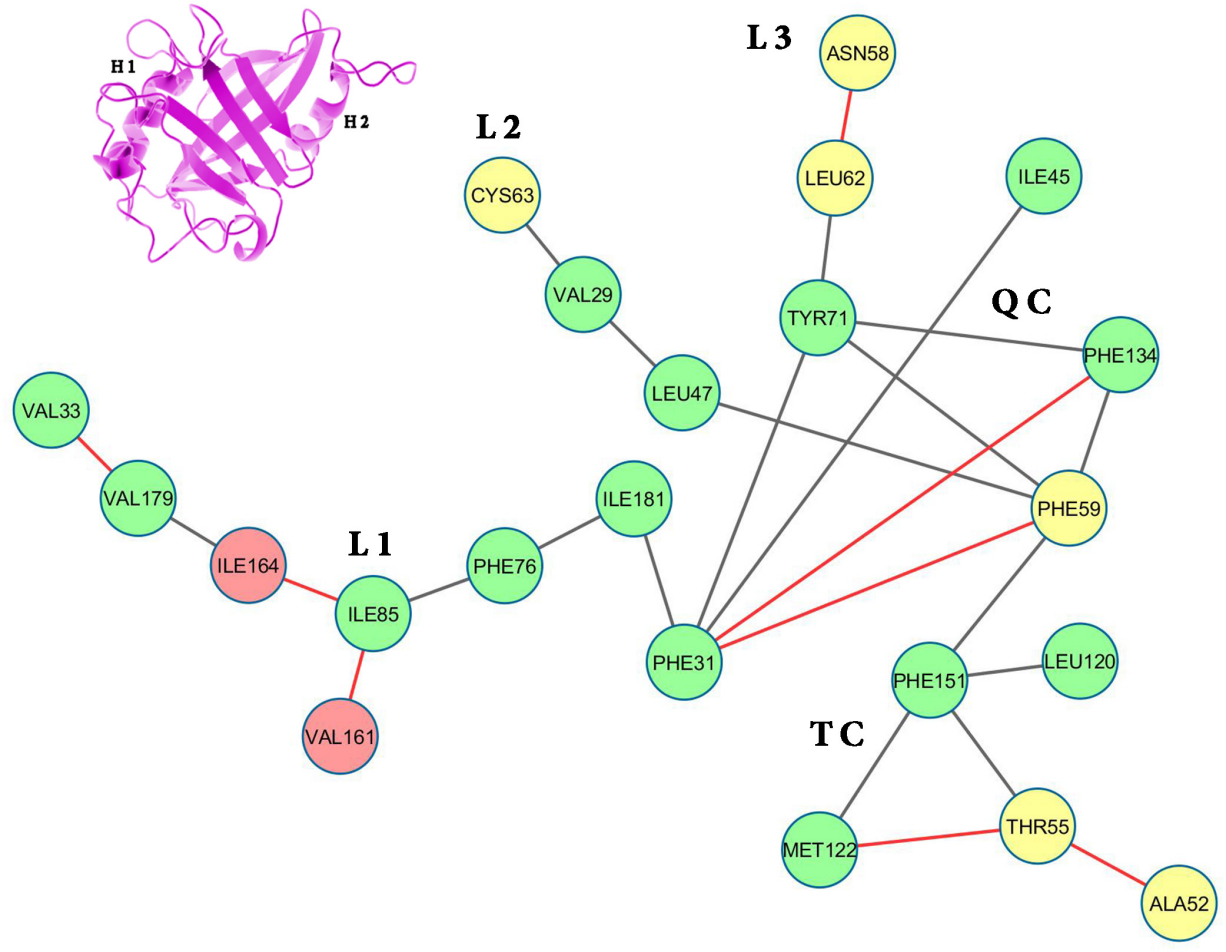
Persistence network diagram at 12 ns of 450 K simulation. Persistence network diagram of the epoch at 12 ns (450 K) with residues (nodes) located on helix H1 colored in yellow, helix H2: red and strand residues: green. A ribbon diagram of a representative snapshot from within the epoch is depicted on the right hand corner. The color scheme followed is same as above.

**Fig 15:**
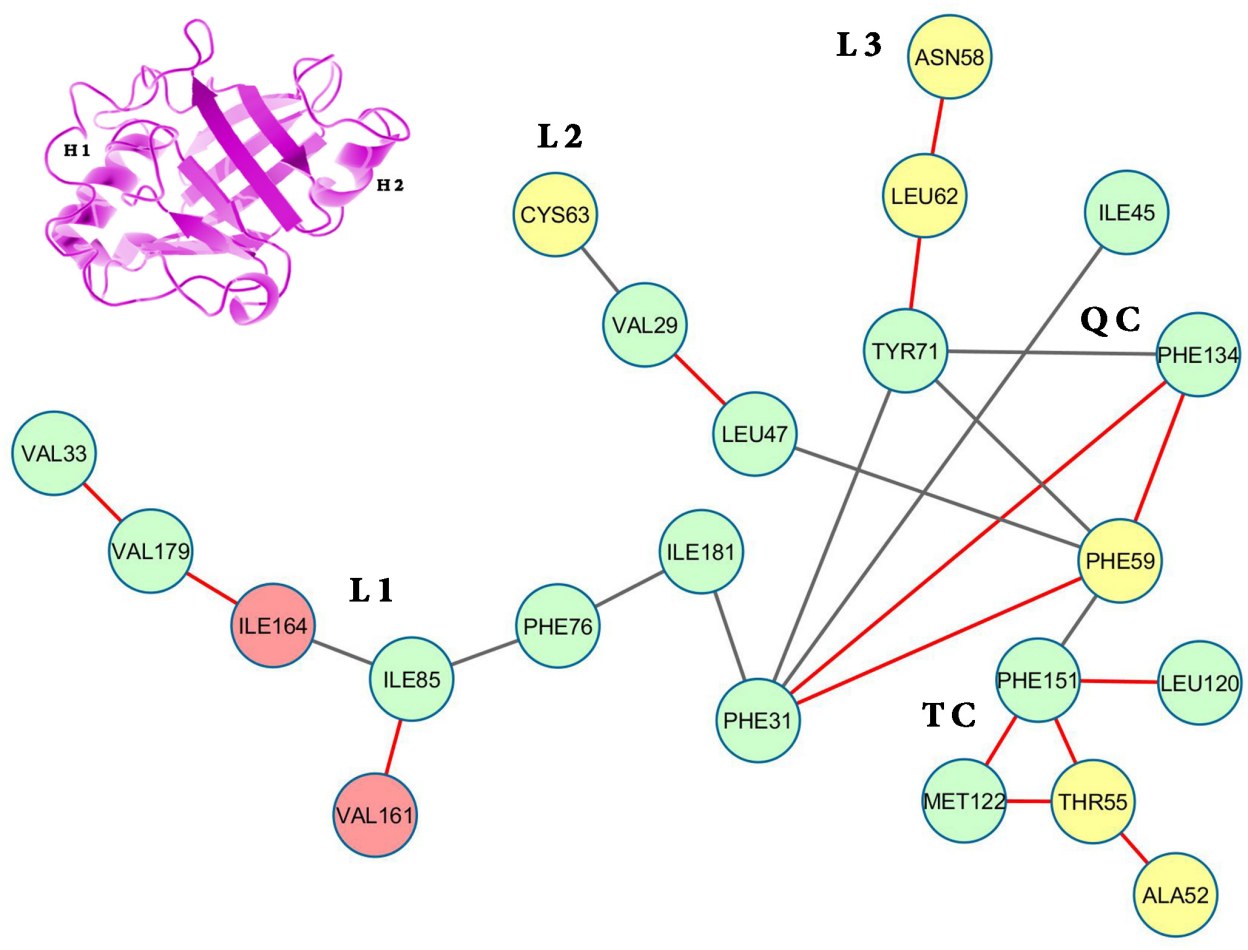
Persistence network diagram within the transition state at 450 K simulation. ‘Persistence network’ diagram of the epoch at 26 ns (450 K) within the main transition state of unfolding showing pronounced disruption in links in L1, QC and TC. The color scheme followed is same as above.

**Fig 16:**
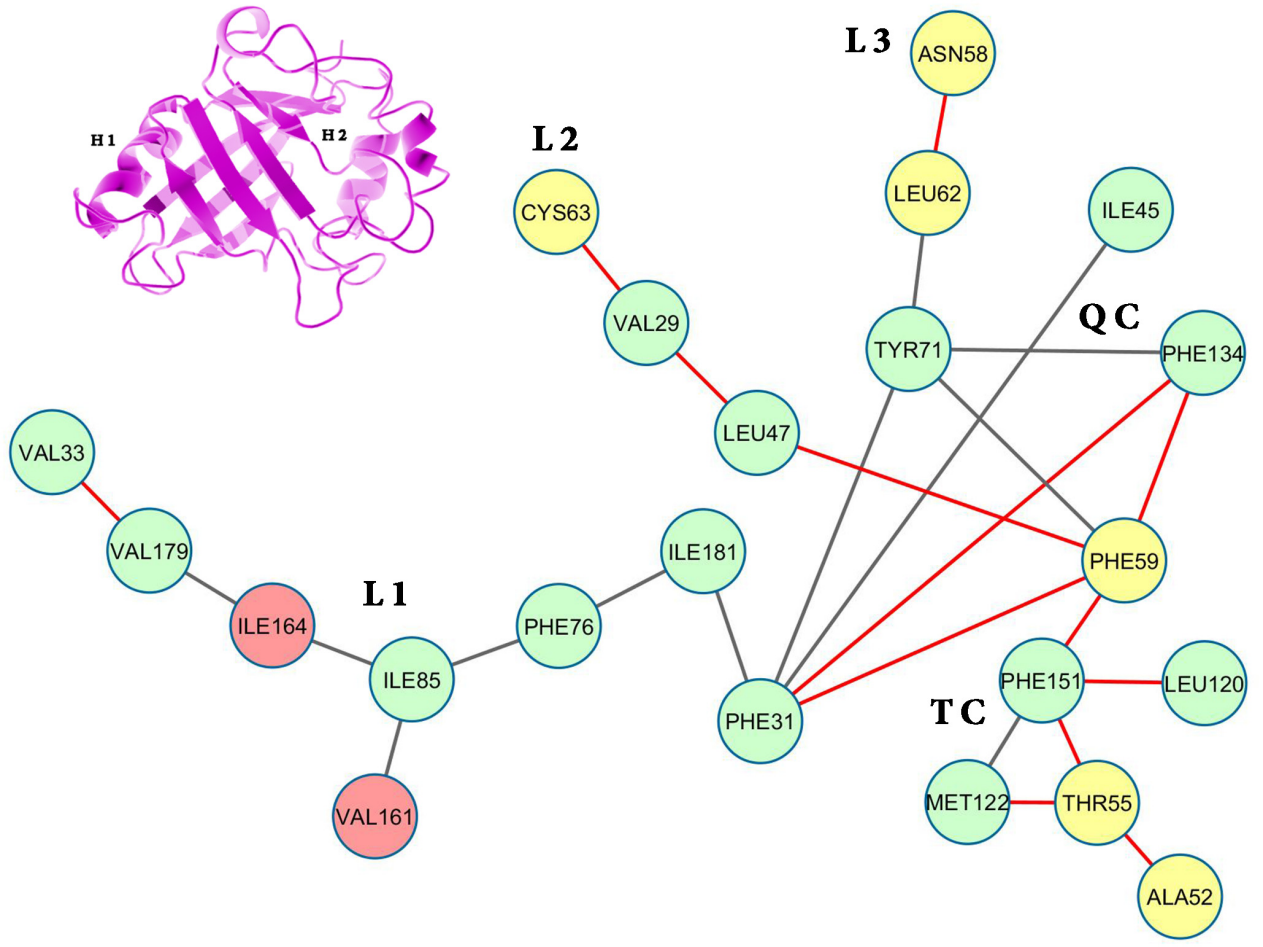
Persistence network diagram at the largely unfolded states at 44ns, 450 K. Persistence network diagram of 44 ns epoch at 450 K showing disruption in L1, L2, QC and TC with the breakage of link 29 VAL– 47 LEU. The color scheme followed is same as above.

Depiction of ‘persistence’ networks linking residues when their surface contact was observed in more than 40% of the snapshots in an epoch (‘persistence matrix’ being the corresponding adjacency matrix for such networks; **Material & Methods**) gave additional insights into the sequence of transitions in the unfolding process. Figs 11–16 depict the persistence networks for representative epochs during the course of the unfolding.

The most prominent topological features of the network in the native simulation and at 400 K was the relative stability of a 4 (**QC**: 71 TYR, 134 PHE, 31 PHE, 59 PHE) and 3 (**TC**: 151 PHE, 122 MET, 55 THR) membered clique involving residues from the core and helix H1, in addition to three linear extensions (**L1**: 181 ILE, 76 PHE, 85 ILE, 161 VAL, 164 ILE, 179 VAL, 33 VAL; **L2**: 47 LEU, 29 VAL, 63 CYS and **L3**: 62 LEU, 58 ASN) emanating from the quadruplet clique QC. These network structures dominating the topological landscape denoted complete integrity of the network involving almost all the residues of the core (Figs 11 and 12) and extensive ‘inter-locking’ of helical residues with those of the barrel.

**Fig 17:**
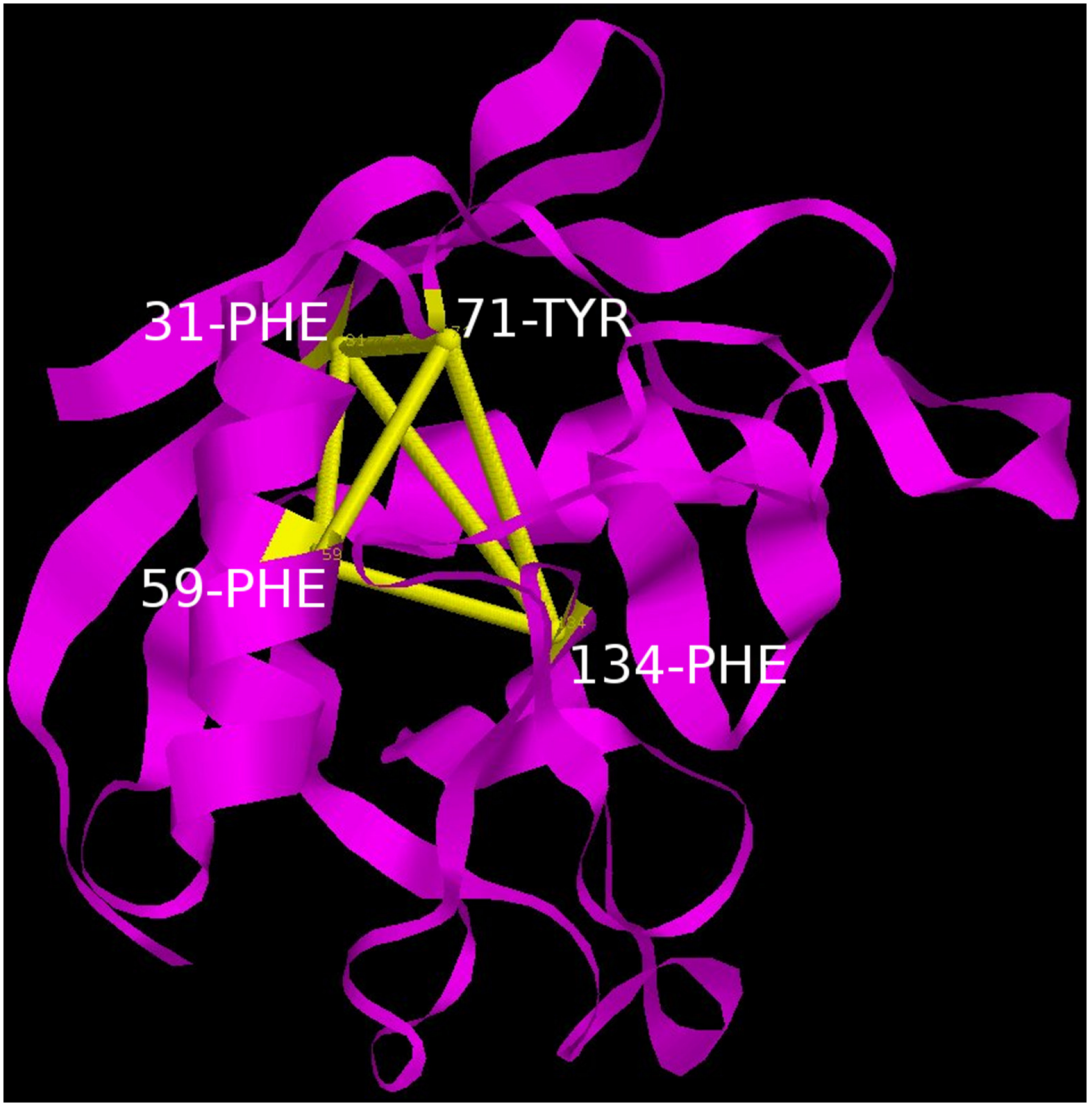
Four membered clique. A four membered clique consisting of residues 31 PHE, 59 PHE, 71 TYR and 134 PHE connecting the helix H1 with the ß-sheet.

**Fig 18:**
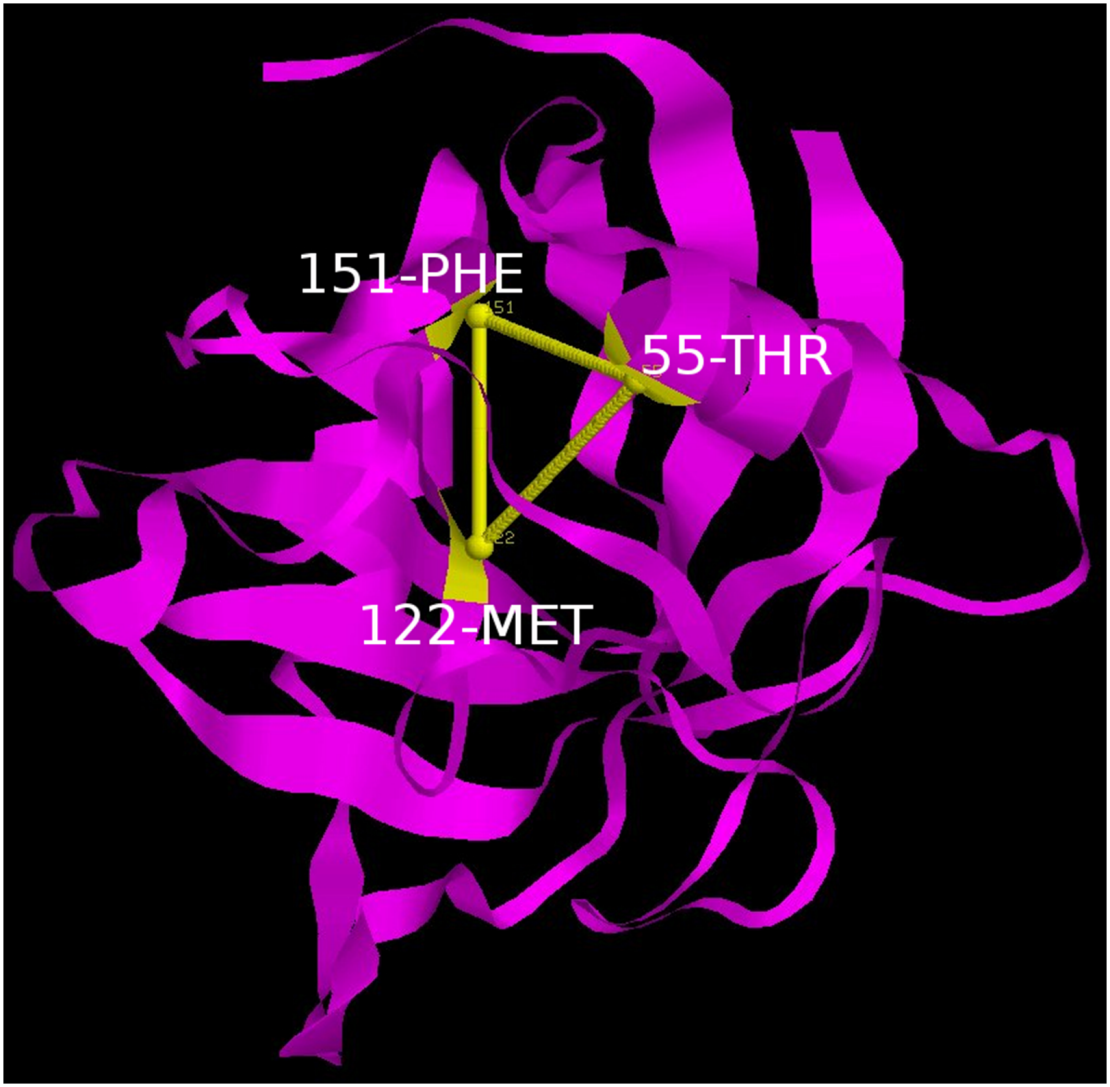
Three membered clique. A three membered clique consisting of residues 55 THR, 122 MET and 151 PHE connecting the ß-sheets.

Notably, 59 PHE and 55 THR contributed by helix H1 were constituents of both cliques QC and TC. Topology of the links involved in the cliques show that in a sense they constitute a scaffold weaving the disparate secondary structural elements into a composite molecular entity (Figs 17 and 18).

Broadly, the unfolding process could be divided into four phases: i) initiation with progressive acquisition of DMG like characteristics (2–8 ns), ii) WMG like phase (8–22 ns) iii) transition state (22–30 ns) and iv) completely unfolded 20 to 50 ns at 500 K. These transitions especially entry into DMG, WMG states subsequent to initiation of unfolding were demarcated based on the extent of water interactions with the core which has been discussed in detail in the next section, and the values adopted by the relevant network parameters in these phases. Here, the sequence of changes in network structure during each phase of unfolding (DMG, WMG, TS etc.) has been elaborated.

**Initiation/DMG**—the initiation of unfolding at 450 K in the first few ns involved i) instabilities appearing in the links 31 PHE– 71 TYR, 31 PHE– 134 PHE, 31 PHE– 59 PHE in QC and ii) fluctuations in L1 due to the weakening of links 85 ILE– 161 VAL, 85 ILE– 164 ILE or 164 ILE– 179 VAL (Fig 13; **See** Table 5 **for reduction in persistence values indicative of altered surface complementarity of the specific links mentioned above**). Thus the very first events in unfolding involved the strategic nodes 31 PHE and 85 ILE. Progressive weakening of the above mentioned links continuously pushed the molecule into DMG like states.

**WMG Phase**—entry into WMG states past 10 ns was signaled by the pronounced disruption of the above links causing the collapse of QC into either two independent triplets or a simple assortment of links and dislocation of helix H2 due to heightened fluctuations in L1 (Fig 14). In addition, breakage of link 55 THR– 122 MET in TC definitely denoted the altered arrangement of helix H1 with respect to the core. The increased conformational flexibility of these residues was also corroborated in their respective RMSF values (Figure B in S1 File).

**TS Region**—the next major events occurred around 22–30 ns upon entry into the transition state (TS) region, namely iii) severance of link 29 VAL– 47 LEU in region L2 iv) breakage of 55 THR– 151 PHE in erstwhile TC, v) disruption of L3 due to breakup of links 71 TYR– 62 LEU, 62 LEU– 58 ASN and vi) further dissolution of erstwhile QC by breakage of link 59 PHE– 134 PHE (Fig 15).

**Largely Unfolded**—past TS the disintegration of the network assumed an increasingly cooperative character with almost simultaneous rapid collapse of all topological regions of the network (b) with the connection between L2 and QC being severed by the permanent disruption of link 47 LEU- 59 PHE. In summary, the unfolding process involves the sequential, gradual and localized disruption of the cliques and extended regions before TS whereas subsequent to entry into TS, the dissolution of all topological regions (specifically L2, L3, QC, TC) is rapid and cooperative.

Upon overlaying all the five simulations (SIM1—SIM5: Figure H—N in S1 File) the consensual sequences of events were as follows:

1. At 6 ns (450 K) QC is reduced to two triplet cliques (71 TYR-134 PHE- 59 PHE and 31 PHE-134 PHE- 71 TYR) due to the disruption of link 31 PHE– 59 PHE (Figure H in S1 File).
2. At 10 ns disruption in L1 due to breakage of links 33 VAL– 179 VAL, 179 VAL– 164 ILE and 164 ILE– 85 ILE (Figure I in S1 File).
3. At 32 ns one of the two cliques formed due to the disintegration of QC collapses due to the severance of link 31 PHE– 134 PHE (Figure J in S1 File) whereas the other triplet clique dissociates from L2 at 38 ns due to breakage of 47 LEU– 59 PHE (Figure K in S1 File).
4. At 4–8 ns 500K, TC dissolves due to disruption of all the constituent links (Figure L in S1 File). Further 8 ns onwards breakage of 59 PHE– 71 TYR disrupts the remaining triplet cliques left over from QC (Figure M in S1 File)
5. Finally at 10 ns 500 K one of the last remaining links of erstwhile QC, 31 PHE– 71 TYR dissolves (Figure N in S1 File).

SIM1 (450, 500 K) and the overlay of all 5 simulations (SIM1-SIM5) generally follow the same sequence of events even though the occurrence of specific events occur at different time points due to the expected non—overlap of the 5 simulations. These events can be summarized as follows

1. Dislocation of the hubs (31 PHE, 59 PHE) in QC followed by disintegration of L1 releasing helix H2 from the core.
2. Further relaxation of packing in erstwhile QC and instability in TC resulting in helix H1 adopting non-native geometries with respect to the core.
3. Cooperative dissolution of L2, L3, erstwhile QC and TC at the transition state region and disintegration of the remaining links thereafter.

### Solvation of the hydrophobic core and identification of MG like states

**Fig 19:**
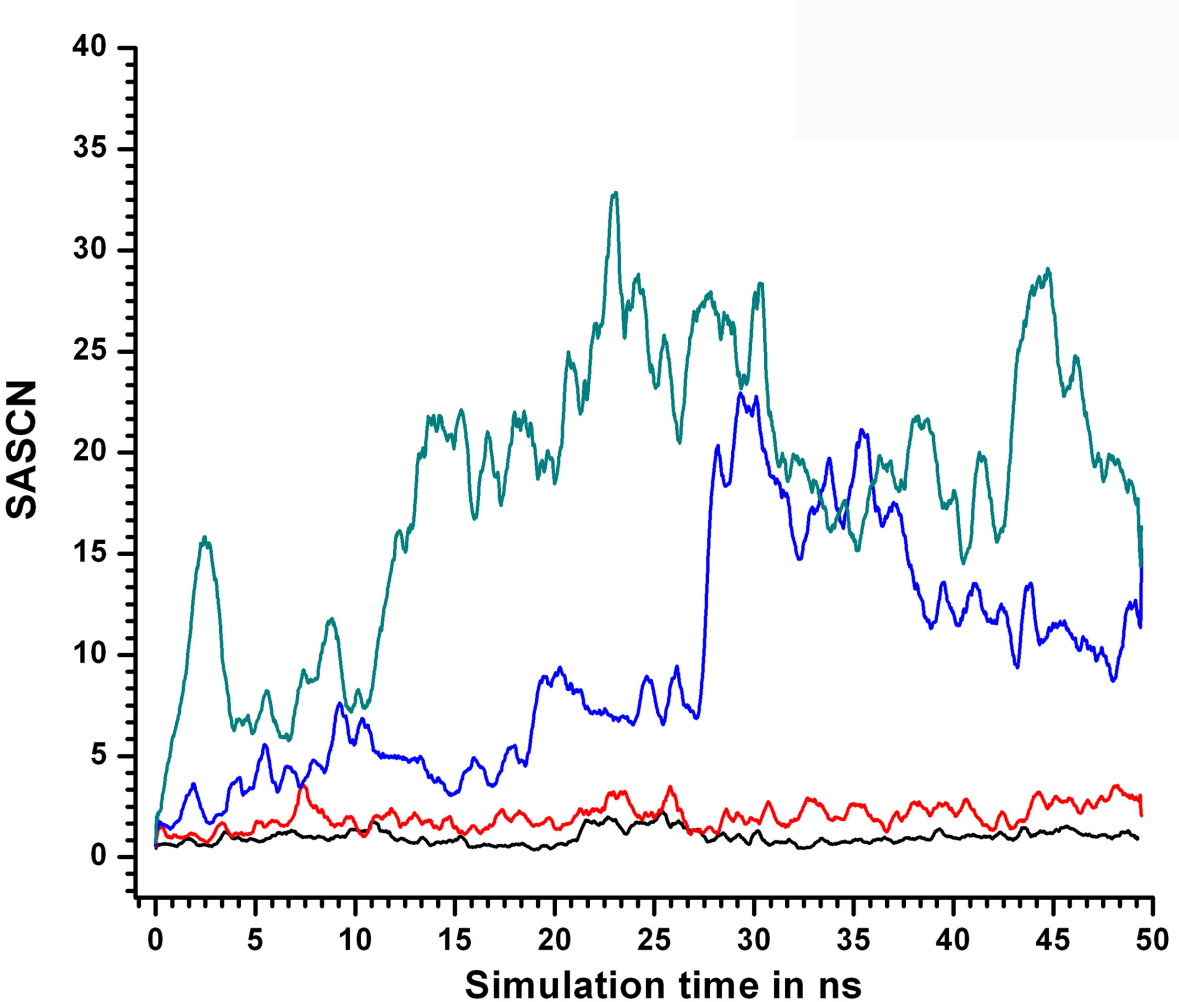
Variation in SASCN at different simulation temperatures. SASCN values plotted as a function of simulation time for different simulation temperatures; 310KSIM1 “black solid line”, 400KSIM1 “red solid line”, 450KSIM1 “blue solid line” and 500KSIM1 “green solid line”. The abrupt increase in SASCN at TS is evident post 24 ns in 450 K.

The measure SASCN was designed to estimate solvent access to the hydrophobic core and was defined as the ratio of the sum of solvent accessible areas of residues (considering side chain atoms alone) constituting the core to the average of the same sum computed from snapshots obtained from the native simulation at 310 K (Materials and Methods). Since the core is expected to be progressively exposed during the course of unfolding, SASCN should increase with rise in temperature. The sum of the solvent accessible areas of the core residues for the native crystal structure (2HAQ) was 7.81 Å^2^ while the average value of the same measure considering the snapshots of the native simulation was found to be 21.14 Å^2^ (the value used for normalization in SASCN). The average SASCN estimated from the snapshots at 310, 400, 450, 500 K (SIM1) were 1.0 (0.51), 2.02 (0.90), 9.50 (5.96) and 18.40 (7.44) respectively. Thus, at 400 K on an average, snapshots exhibited approximately twice the exposed solvent accessible area than observed in the native (at 310 K). By and large SASCN proved to be a highly sensitive indicator of the relative exposure of the core to solvent. The time evolution of SASCN was very similar for both the simulations at 310 and 400 K, with a radically altered pattern at 450 K. Relatively higher values of SASCN (between 4.0–5.0) were observed in the first 17 ns (at 450 K), which rose to about 9.0 for the next 10 ns. At the 27^th^ ns a steep increase to about 25.0 was observed as if signaling a phase transition (Fig 19), gradually declining to around 15.0 thereafter, by the 50^th^ ns.

In general, the behavior of SASCN was mutually consistent with the other metrics (*Disnet*, Q_L_ etc.). Snapshots from the unfolded state at 500 K, exhibited increased fluctuations in SASCN, which at its maximum value during the simulation exceeded 40.0.

Additionally, the number of water interactions with the core was also monitored with a distance cutoff of 5.0 Å and the average number of water interactions per epoch was calculated. There appeared to be progressive water penetration into the core (Figure O in S1 File) beginning with an average of approximately 11 waters accessing the core from 2–8 ns (the initiation/DMG region) to about 16 waters from 8–22 ns (WMG phase, 450KSIM1). The corresponding average value of solvent molecules penetrating the core for the overall native simulation at 310 K was 12.6. SASCN also exhibited a relative rise from 3.17 to 7.37 in the same two regions (native average at 310 K: 1.01). At TS (22–30 ns), core the values of the same parameters abruptly increased to 21 and 11.31 in water—core interactions and SASCN respectively (Table 6).

**Table 6:** Order parameters averaged over the relevant temporal regions to depict their transition during the process of unfolding. The metrics (*Disnet*, Q_L_, SASCN, Number of Waters and Cα RMSD) assume characteristically different values during the different phases of unfolding states (DMG, WMG and TS).

Order Parameters	Native states 310K (2–50ns)	DMG like states 450K (2–8ns)	WMG like states 450K (8–22ns)	TS States 450K (22–30ns)	Completely Unfolded states 500K (20–50ns)
*Disnet*	0.46(0.07)	0.54(0.08)	0.64(0.08)	0.76(0.07)	0.87(0.15)
Q_L_	0.73(0.08)	0.59(0.10)	0.49(0.11)	0.32(0.09)	0.22(0.14)
SASCN	1.01(0.51)	3.17(1.83)	7.37(2.35)	14.11(6.78)	18.72(7.87)
No. of Waters	12.6(2.9)	11.3(3.2)	16.4(5.1)	20.7(8.1)	41.0(8.4)
Cα RMSD	0.53(0.06)	0.87(0.25)	1.33(0.15)	1.37(0.18)	3.02(0.59)

The parameters clearly discriminate between the four states especially native, MG, TS and completely unfolded states. Between DMG and WMG states the distinction was more pronounced for SASCN and average water interactions than for *Disnet* and Q_L_. Again, for DMG and WMG states the standard deviation of the order parameters were in agreement with the native simulation between (2–50 ns; 310 K) rather than the ‘unfolded protein ‘ (20–50 ns; 500 K). The compactness of the core from 2–8 ns indicated by the average Cα RMSD (0.87 Å) appeared to resemble the native structure (0.53 Å averaged over 2–50 ns at 310 K) and rose to an average value of 1.33 Å between 8–22 ns (Table 6). The values of the network based metrics *Disnet*, Q_L_ in the two hypothetical MG regions (dry and wet) were 0.54, 0.59 and 0.64, 0.49 respectively. Thus, the transition between hypothetical DMG and WMG like states was rather gradual with probable overlap between them. In contrast the move from the MG states to the transition state region (22–30 ns) was abrupt with pronounced change in all the relevant network based metrics (Table 6). The network based metrics also reflected the properties of a transition state such as relative instability and rapid progression into non—native unfolded states thereafter.

To conveniently depict DMG, WMG and the transition state, the metrics (SASCN, Q_L_) of each snapshot was plotted (with color coding for the time coordinate). For the native simulation all the points were confined between 0.5 < Q_L_< 1.0 and 0 < SASCN < 5; being predominantly populated by structures closely resembling the native crystal structure. Accordingly this region of the plot was termed ‘Native-Like’ (Figure P in S1 File). As DMG structures should exhibit perturbed packing circumscribed by a solvent inaccessible core, a decline in Q_L_ could be expected while maintaining the same range in SASCN as observed in 310 K. Accordingly, the average in Q_L_, SASCN (obtained in the temporal period 2–8 ns, 450K) with a width of 1σ turned out to be 0.49 < Q_L_< 0.69 and 1.34 < SASCN < 5.0. This could probably be characteristic of DMG like states and tentatively the region spanning 0.5 < Q_L_ < 0.7 and 1 < SASCN < 5 was referred to as ‘DMG-Like’ (Figure P in S1 File). With further progress in unfolding, WMG structures could emerge accompanied by increased solvent exposure of the core concomitant to further relaxation of packing constraints. Thus, increase in the values of SASCN could definitely be expected, associated with further possible decline in Q_L_. Adopting the same procedure as above, the area bound by 0.4 < Q_L_ < 0.6 and 5 < SASCN < 10 of the plot was referred to as ‘WMG-Like’ (Figure P in S1 File). Similarly, the major transition state of unfolding (22–30 ns) was found to be located in the region bound by 0.2 < Q_L_ < 0.4 and 7.5 < SASCN < 20.0. Finally, at 500 K with the completion of unfolding there was an increased tendency of solvent exposure with SASCN exceeding 25 and Q_L_ also dropping below 0.10 (Figure P in S1 File) indicative of the protein sampling conformational states with hardly any resemblance to the native structure (‘Unfolded’).

**Fig 20:**
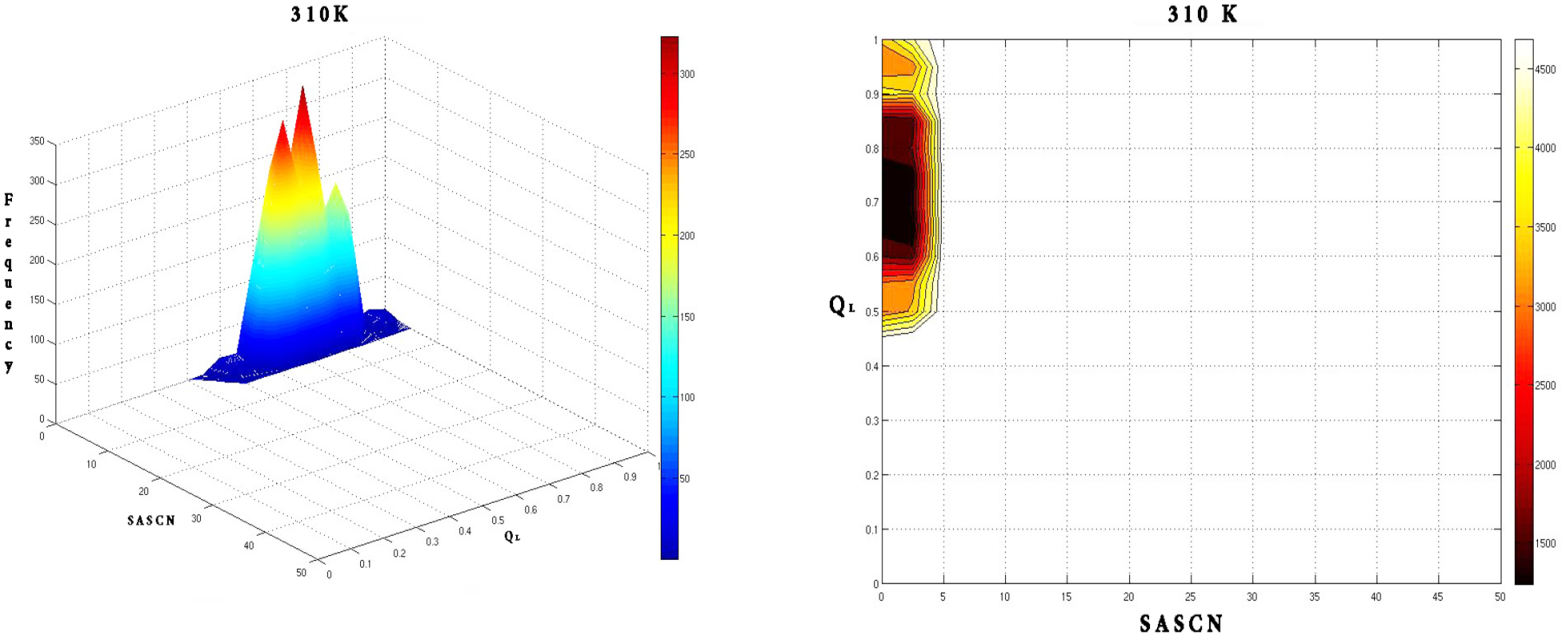
Frequency distribution and the corresponding free energy contour map at 310 K with reaction coordinates Q_L_ and SASCN. Frequency distribution at 310 K shows a dominant peak in the native region which is also reflected by a global minimum in energy around (Q_L_: 0.7; SASCN: 2.5) in the free energy contour map.

**Fig 21:**
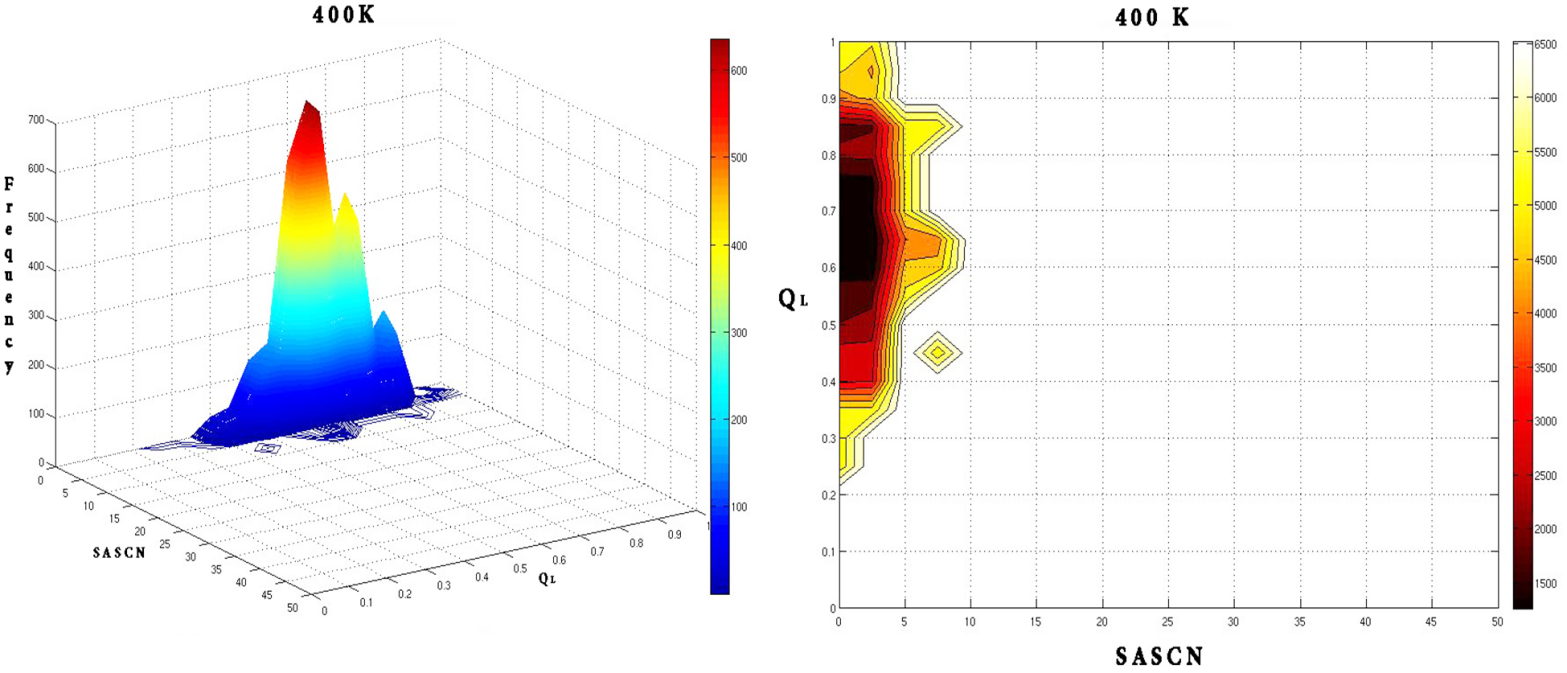
Frequency distribution and the corresponding free energy contour map at 400 K with reaction coordinates Q_L_ and SASCN. Frequency distribution and free energy contour map at 400 K are similar to that at 310 K with conservation of the dominant frequency peak in the native region and the global minimum in the energy landscape.

Calculation of the free energy landscapes for the Q_L_-SASCN plots at different temperatures exhibited prominent minima for the native simulation at 310 K centered about (Q_L_: 0.7; SASCN: 2.5) (Fig 20) which appeared to be conserved at 400 K (Fig 21).

**Fig 22:**
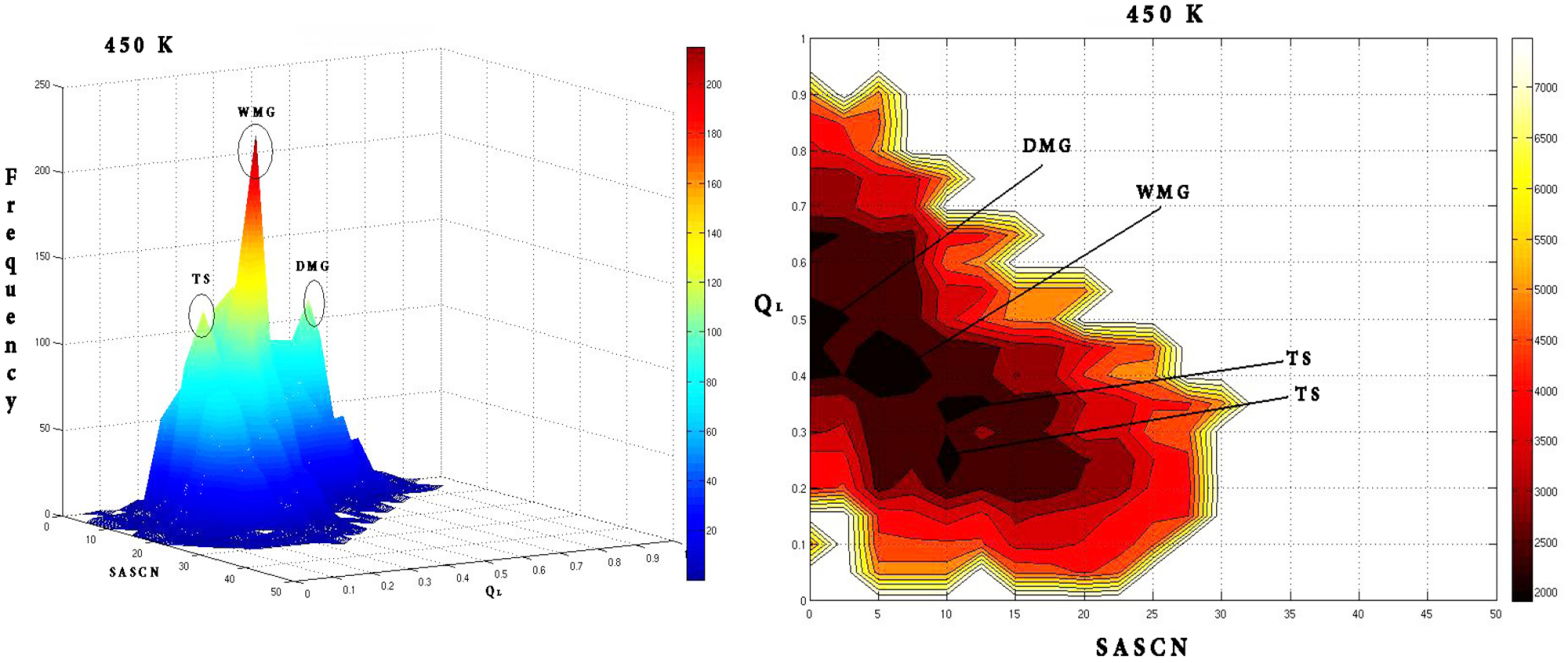
Frequency distribution and the corresponding free energy contour map at 450 K with reaction coordinates Q_L_ and SASCN. Frequency distribution at 450 K is marked with distinct peaks corresponding to the DMG, WMG and TS regions with corresponding minima in the free energy landscape at (Q_L_: 0.5; SASCN: 2.5), (0.4; SASCN: 7.5) and TS regions (Q_L_: 0.35; SASCN: 12.5 and Q_L_: 0.25; SASCN: 10.0) respectively.

At 450 K there was a significant increase in the ruggedness of the landscape (Fig 22) with minima appearing at the DMG (Q_L_: 0.5; SASCN: 2.5) and WMG regions (Q_L_: 0.4; SASCN: 7.5) and two closely spaced minima at TS (Q_L_: 0.35; SASCN: 12.5 and Q_L_: 0.25; SASCN: 10.0). Of the three the minima in the energy landscape the minima corresponding to the WMG region dominated the landscape.

**Fig 23:**
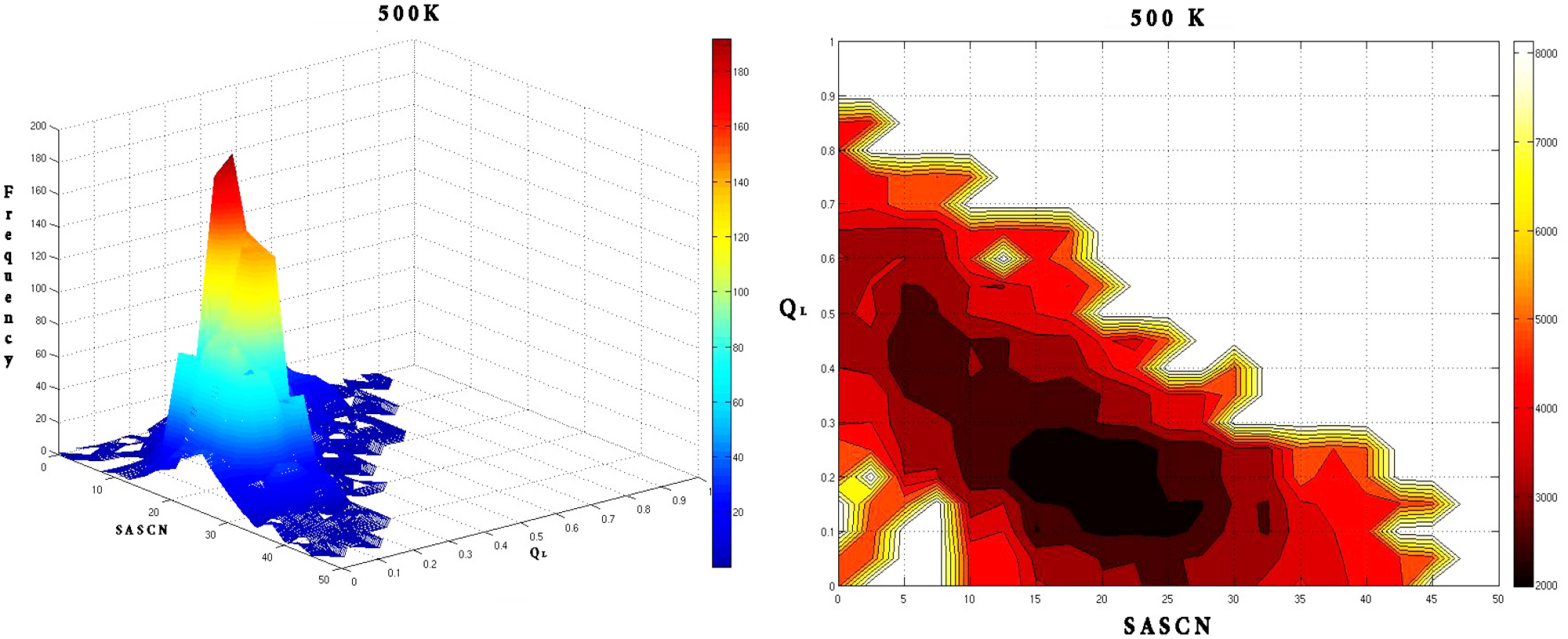
Frequency distribution and the corresponding free energy contour map at 500 K with reaction coordinates Q_L_ and SASCN. Free energy contour map at 500 K is characterized by a broad minimum around (Q_L_: 0.2; SASCN: 20) with a corresponding single peak for the frequency distribution.

At, 500K (Fig 23) there appeared to be a unique minimum centered at Q_L_: 0.2; SASCN: 20.

Notably, both the peaks in the frequency distributions and the minima in the corresponding energy landscapes were well within the criteria based on Q_L_—SASCN plots for different states (DMG, WMG etc.) which were determined based on time series analysis of network parameters and water interactions.

In most of the other 450K simulations (SIM2-SIM5) the WMG like regions were preferentially populated between 15–20ns, especially for 450KSIM3 and 450KSIM5 (Figure Q in S1 File) whereas DMG like states appeared only during the first 10ns (450KSIM2-5). Snapshots in the ‘TS’ region accumulated preferentially post 25ns in 450KSIM3 and 450KSIM5, whereas for 450KSIM2 and 450KSIM4 they seemed to accumulate post 30ns.

A similar analysis of the *Disnet*-SASCN plots led to the demarcation of similar regions in the plot such as ‘Native-Like’ (0 < SASCN < 5 & 0.3 <*Disnet* < 0.5); ‘DMG-Like’ (1 < SASCN < 5 & 0.5 <*Disnet* < 0.7); ‘WMG-Like’ (5 < SASCN < 10 & 0.7 <*Disnet* < 0.9); ‘TS’ (7.5 < SASCN < 20 & 0.8 <*Disnet* < 0.9) and the ‘Unfolded Region’ (SASCN>25 &*Disnet*> 0.9) (Figure R in S1 File).

One notable difference between *Disnet* and Q_L_ appeared to be the greater sensitivity, spread in *Disnet* values relative to Q_L_ with its fairly rapid elevation in beginning with the simulation even by 400 K. Otherwise the behavior of both measures in the context of the plot were mutually consistent (Figure Q, R in S1 File). Thus, as per the simulations the protein does sample DMG like states during the course of unfolding, the experimental observations of such states would perhaps correlate with the probability with which these states are sampled during the course of the simulation. For 450KSIM1 the relative probabilities of the ‘DMG’, ‘WMG’ and the ‘TS’ regions in the Q_L−_ SASCN plots(calculated by counting the number of snapshots in the specified regions divided by the total number of snapshots) were 0.08, 0.31 and 0.13 respectively. Thus it appears that the primary stable intermediate should probably be WMG like. However the procedure adopted in this work needs to be confirmed by simulations on multiple proteins to confirm the criteria adopted to distinguish between the DMG and the WMG states.

### Classical Multidimensional Scaling to identify the TS region

**Fig 24:**
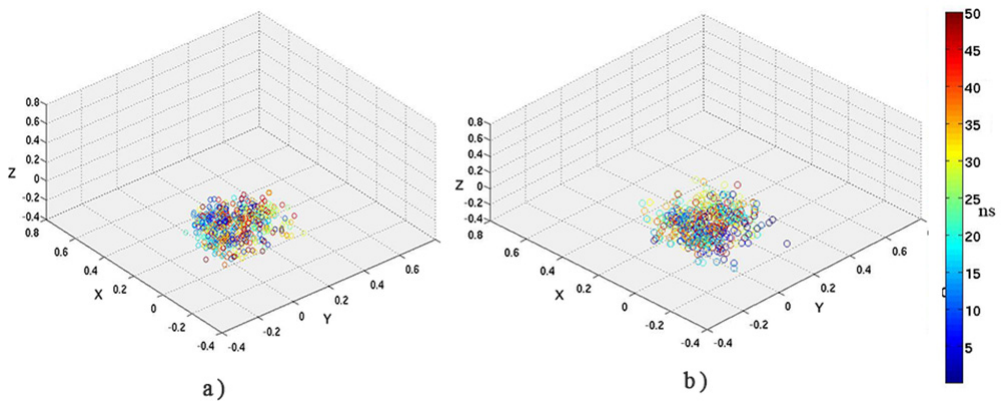
Multi-dimensional scaling based on *Disnet* for 310 and 400K. Coordinates derived by MDS, based on the metric *Disnet* at simulation temperatures of a) 310KSIM1 and b) 400KSIM1. The color bar for the plotted points represent snapshots at time intervals during the course of the simulation.

Classical multidimensional scaling (MDS) was utilized to transform (*Disnet* based) ‘distances’ into coordinates (**Materials & Methods**), in order to ascertain whether the distribution of points could enable the identification of the transition state region. Snapshots were sampled every 100 ps for every simulation block (at temperatures 310, 400 K etc.) and the matrix of inter-snapshot ‘distances’ (*Disnet*) converted to coordinates independently for each temperature by MDS. The distribution (Fig 24) at 310KSIM1 and 400KSIM1 were very similar, dominated by a unique dense cluster of points with a few outliers.

**Fig 25:**
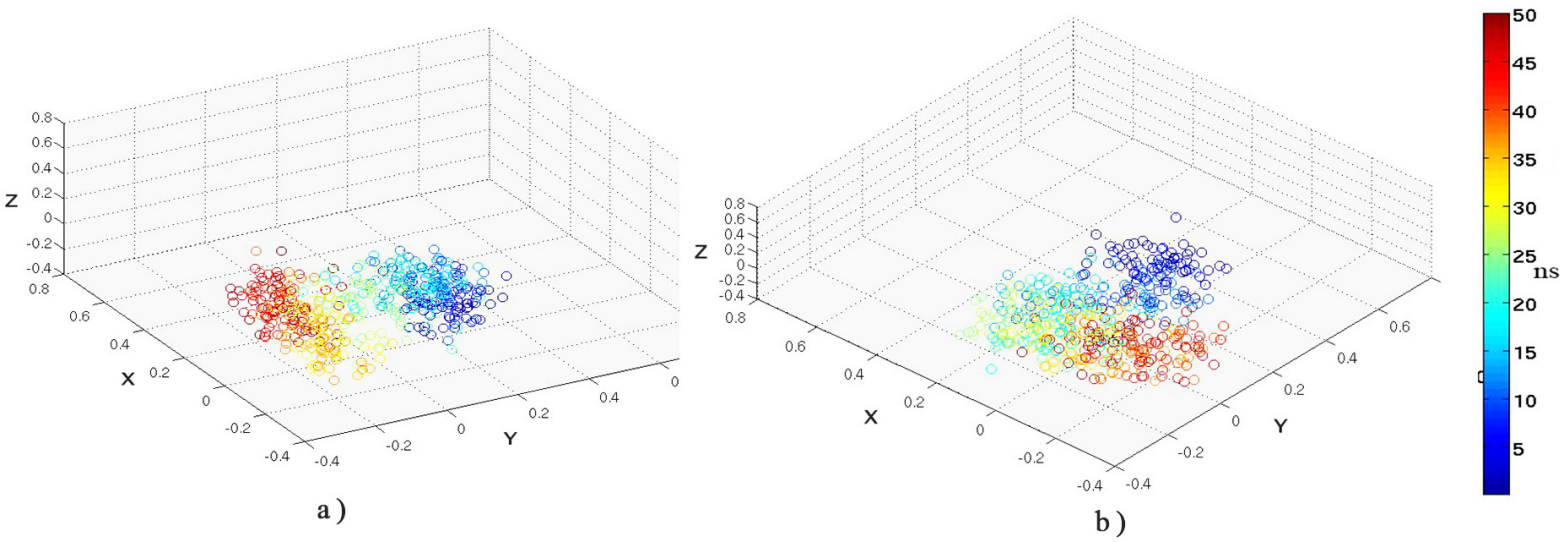
Multi-dimensional scaling based on *Disnet* for 450 and 500K. Coordinates derived by MDS, based on the metric *Disnet* at simulation temperatures of a) 450KSIM1 and b) 500KSIM1. The color bar for the plotted points denoting snapshots at indicated time intervals during the course of the simulation. The distribution splits into two distinct clusters before and after the transition state for 450 K.

The only difference between the distributions was the marginal increase in the number of outliers at 400KSIM1 (with respect to 310). The pattern for the 450KSIM1 simulation block was however substantially different, with the total number of points being partitioned into two distinct distributions (Fig 25); one retaining the characteristics of the previously observed dense cluster whereas the other fanning out into a widely dispersed array.

If a rapid expansion in the number of conformational states of the protein can be expected immediately subsequent to the transition state then the departure from the densely packed region of the plot (between 25 to 35 ns: Fig 25) could possibly be identified as exit from the transition state.

A dense cluster of points represents an ensemble of structures with very similar internal architecture. On the other hand, the wide dispersal of points denotes the dissolution of the compact core substituted by labile, non-native interactions in the interior of the molecule. By 500 K the entire distribution was dominated by a widely dispersed array of points indicative of a completely dissolved core.

## Conclusions

The dual use of surface complementarity measures and network based metrics provide several insights into the unfolding process which can be summarized as follows:

1. The initial unlocking of packing interactions in proteins leading to DMG states could be more common than has been hitherto recognized, though a wide variability in their stabilities for different proteins could of course be expected. Surface complementarity based metrics appear to be very sensitive global indicators to even slight perturbations in packing interactions and the probability of conformational states to occupy the different regions of the Q_L_/*Disnet*-SASCN plots could perhaps be exploited to predict whether DMG or WMG states were sufficiently probable to qualify as experimentally detectable intermediates in the protein unfolding pathway. In particular the criteria for DMG, WMG conformational states (Q_L_/*Disnet*-SASCN plots), naturally characterizes the different stages in protein unfolding. The effectiveness of the proposed computational method will obviously have to be experimentally confirmed in the future and also tested on the unfolding simulations of other proteins. Another question which urgently needs to be addressed is with regard to the nature of topological constraints in the surface contact networks which could give rise to stable DMG/WMG intermediates. To sum up, the calculations appear to indicate that in the course of unfolding DMG like states will be sampled as a natural sequence of events, however limited the temporal duration of such a sampling might be.
2. The unraveling of the SCN was a consequence of sequential disintegration of specific links in the network evident from following the pattern in their persistence values as a function of time and temperature. For cyclophilin in particular the relaxation of packing constraints about strategic nodes (31 PHE, 85 ILE) and the dissociation of helix H2 from the core or the main body of the protein appeared to be the first step in it’s unfolding. Even at 400K when the hydrophobic core, the β barrel and the association of the other helix (H1) was reasonably intact, greater conformational flexibility was exhibited by the surface contact network (SCN) spanning the core, with respect to the native structure (simulated at 310 K). This increased dynamic fluctuation was captured by the metrics persf, dlf, Q_L_ and the examination of the ‘persistence’ in case of individual contacts. At this stage the core of cyclophilin was wholly inaccessible to the solvent, native like and compact. Thus, the initiation of unfolding could be concomitant to a subtle enhancement in network fluctuations. Further ‘persistence networks’ provided a convenient method to identify possible critical or strategic interactions while unfolding.
3. Entry into the transition state appeared to be concomitant to the abrupt destabilization of strategic high persistence contacts. A significant fraction of these contacts could centre about ‘hubs’ such as 151 PHE and 59 PHE. The main transition state of the unfolding appeared between 22–30 ns in the 450 K SIM1 simulation block, indicated by a sudden rise in all the network parameters (*Disnet*, persf, dlf) subsequent to which there was a collapse of the surface contact network and rapid expansion into non—native conformational states. Examination of ‘persistence’ networks indicated key residues whose side chains interlocked to form a triplet and quadruplet clique which could possibly be the crucial step in folding. Prior to the transition state breakage of links was limited to local regions whereas subsequent to TS disintegration of the network was highly cooperative and pervasive.
4. Conversion of inter—snapshot ‘distances’ by MDS techniques, based on network based metrics appears to clearly demarcate the entry into and exit from the transition state ensemble.

Thus, metrics based on surface contact networks appears to possess definite advantages suitable for dissecting the unfolding trajectory into appropriate stages (DMG, WMG, TS) especially involving locking/unlocking of side chain residues in the MG regions.

## Supporting Information

S1 File Supporting Information file containing figures and tables. A single PDF file which contains all the supporting information’s consisting of figures and tables (with figure and table legends). Figure A, Cα RMSD of all residues and the core residues. Cα RMSD of all residues and the core residues for the other simulations at 310, 400, 450 and 500 K with crystal structure of LdCyp (2HAQ) as a reference, in panel (A) 310 K simulation sets with all residues. Color scheme: 310KSIM2ALL “black solid line”, 310KSIM3ALL “blue solid line”, 310KSIM4ALL “magenta solid line”, 310KSIM5ALL “cyan solid line”. Color scheme with only core residues 310KSIM2CORE “red solid line”, 310KSIM3CORE “green solid line”, 310KSIM4CORE “dark yellow solid line”, 310KSIM5CORE “brown solid line”. Similarly, for 400, 450, and 500 K in panels B, C and D respectively with identical color scheme. Figure B, RMSF values of all residues and for residues in the hydrophobic core. RMSF values for all residues from simulation sets a) 310KSIM1 “black solid line”, b) 400KSIM1 “red solid line”, c)450KSIM1 “blue solid line” and d) 500KSIM1 “green solid line”, whereas RMSF for core residues are plotted for a) 310KSIM1 “black solid line joined by black filled circles”, b) 400KSIM1 “red solid line joined by red filled circles”, c) 450KSIM1 “blue solid line joined by blue filled circles” and d) 500KSIM1 “green solid line joined by green filled circles”. Figure C, Comparison between *Disnet* and Q. Comparison of *Disnet* and Q with *Disnet* being plotted for 310KSIM1 “magenta solid line”, 400KSIM1 “dark yellow solid line”, 450KSIM1 “dark green solid line” and500KSIM1 “brown solid line”, whereas Q is plotted for 310KSIM1 “black solid line”, 400KSIM1 “red solid line”, 450KSIM1 “blue solid line” and 500KSIM1 “green solid line”. Figure D, *Disnet* values at different simulation temperatures of 310, 400 and 450 K (SIM2-SIM5) *Disnet* values for other simulation sets at a) 310KSIM2 “black solid line”, 310KSIM3 “red solid line”, 310KSIM4 “blue solid line”, 310KSIM5 “green solid line”. b) 400 K c) 450 K with identical color schemes. Figure E, Persfvalues at simulation temperature of 450 K (SIM2-SIM5). Persf values for simulations at 450 K plotted versus epochs, a) 450KSIM2“black solid line joined by black filled circles”b) 450KSIM3“red solid line joined by red filled circles”, c) 450KSIM4“blue solid line joined by blue filled circles” and 450KSIM5“green solid line joined by green filled circles”. Figure F, Dlf values at simulation temperature of 450 K (SIM2-SIM5). Dlf values for 450K simulation temperatures plotted versus epochs a) 450KSIM2“black solid line joined by black filled circles”b) 450KSIM3 “red solid line joined by red filled circles”, c) 450KSIM4“blue solid line joined by blue filled circles” and 450KSIM5“green solid line joined by green filled circles”. Figure G, Metric values averaged over 5 simulations along with their standard deviations. Metric Ca RMSD of core, *Disnet* and persf (panel a, panel b and panel c) were averaged over the 5 simulations (SIM1-SIM5) and their standard deviations estimated. The color scheme followed is same as above. Figure H, Consensus network diagram at 450 K (SIM1-SIM5), 6 ns. Persistence network diagram at 450 K, 6 ns depicting the disruption of link 31 PHE– 59 PHE, with residues (nodes) located on helix H1 colored in yellow, helix H2: red and strand residues: green. The completely disrupted links (absent in at least 4 simulations based on persistence cutoff of 0.4) are represented in red solid line, while the intermediate links (present in 2or 3 simulation sets out of 5) are represented by broken blue lines. Persistent links (present in at least 4 simulation sets) are represented by solid grey lines. Figure I, Consensus network diagram at 10 ns, 450 K (SIM1-SIM5). The network diagram depicts the disruption of majority of the links in L1 with some instability in the extended linear region L3 and in the quadruplet clique QC. The color scheme followed is same as above. Figure J, Consensus network diagram at 32 ns, 450 K (SIM1-SIM5). The network exhibits disruption of links 31 PHE- 134 PHE leading to the dissolution of one of the triplet cliques (31 PHE- 71 TYR- 134 PHE) formed due to the disruption of links in erstwhile quadruplet clique QC. The color scheme followed is same as above. Figure K, Consensus network diagram at 450 K (SIM1-SIM5), 38 ns. The network depicts the severance of link 29 VAL- 47 LEU that links QC with L2 apart from disruption in L1, QC, TC and L3. The color scheme followed is same as above. Figure L, Consensus network diagram at 500 K (SIM1-SIM5), 4 ns. The network diagram in the region of 4–8 ns at 500 K depicts heightened instability in TC. The color scheme followed is same as above. Figure M, Consensus network diagram at 500 K (SIM1-SIM5), 8 ns. The network depicts dissolution of one of the triplet cliques of QC (71 TYR-59 PHE- 134 PHE) due to disruption of link 59 PHE- 71 TYR. The color scheme followed is same as above. Figure N, Consensus network diagram at 500 K (SIM1-SIM5), 10 ns. The network diagram demonstrates the dissolution link 31 PHE– 71 TYR in erstwhile QC along with pronounced disruption in all the regions of the network (L1, QC, TC and L3). The color scheme followed is same as above. Figure O, Estimation of protein core solvation. The average number of waters (for every epoch: 2 ns) was calculated with a distance cutoff of 5Åfrom the side chain atoms of the core for 450KSIM1 “red solid line joined by red filled circles” and 500KSIM1 “black solid line joined by black filled circles”. Figure P, Q_L_-SASCN plots at different simulation temperatures. Fraction of native links (Q_L_) v/s SASCN plots at a) 310 K b) 400 K c) 450 K and d) 500 K to identify the ‘Native-like’, ‘DMG-like’, ‘WMG-like’ and the ‘TS’ regions. The color bar for the plotted points represents snapshots at time intervals during the course of the simulation. Figure Q, Q_L_-SASCN plots at 450K (SIM2-SIM5). Fraction of native links Q_L_-SASCN plots for 450 K a) 450KSIM2 b) 450KSIM3 c) 450KSIM4 and d) 450KSIM5 to identify the ‘native-like’, ‘DMG-like’, ‘WMG-like’ and the ‘TS’ snapshots. Figure R, *Disnet*-SASCN plots at different simulation temperatures. *Disnet*-SASCN plots at a) 310 K b) 400 K c) 450 K and d) 500 K to identify the ‘native, ‘DMG like’, ‘WMG like’ and the ‘TS’ regions. The color bar for the plotted points represents snapshots at time intervals during the course of the simulation. Table A, Set of contacts of S1 (core-core), S2 (helix1-core) and S3 (helix2-core), with the secondary structural elements in parentheses, S-Strands, H1-helix1, H2-helix2 and L-loops. Table B, Average Cα RMSD of simulations with all the residues (SIM_ALL) of LdCyp and with only the core residues (SIM_CORE)for other simulation sets (for temperatures 310, 400, 450, 500K) with the standard deviations in parentheses. Table C, RMSF values of core residues, all residues and non-core residues with standard deviations in parentheses. Table D, <*Disnet*> values for different simulation temperatures for the 310, 400, 450 and 500K calculated with native crystal structure (2HAQ) as baseline averaged over the entire simulation block of 50ns with the standard deviation given in parentheses. Table E, Cross-Correlation values between Q and *Disnet* at different simulation temperatures (SIM1-SIM5). Table F, Dlf and persf values averaged over the entire simulation block (between 2–50ns) for the hydrophobic core of LdCyp at different simulation temperatures for (450KSIM2, 450KSIM3, 450KSIM4 and 450KSIM5). The standard deviations are given in parentheses.
(PDF)

S1 Movie Movie Caption: “Unfolding of cyclophilin from Leishmania Donovani (LdCyp)”. (AVI)
