# Supplementary material for: The Unfolding MD Simulations of Cyclophilin: Analyzed by Surface Contact Networks and Their Associated Metrics
Source: PLoS One. 2015 Nov 6;10(11):e0142173. doi: 10.1371/journal.pone.0142173 (PMC4636149; doi:10.1371/journal.pone.0142173)
Supplement: S1 File — A single PDF file which contains all the supporting information’s consisting of figures and tables (with figure and table legends). Figure A, Cα RMSD of all residues and the core residues. Cα RMSD of all residues and the core residues for the other simulations at 310, 400, 450 and 500 K with crystal structure of LdCyp (2HAQ) as a reference, in panel (A) 310 K simulation sets with all residues. Color scheme: 310KSIM2ALL “black solid line”, 310KSIM3ALL “blue solid line”, 310KSIM4ALL “magenta solid line”, 310KSIM5ALL “cyan solid line”. Color scheme with only core residues 310KSIM2CORE “red solid line”, 310KSIM3CORE “green solid line”, 310KSIM4CORE “dark yellow solid line”, 310KSIM5CORE “brown solid line”. Similarly, for 400, 450, and 500 K in panels B, C and D respectively with identical color scheme. Figure B, RMSF values of all residues and for residues in the hydrophobic core. RMSF values for all residues from simulation sets a) 310KSIM1 “black solid line”, b) 400KSIM1 “red solid line”, c)450KSIM1 “blue solid line” and d) 500KSIM1 “green solid line”, whereas RMSF for core residues are plotted for a) 310KSIM1 “black solid line joined by black filled circles”, b) 400KSIM1 “red solid line joined by red filled circles”, c) 450KSIM1 “blue solid line joined by blue filled circles” and d) 500KSIM1 “green solid line joined by green filled circles”. Figure C, Comparison between Disnet and Q. Comparison of Disnet and Q with Disnet being plotted for 310KSIM1 “magenta solid line”, 400KSIM1 “dark yellow solid line”, 450KSIM1 “dark green solid line” and500KSIM1 “brown solid line”, whereas Q is plotted for 310KSIM1 “black solid line”, 400KSIM1 “red solid line”, 450KSIM1 “blue solid line” and 500KSIM1 “green solid line”. Figure D, Disnet values at different simulation temperatures of 310, 400 and 450 K (SIM2-SIM5) Disnet values for other simulation sets at a) 310KSIM2 “black solid line”, 310KSIM3 “red solid line”, 310KSIM4 “blue solid line”, 310KSIM5 “green solid line”. b) 400 K c) [file pone.0142173.s001.pdf]

Supporting Information

Supporting Figures

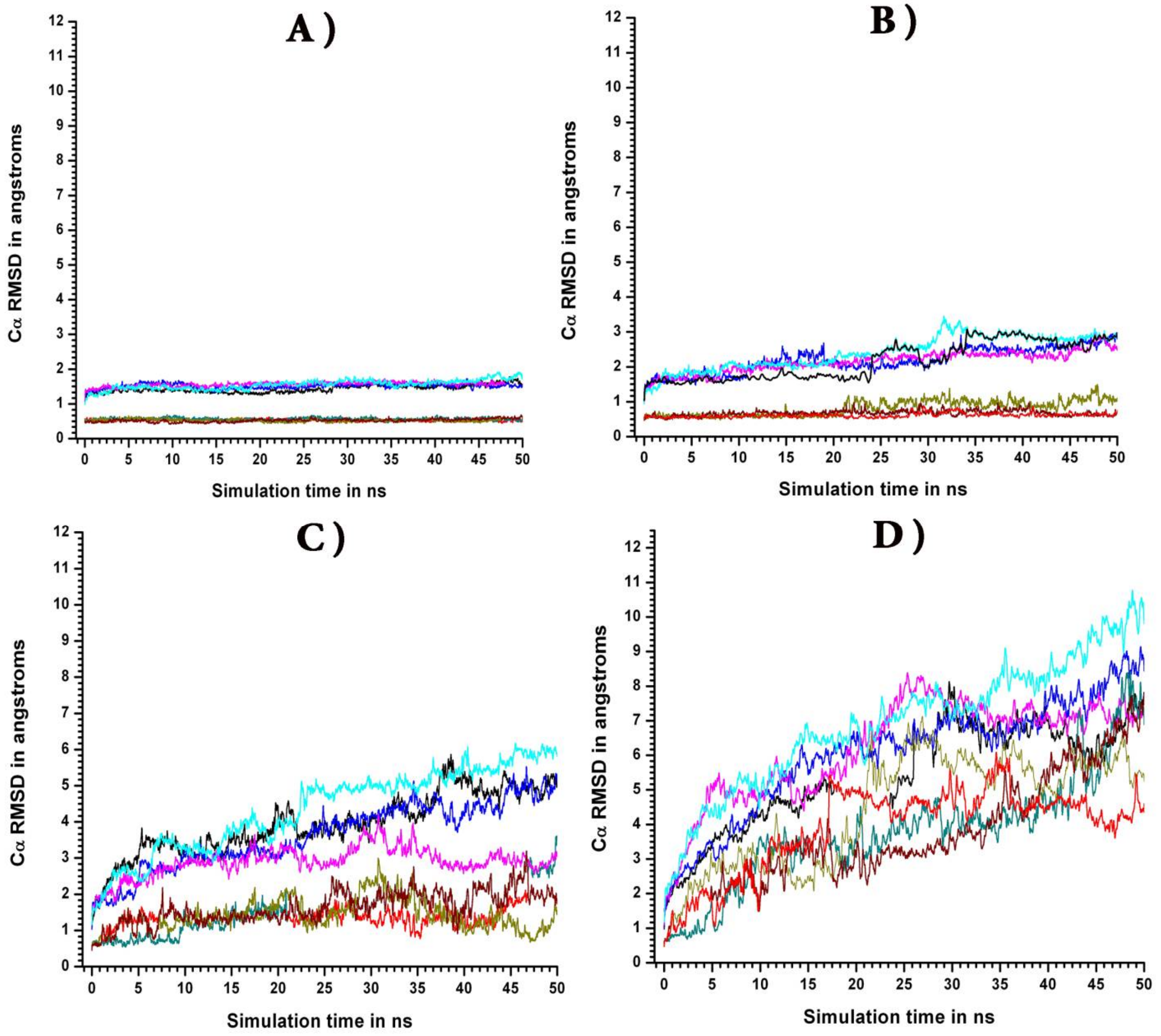

Figure A. Cα RMSD of all residues and the core residues. Cα RMSD of all residues and the core residues for the other simulations at 310, 400, 450 and 500 K with crystal structure of LdCyp (2HAQ) as a reference, in panel (A) 310 K simulation sets with all residues. Color scheme: 310KSIM2ALL "black solid line", 310KSIM3ALL "blue solid line", 310KSIM4ALL "magenta solid line", 310KSIM5ALL "cyan solid line". Color scheme with only core residues 310KSIM2CORE "red solid line", 310KSIM3CORE "green solid line", 310KSIM4CORE "dark

yellow solid line", 310KSIM5CORE "brown solid line". Similarly, for 400, 450, and 500 K in panels B, C and D respectively with identical color scheme.

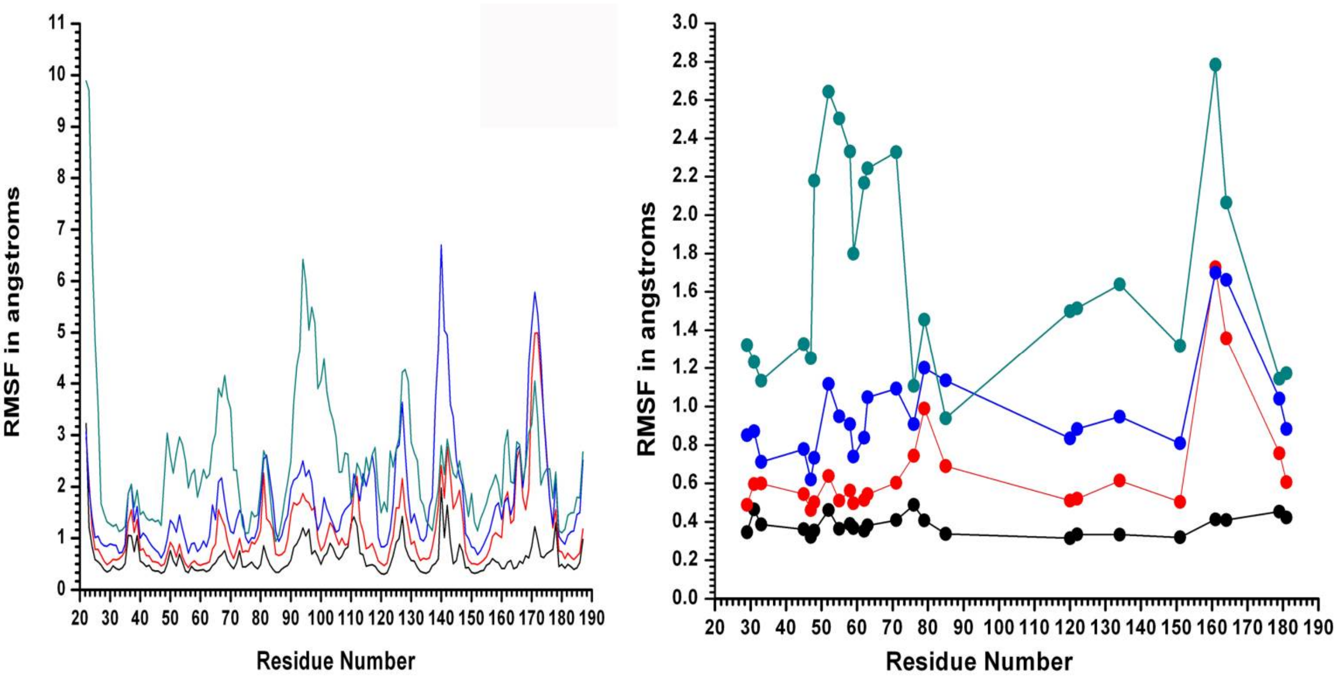

Figure B. RMSF values of all residues and for residues in the hydrophobic core. RMSF values for all residues from simulation sets a) 310KSIM1 "black solid line", b) 400KSIM1 "red solid line", c) 450KSIM1 "blue solid line" and d) 500KSIM1 "green solid line", whereas RMSF for core residues are plotted for a) 310KSIM1 "black solid line joined by black filled circles", b) 400KSIM1 "red solid line joined by red filled circles", c) 450KSIM1 "blue solid line joined by blue filled circles" and d) 500KSIM1 "green solid line joined by green filled circles".

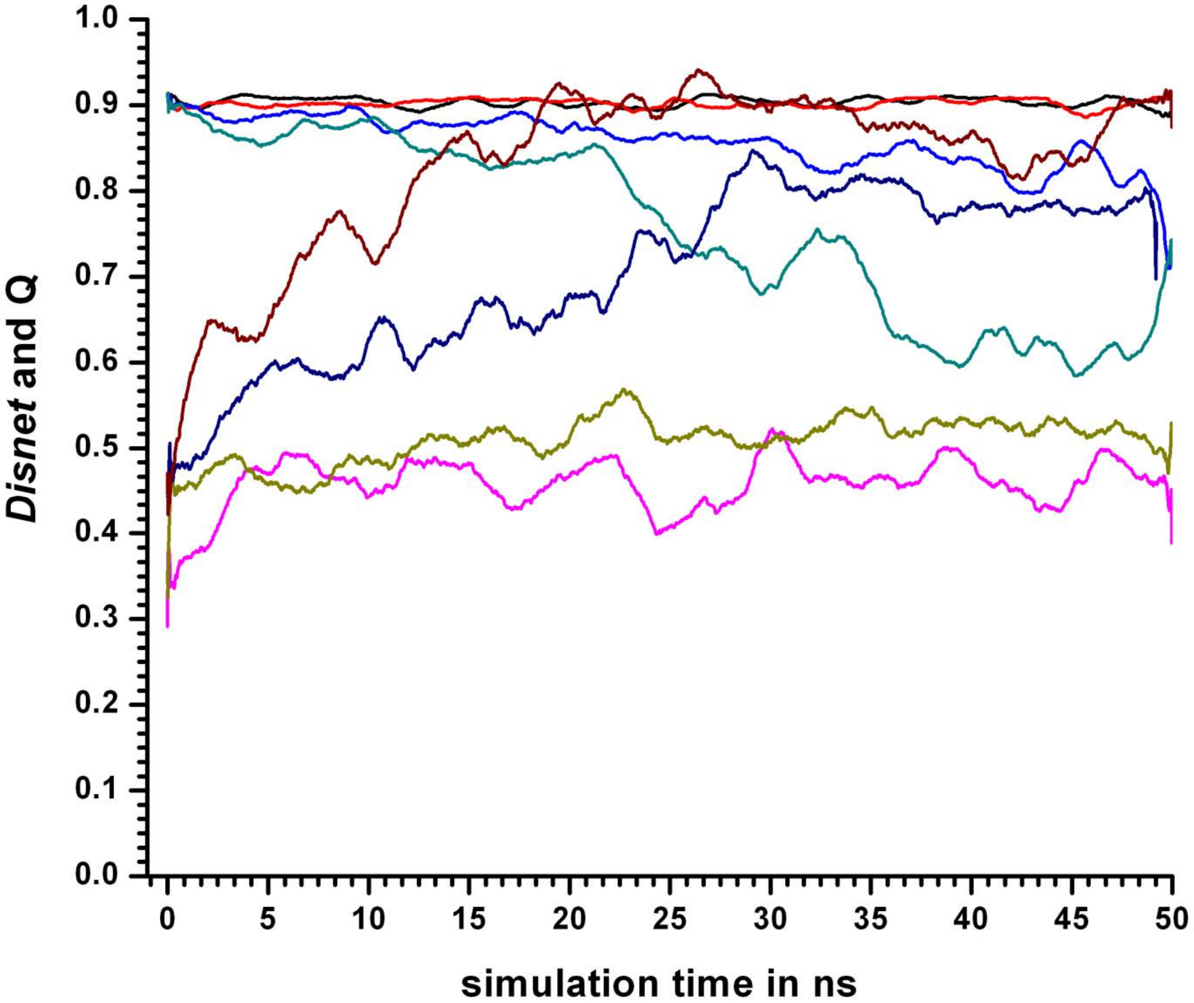

Figure C. Comparison between *Disnet* and Q.Comparison of *Disnet* and Q with *Disnet* being plotted for 310KSIM1 "magenta solid line", 400KSIM1 "dark yellow solid line", 450KSIM1 "dark green solid line" and 500KSIM1 "brown solid line", whereas Q is plotted for 310KSIM1 "black solid line", 400KSIM1 "red solid line", 450KSIM1 "blue solid line" and 500KSIM1 "green solid line".

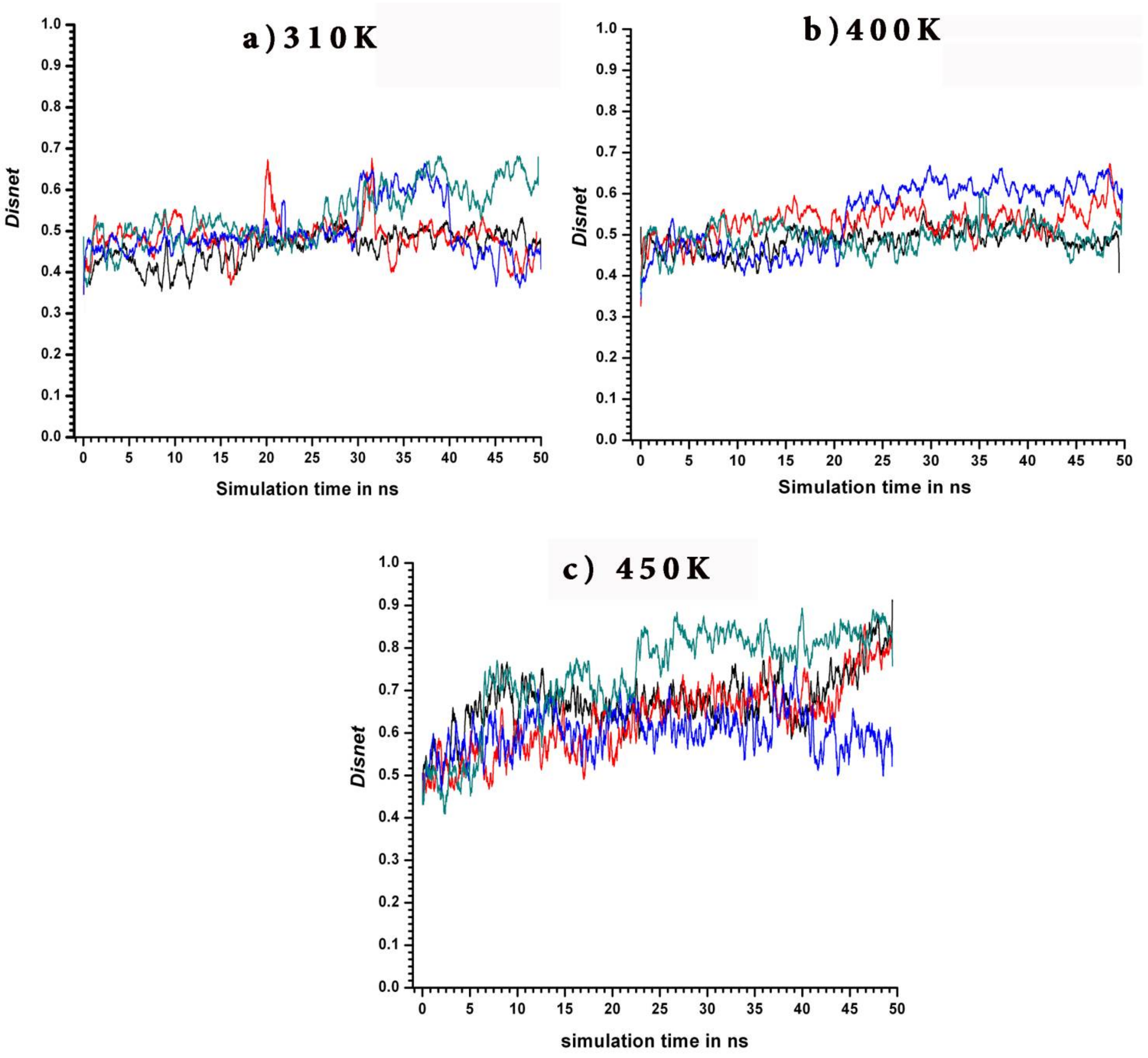

Figure D. ***Disnet*** **values at different simulation temperatures of 310, 400 and 450 K (SIM2-SIM5)** *Disnet*values for other simulation sets at a) 310KSIM2 “black solid line”, 310KSIM3 “red solid line”, 310KSIM4 “blue solid line”, 310KSIM5 “green solid line”. b) 400 K c) 450 K with identical color schemes.

.

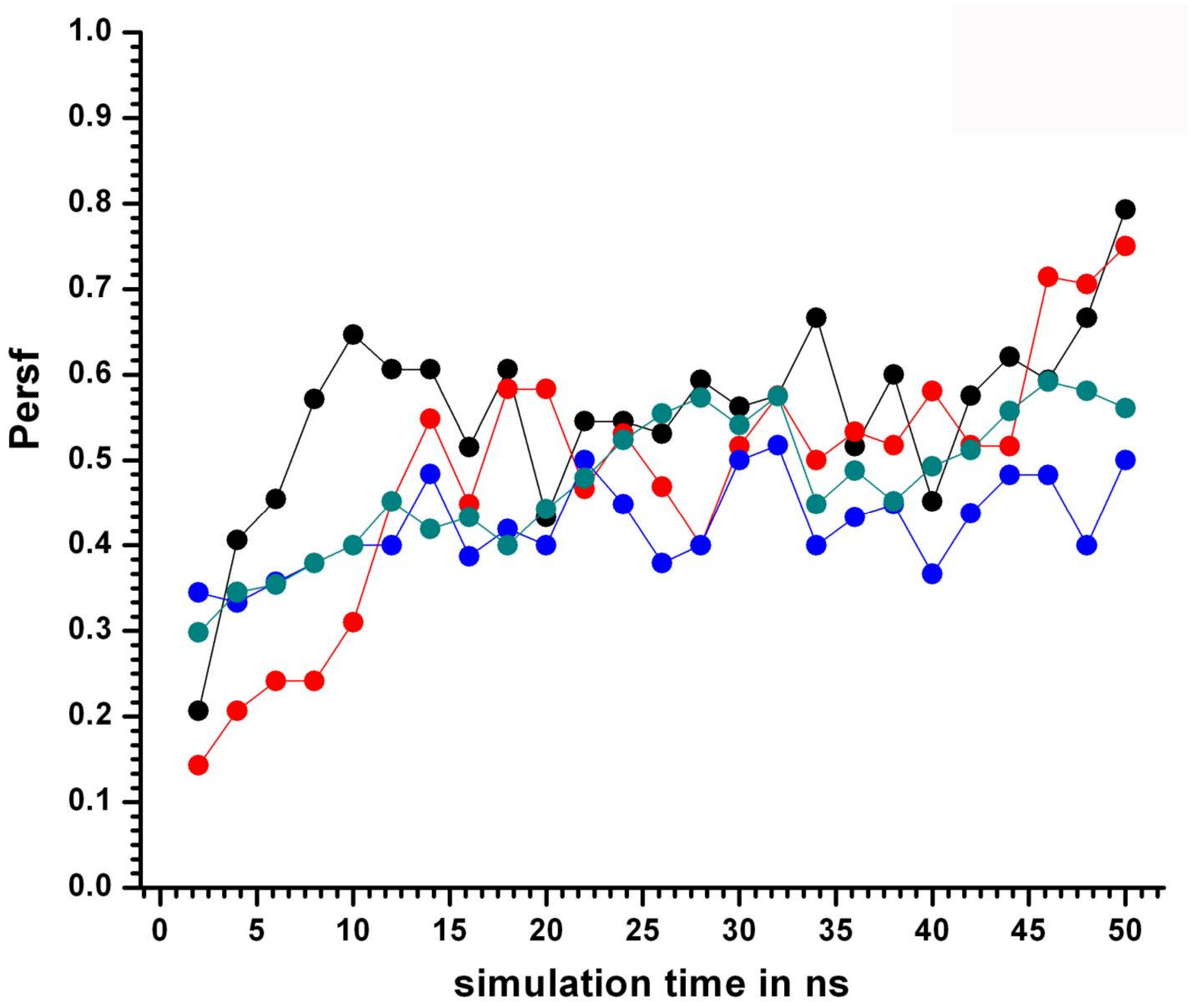

Figure E. Persf values at simulation temperature of 450 K (SIM2-SIM5). Persf values for simulations at 450 K plotted versus epochs, a) 450KSIM2 “black solid line joined by black filled circles” b) 450KSIM3 “red solid line joined by red filled circles”, c) 450KSIM4 “blue solid line joined by blue filled circles” and 450KSIM5 “green solid line joined by green filled circles”.

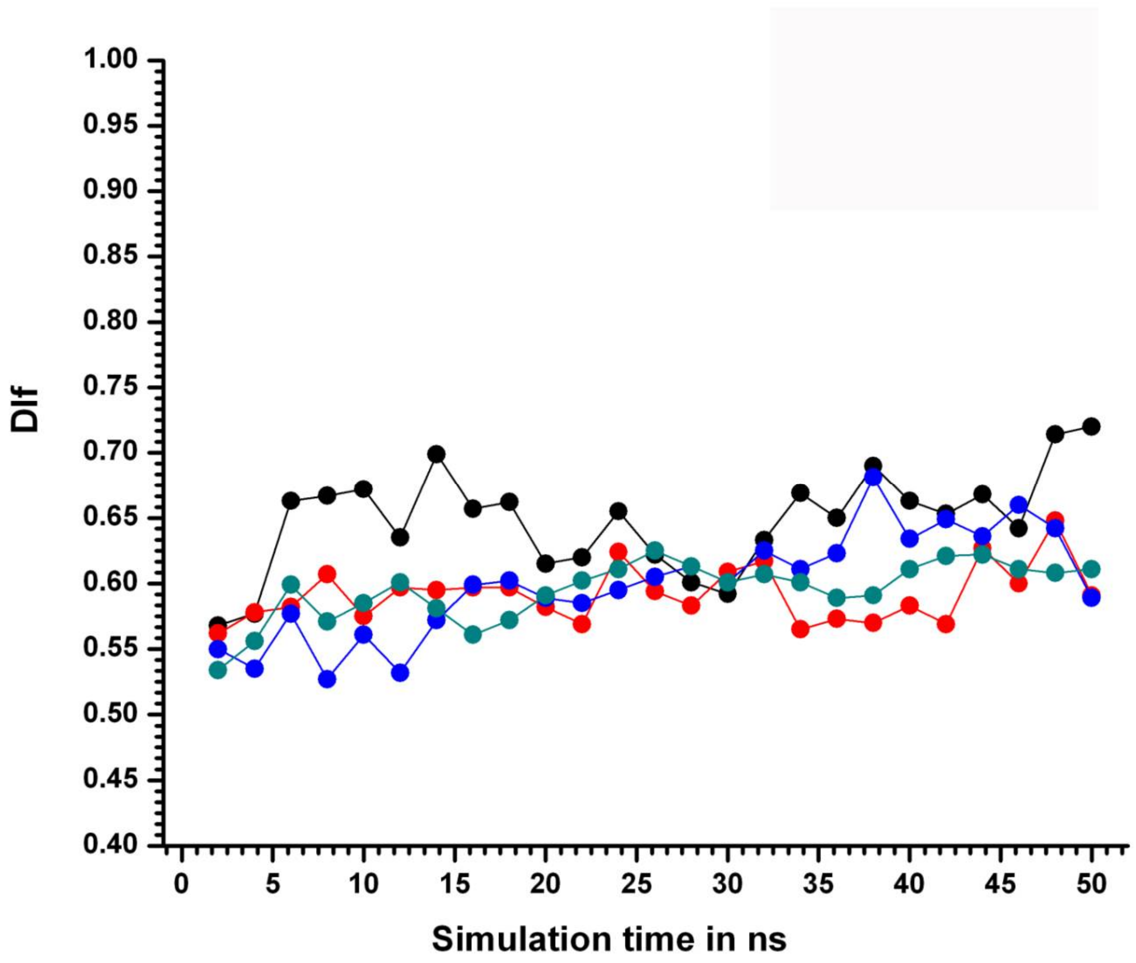

Figure F. Dlf values at simulation temperature of 450 K (SIM2-SIM5). Dlf values for 450K simulation temperatures plotted versus epochs a) 450KSIM2 “black solid line joined by black filled circles” b) 450KSIM3 “red solid line joined by red filled circles”, c) 450KSIM4 “blue solid line joined by blue filled circles” and 450KSIM5 “green solid line joined by green filled circles”.

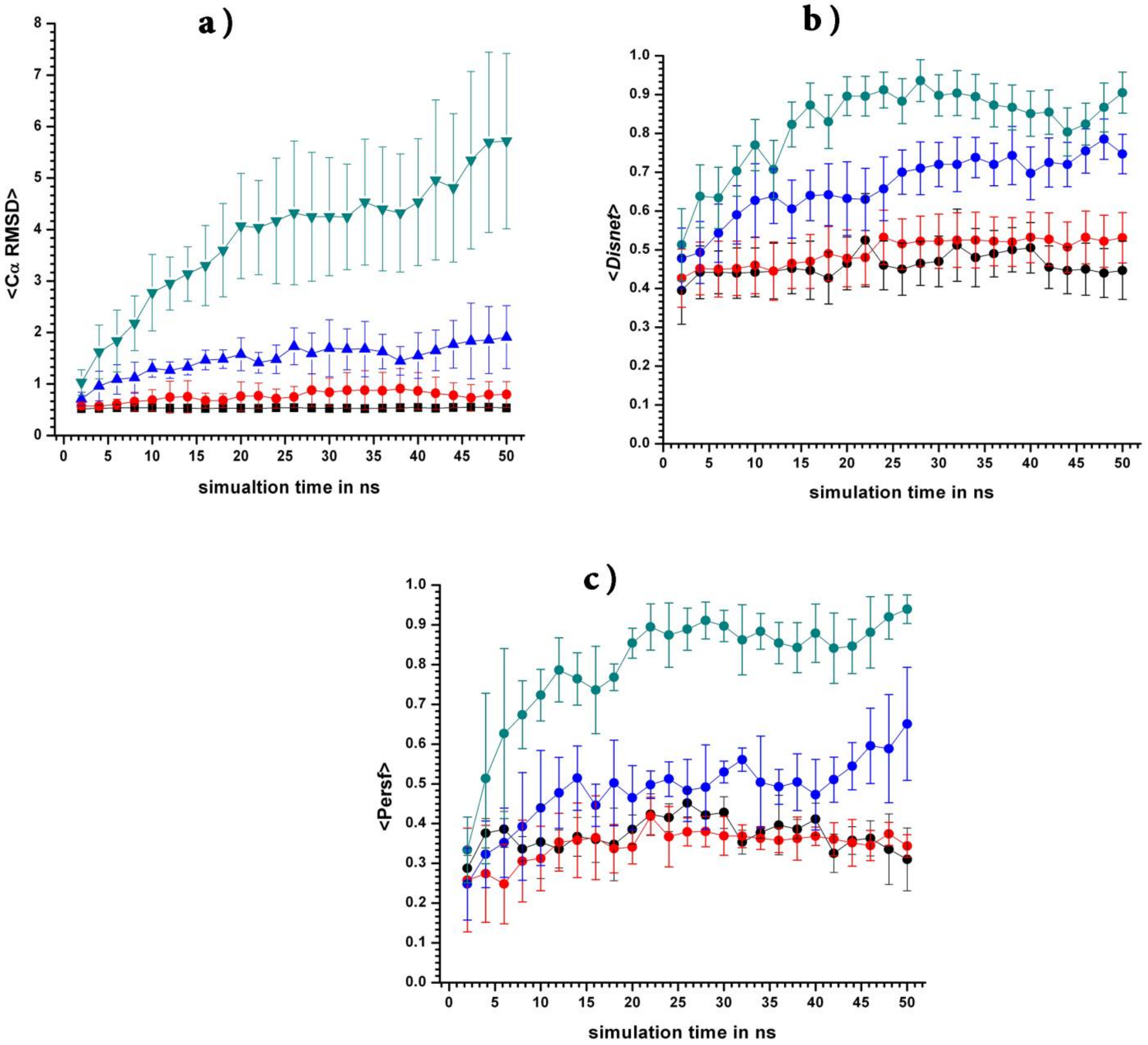

Figure G. Metric values averaged over 5 simulations along with their standard deviations. Metric Ca RMSD of core, *Disnet* and persf (panel a, panel b and panel c) were averaged over the 5 simulations (SIM1-SIM5) and their standard deviations estimated**.** The color scheme followed is same as above.

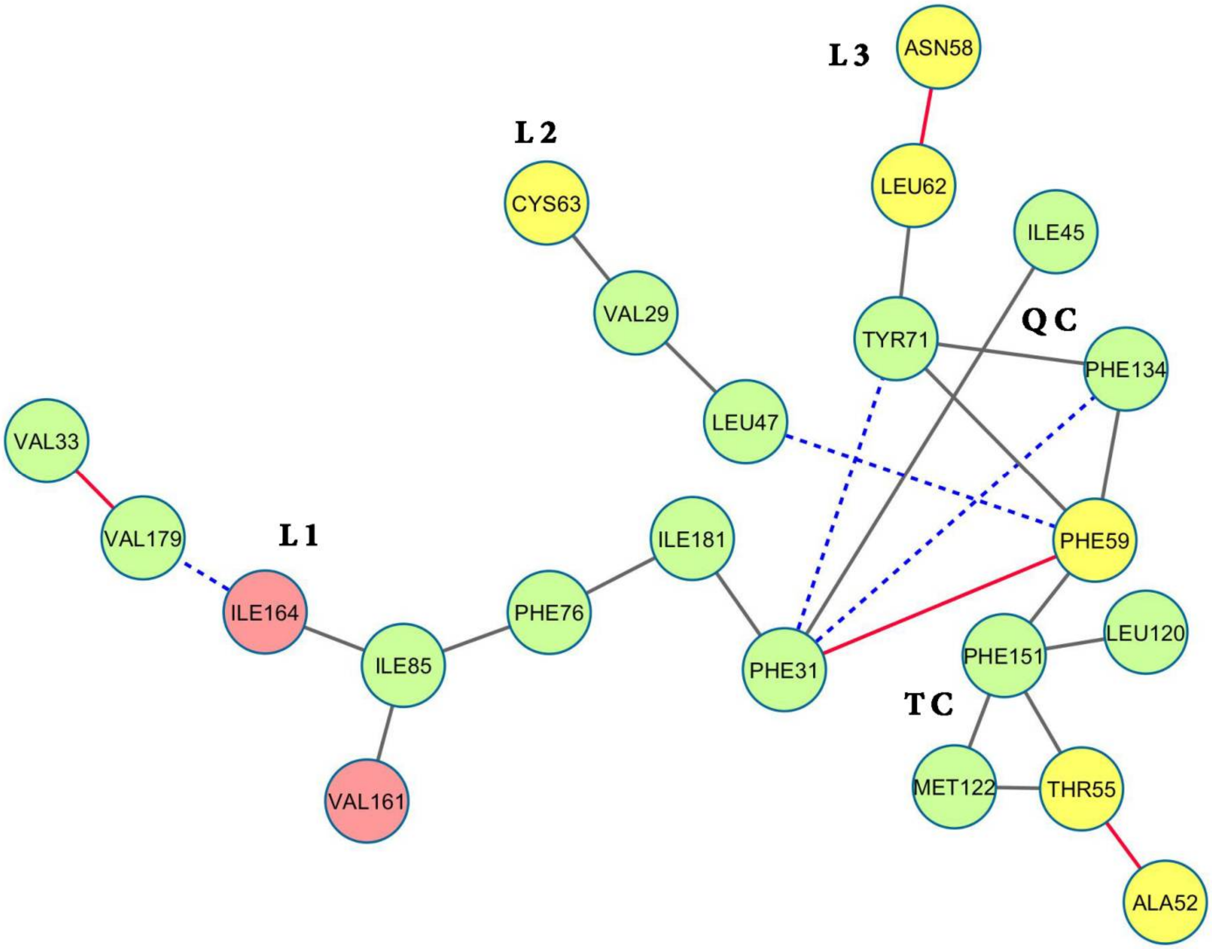

Figure H. Consensus network diagram at 450 K (SIM1-SIM5), 6 ns. Persistence network diagram at 450 K, 6 ns depicting the disruption of link 31 PHE – 59 PHE, with residues (nodes) located on helix H1 colored in yellow, helix H2: red and strand residues: green. The completely disrupted links (absent in at least 4 simulations based on persistence cutoff of 0.4) are represented in red solid line, while the intermediate links (present in 2or 3 simulation sets out of 5) are represented by broken blue lines. Persistent links (present in at least 4 simulation sets) are represented by solid grey lines.

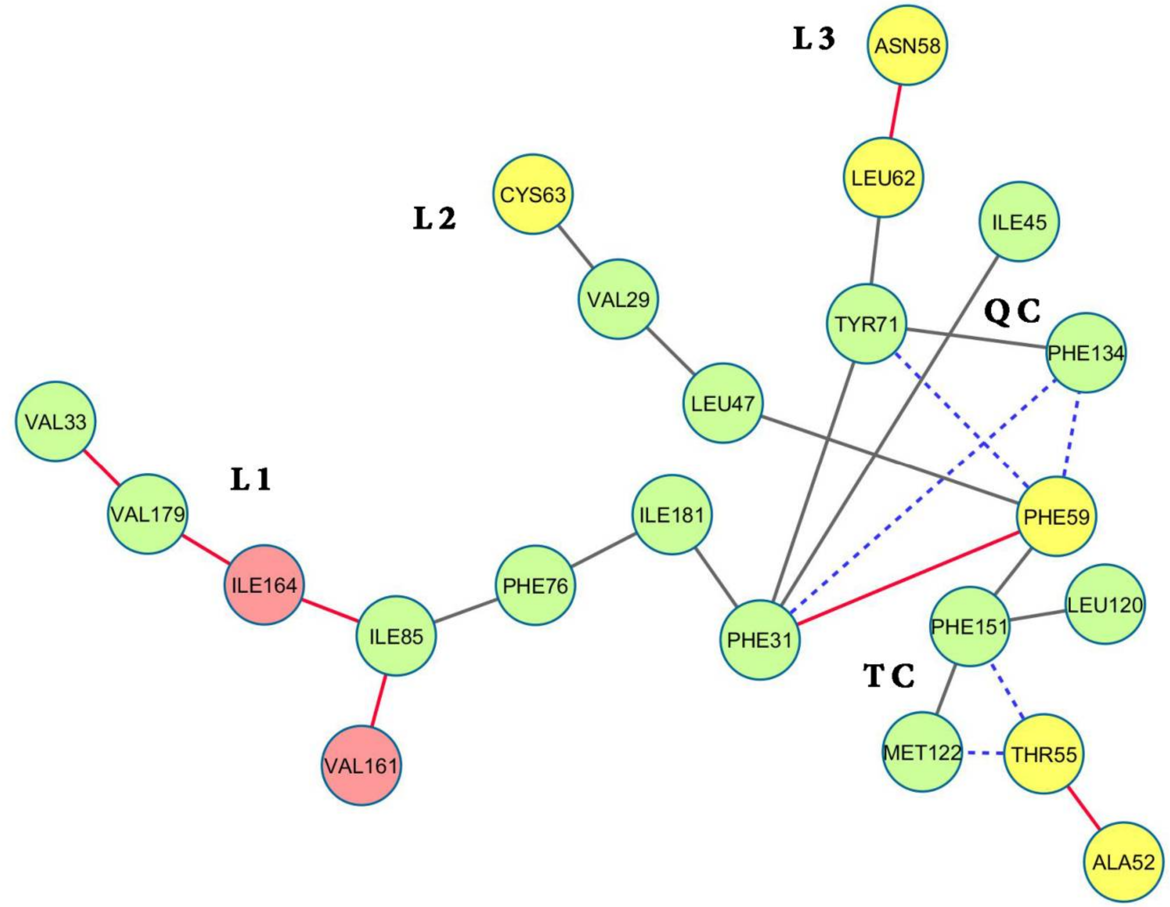

Figure I. Consensus network diagram at 10 ns, 450 K (SIM1-SIM5). The network diagram depicts the disruption of majority of the links in L1 with some instability in the extended linear region L3 and in the quadruplet clique QC. The color scheme followed is same as above.

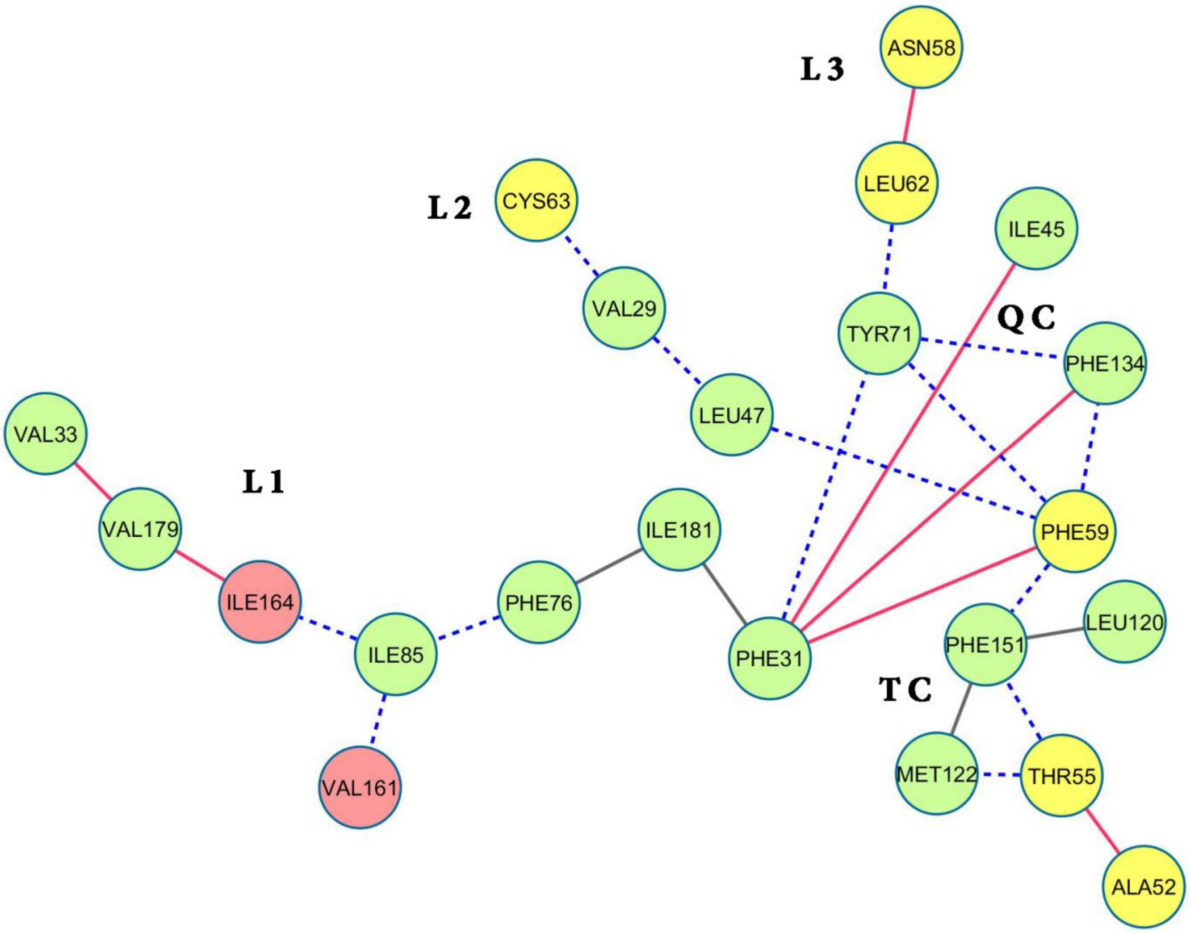

Figure J. Consensus network diagram at 32 ns, 450 K (SIM1-SIM5). The network exhibits disruption of links 31 PHE- 134 PHE leading to the dissolution of one of the triplet cliques (31 PHE- 71 TYR- 134 PHE) formed due to the disruption of links in erstwhile quadruplet clique QC. The color scheme followed is same as above.

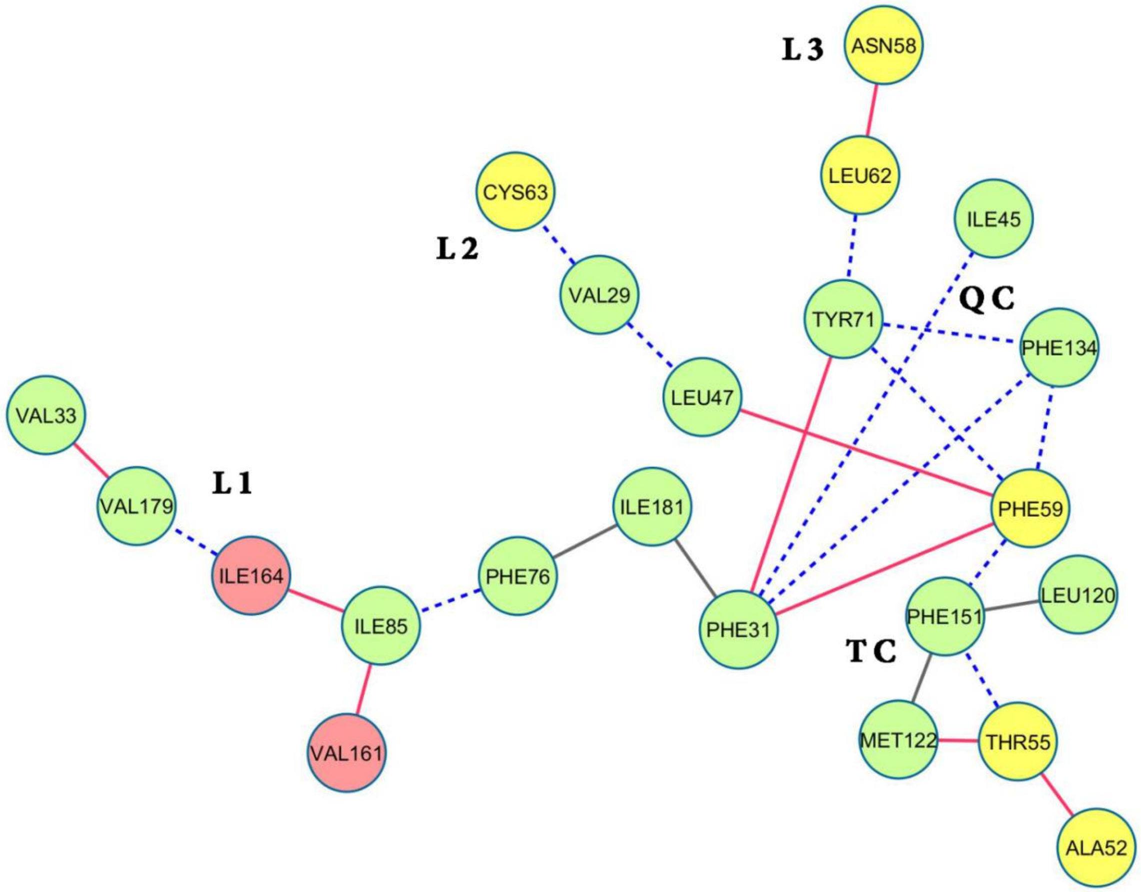

Figure K. Consensus network diagram at 450 K (SIM1-SIM5), 38 ns. The network depicts the severance of link 29 VAL- 47 LEU that links QC with L2 apart from disruption in L1, QC, TC and L3. The color scheme followed is same as above.

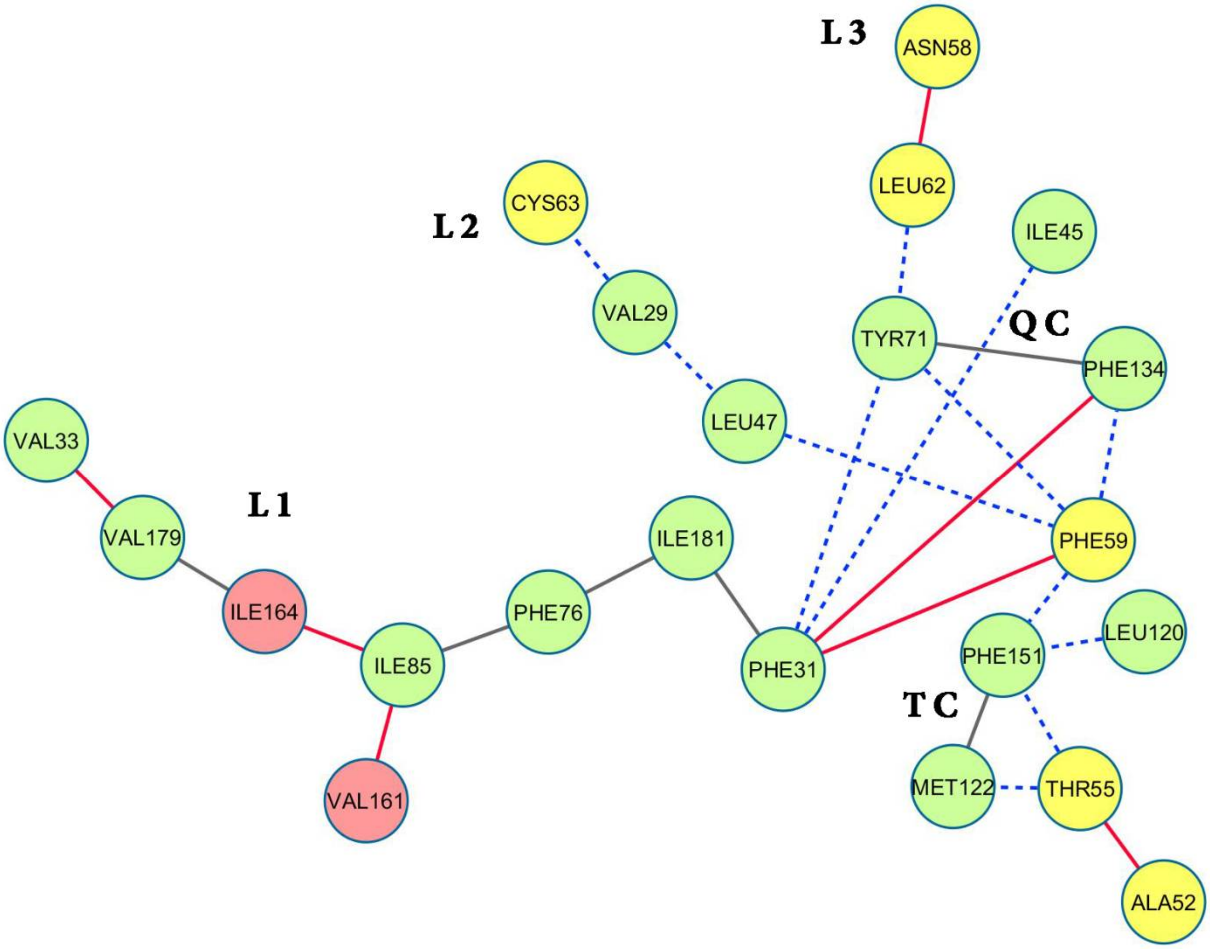

Figure L. Consensus network diagram at 500 K (SIM1-SIM5), 4 ns. The network diagram in the region of 4-8 ns at 500 K depicts heightened instability in TC. The color scheme followed is same as above.

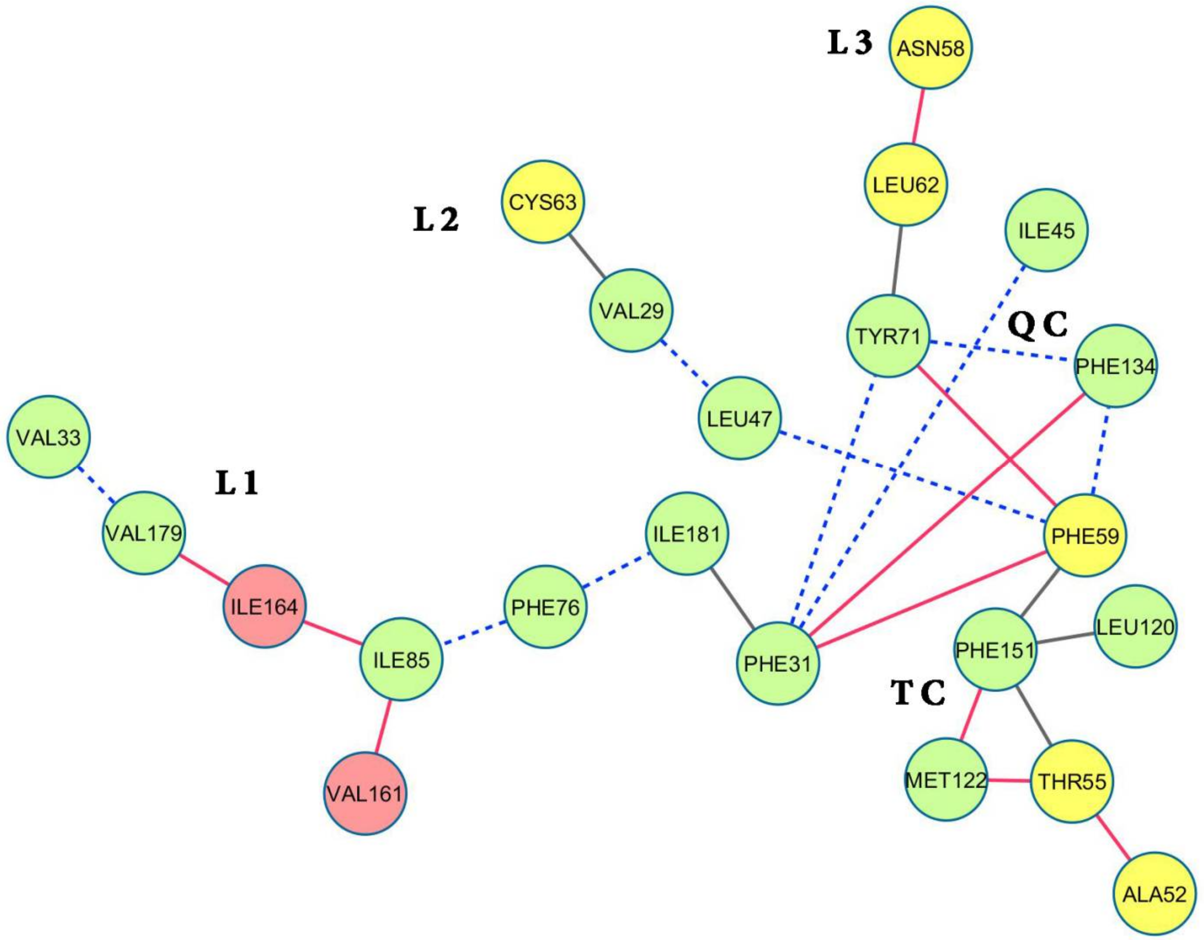

Figure M. Consensus network diagram at 500 K (SIM1-SIM5), 8 ns. The network depicts dissolution of one of the triplet cliques of QC (71 TYR-59 PHE- 134 PHE) due to disruption of link 59 PHE- 71 TYR. The color scheme followed is same as above.

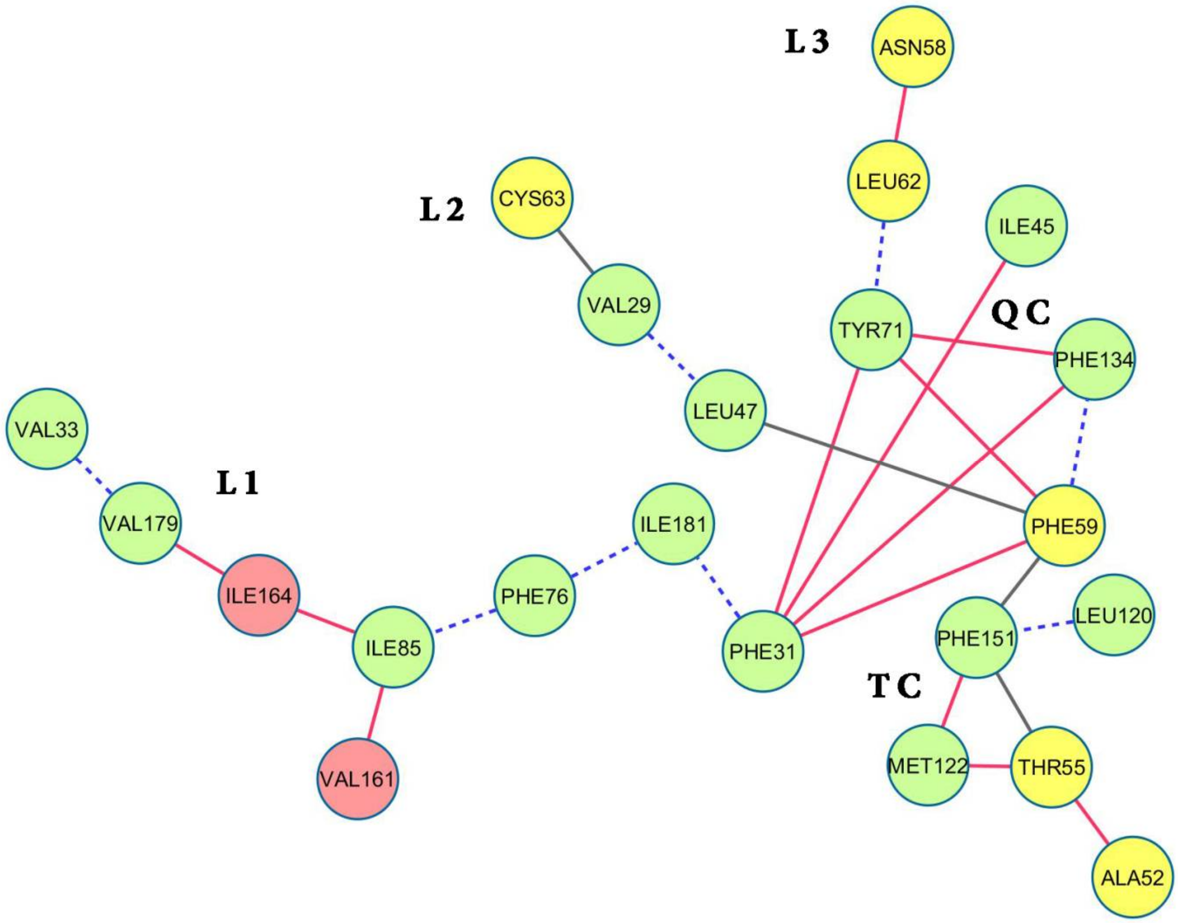

Figure N. Consensus network diagram at 500 K (SIM1-SIM5), 10 ns. The network diagram demonstrates the dissolution link 31 PHE – 71 TYR in erstwhile QC along with pronounced disruption in all the regions of the network (L1, QC, TC and L3). The color scheme followed is same as above.

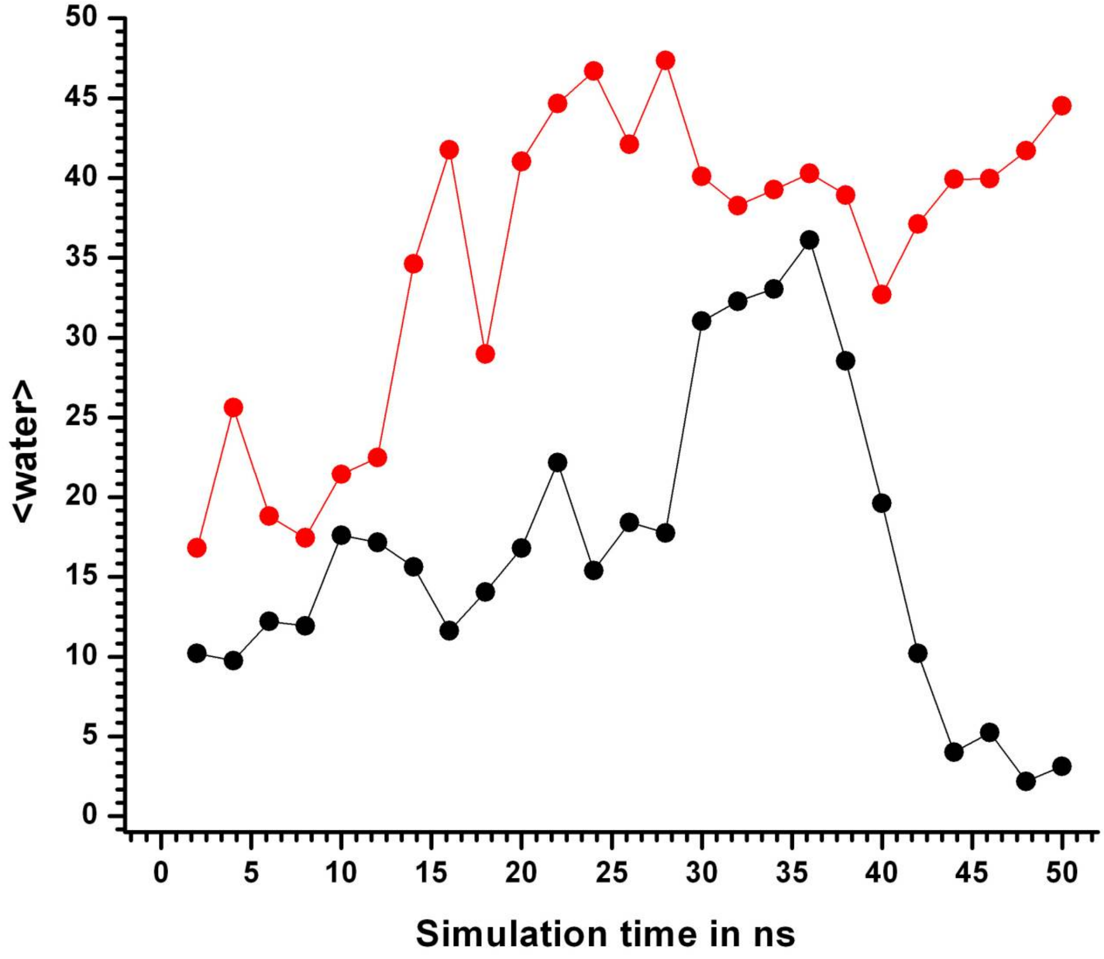

Figure O. Estimation of protein core solvation. The average number of waters (for every epoch: 2 ns) was calculated with a distance cutoff of 5Åfrom the side chain atoms of the core for 450KSIM1 “red solid line joined by red filled circles” and 500KSIM1 “black solid line joined by black filled circles”.

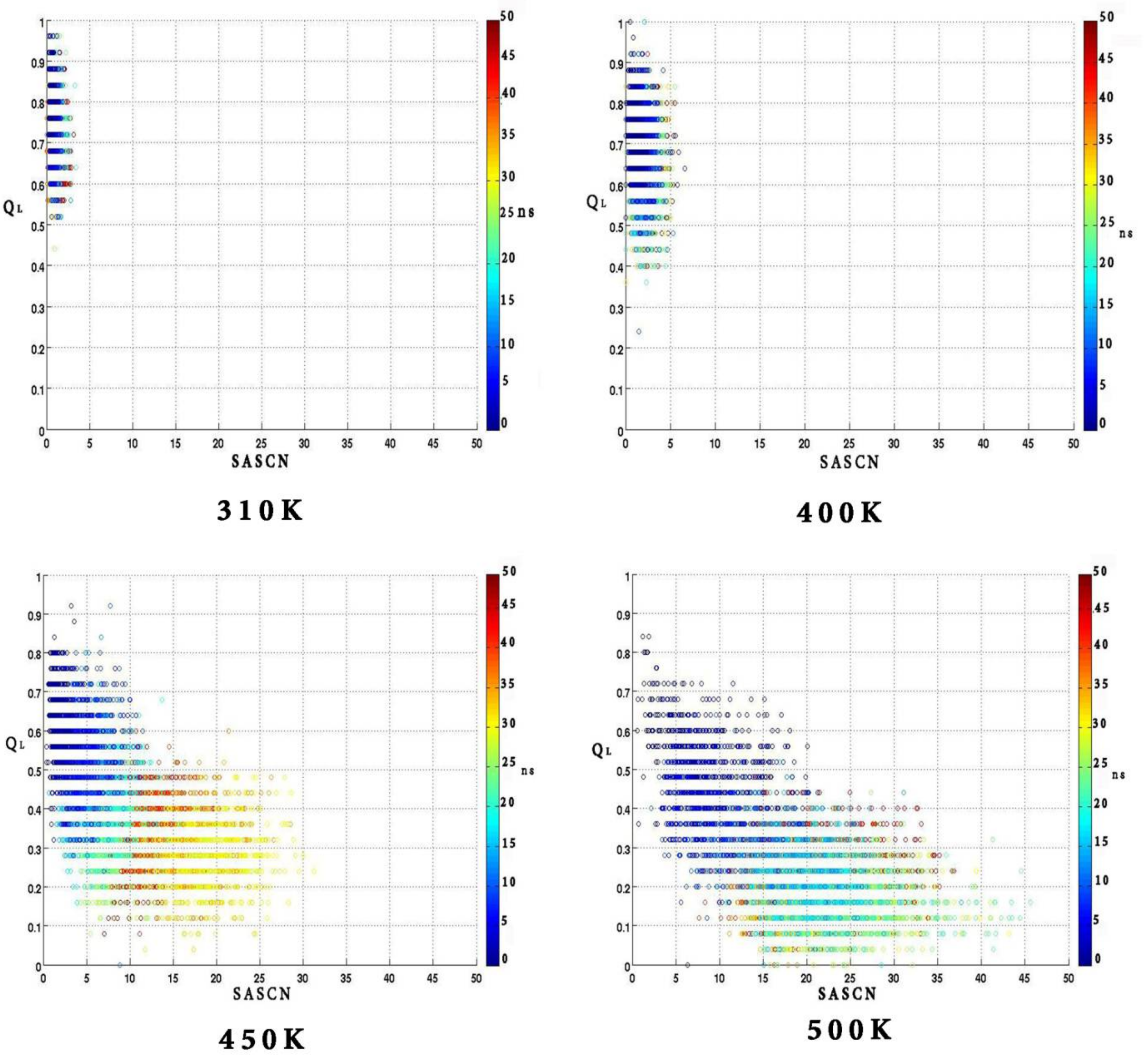

Figure P. Q_L-SASCN plots at different simulation temperatures. Fraction of native links (Q_L) v/s SASCN plots at a) 310 K b) 400 K c) 450 K and d) 500 K to identify the ‘Native-like’ , ‘DMG-like’, ‘WMG-like’ and the ‘TS’ regions. The color bar for the plotted points represents snapshots at time intervals during the course of the simulation.

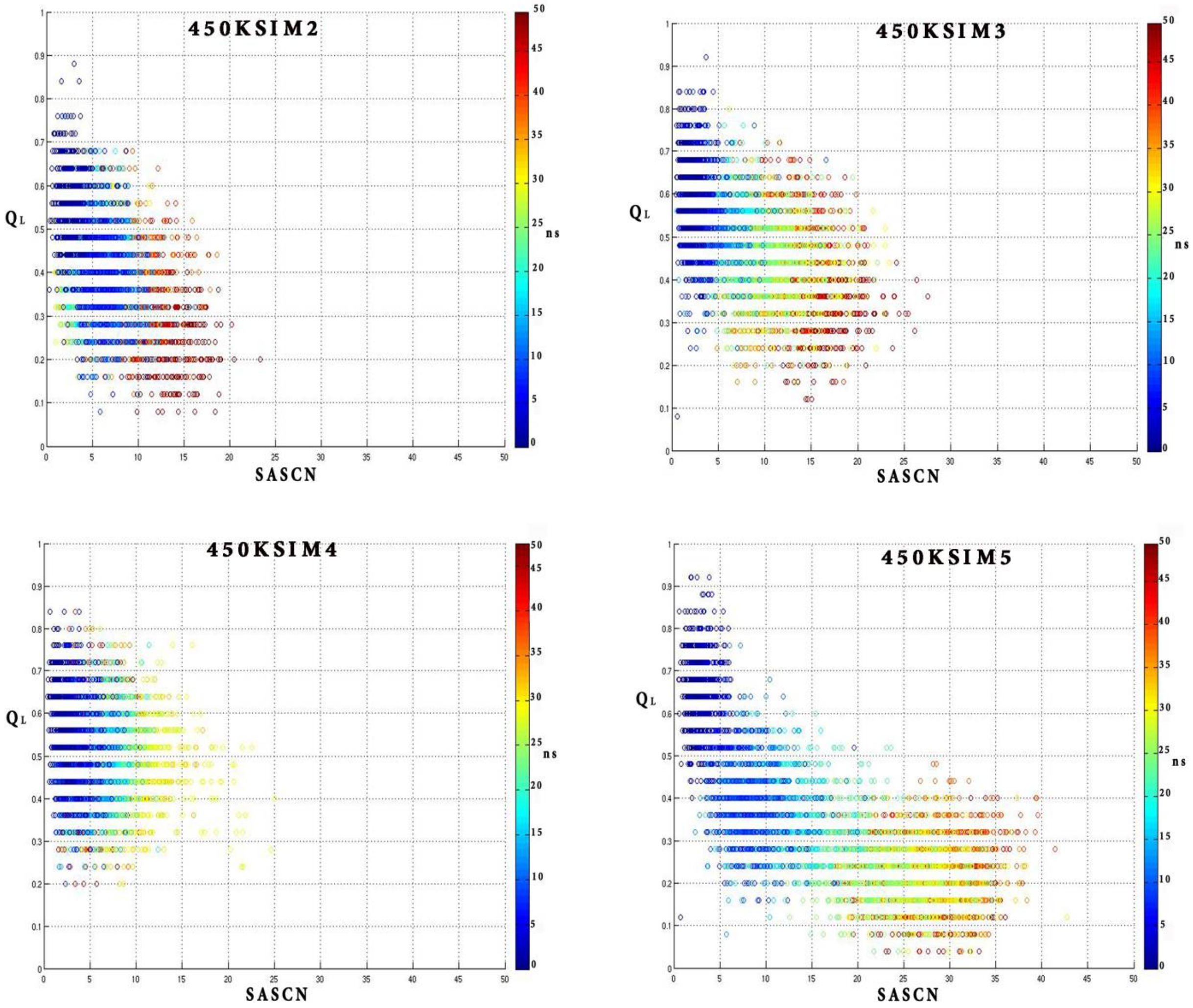

Figure Q. Q_L-SASCN plots at 450K (SIM2-SIM5). Fraction of native links Q_L-SASCN plots for 450 K a) 450KSIM2 b) 450KSIM3 c) 450KSIM4 and d) 450KSIM5 to identify the 'native-like' , 'DMG-like', 'WMG-like' and the 'TS' snapshots.

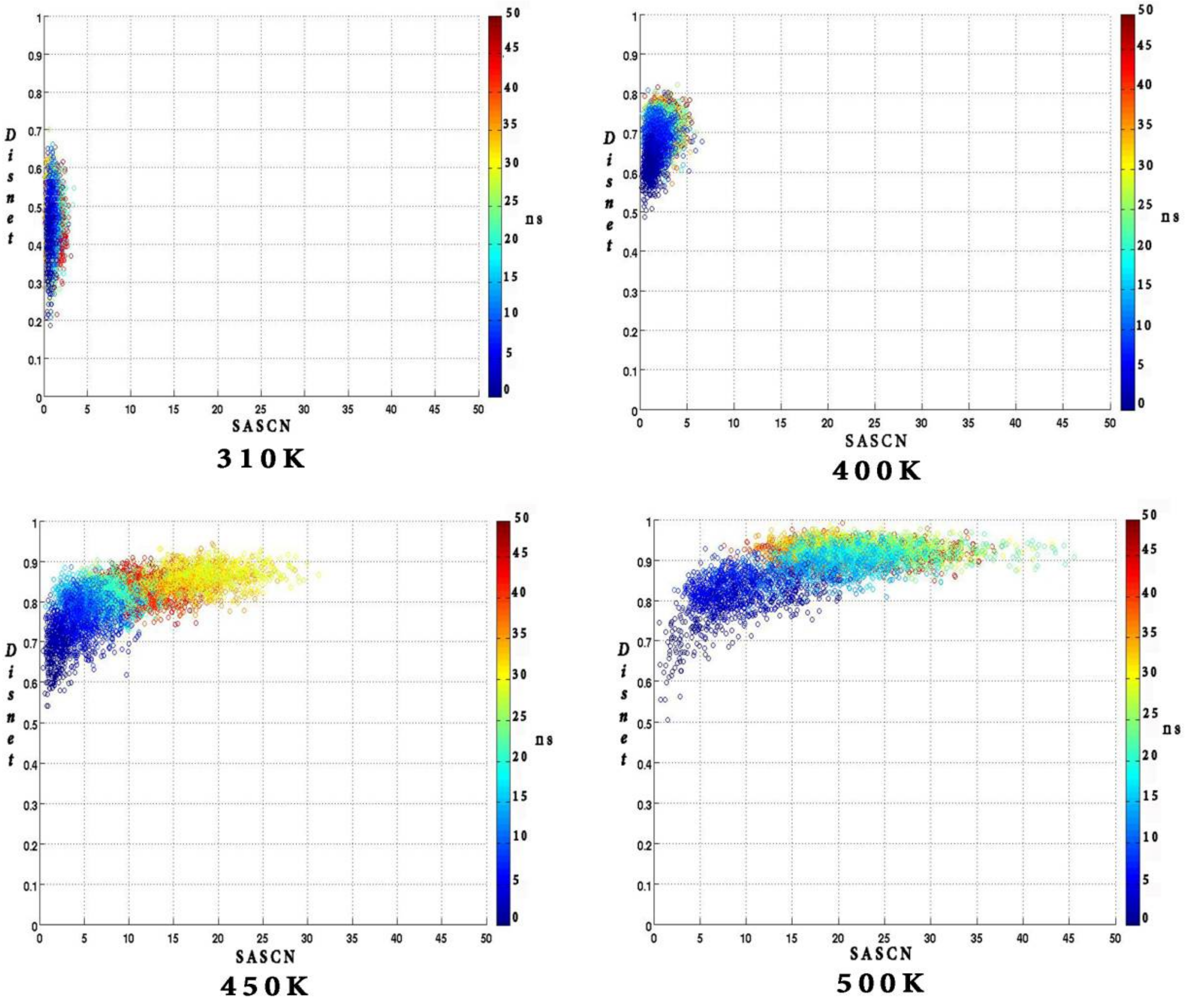

Figure R. *Disnet*-SASCN plots at different simulation temperatures. *Disnet*-SASCN plots at a) 310 K b) 400 K c) 450 K and d) 500 K to identify the ‘native , ‘DMG like’, ‘WMG like’ and the ‘TS’ regions.The color bar for the plotted points represents snapshots at time intervals during the course of the simulation

Supporting Tables

Table A. Set of contacts of S1 (core-core), S2 (helix1-core) and S3 (helix2-core), with the secondary structural elements in parentheses, S-Strands, H1-helix1, H2-helix2 and L-loops

S1	S2	S3
120 LEU(S)-151 PHE(S)	59 PHE(H1)-151 PHE(S)	85 PHE(S)-164 PHE(H2)
29 VAL(S)-47 LEU(S)	62 LEU(H1)-71 TYR(L)	85 ILE(S)-161VAL(H2)
122 MET(S)-151PHE(S)	29 VAL(S) -63 CYS(H1)	164 ILE(H2)-179 VAL(S)
31 PHE (S)-181 ILE(S)	55 THR(H1)-151 PHE(S)	
76 PHE(L)-85 ILE(S)	59 PHE (H1)-71 TYR(L)	
31 PHE(S)-45 ILE(S)	55 THR(H1)-122 MET(S)	
76 PHE(L)-181 ILE(S)	47 LEU(S)- 59 PHE(H1)	
71 TYR(L) -134 PHE(S)	59 PHE(H1)-134 PHE(S)	
31 PHE (S)-134 PHE(S)	31 PHE(S)-59 PHE(H1)	
31 PHE (S)-71 TYR(S)		
33 VAL(S) -179 VAL(S)		

Table B. Average Cα RMSD of simulations with all the residues (SIM_ALL) of LdCyp and with only the core residues (SIM_CORE) for other simulation sets (for temperatures 310,400,450,500 K) with the standard deviations in parentheses.

Simulations	<Cα RMSD> Å	
	All	Core
310KSIM2	1.44(0.07)	0.53(0.05)
310KSIM3	1.51(0.09)	0.57(0.06)
310KSIM4	1.56(0.07)	0.52(0.06)
310KSIM5	1.55(0.12)	0.52(0.06)
400KSIM2	2.17(0.10)	0.61(0.07)
400KSIM3	2.19(0.11)	0.60(0.08)
400KSIM4	2.16(0.15)	0.82(0.07)
400KSIM5	2.41(0.43)	0.67(0.08)
450KSIM2	3.95(0.49)	1.39(0.30)
450KSIM3	3.64(0.18)	1.60(0.10)
450KSIM4	2.90(0.26)	1.42(0.28)
450KSIM5	4.36(0.40)	1.63(0.30)
500KSIM2	5.42(0.51)	3.90(0.47)
500KSIM3	6.05(0.53)	3.81(0.29)
500KSIM4	6.23(1.01)	4.46(0.65)
500KSIM5	6.94(0.84)	3.71(0.48)

Table C. RMSF values of core residues, all residues and non-core residues with standard deviations in parentheses

Simulation Sets	<RMSF> Å Residues		
	All	Core	Non-Core
300KSIM1	0.63(0.35)	0.38(0.05)	0.67(0.36)
400KSIM1	1.17(0.65)	0.67(0.30)	1.25(0.81)
450KSIM1	1.72(0.79)	0.97(0.26)	1.85(1.10)
500KSIM1	2.47(1.35)	1.71(0.56)	2.60(1.41)

Table D in S1 File. <*Disnet*> values for different simulation temperatures for the 310,400,450 and 500 K calculated with native crystal structure (2HAQ) as baseline averaged over the entire simulation block of 50ns with the standard deviation given in parentheses.

Simulations	<*Disnet*>
310KSIM2	0.48(0.09)
310KSIM3	0.47(0.09)
310KSIM4	0.50(0.11)
310KSIM5	0.47(0.08)
400KSIM2	0.49(0.08)
400KSIM3	0.53(0.08)
400KSIM4	0.55(0.11)
400KSIM5	0.50(0.10)
450KSIM2	0.68(0.10)
450KSIM3	0.63(0.11)
450KSIM4	0.60(0.09)
450KSIM5	0.76(0.13)
500KSIM2	0.84(0.11)
500KSIM3	0.85(0.17)
500KSIM4	0.87(0.12)
500KSIM5	0.91(0.16)

Table E. Cross-Correlation values between Q and *Disnet* at different simulation temperatures (SIM1-SIM5)

Temperature (K)	Cross-correlation values between Q and *Disnet* Simulation Sets					Averaged Over 5 simulations (SIM1-SIM5)	Standard Deviation
	S IM1	SIM2	SIM3	SIM4	SIM5		
310	1017.8	999.2	1019.4	1052.4	989.7	1015.7	24.0
400	1108.7	1034.9	1104.8	1086.8	1060.9	1079.2	31.2
450	1046.5	1053.6	1116.5	1231.8	1201.4	1129.9	84.3
500	1273.8	1143.5	1086.0	1091.5	1008.2	1120.6	98.3

Table F in S1 File. Dlf and persf values averaged over the entire simulation block (between 2 – 50 ns) for the hydrophobic core of LdCyp at different simulation temperatures for (450KSIM2, 450KSIM3, 450KSIM4 and 450KSIM5). The standard deviations are given in parentheses.

Temperature (K)/Simulation Sets	<dlf>	<Persf>
450KSIM2	0.65(0.04)	0.55(0.11)
450KSIM3	0.60(0.04)	0.48(0.15)
450KSIM4	0.59(0.02)	0.42(0.05)
450KSIM5	0.60(0.02)	0.47(0.08)

S1 Movie: Movie Caption: “Unfolding of cyclophilin from Leishmania Donovani (LdCyp)”.
